# Mapping hemagglutinin residues driving antigenic diversity in H5Nx avian influenza viruses

**DOI:** 10.1128/jvi.00095-26

**Published:** 2026-04-30

**Authors:** Rebecca Daines, Jean-Remy Sadeyen, Pengxiang Chang, Munir Iqbal

**Affiliations:** 1 Avian Influenza and Newcastle disease group, The Pirbright Institute, Woking, United Kingdom; 2 Department of Pathobiology and Population Sciences, Royal Veterinary College 325262, Hatfield, United Kingdom; Cornell University Baker Institute for Animal Health, Ithaca, New York, USA

**Keywords:** antigenicity, antigenic cartography, avian influenza viruses, H5Nx, hemagglutinin, putative antigenic residues

## Abstract

Since its emergence in 1996, the H5 avian influenza virus (AIV) A/Goose/Guangdong/1/1996 (Gs/GD) hemagglutinin (HA) has evolved into over 30 genetically and antigenically distinct clades, including the widespread clade 2.3.4.4b. Vaccination is widely used in endemic regions to reduce poultry losses and zoonotic risk. However, the evolving antigenic diversity and global co-circulation of multiple clades challenge the protective efficacy of poultry vaccines with poor antigenic matching to field strains, resulting in immune escape and vaccine failure. This study aimed to improve vaccine seed selection by identifying HA epitopes contributing to inter-clade antigenic differences. Recombinant clade-representative viruses were generated using HA genes from circulating H5 AIVs via reverse genetics with A/Puerto Rico/8/1934 (PR8) internal and neuraminidase genes. Antigenic relationships were assessed using hemagglutination inhibition (HI) assays with homologous and heterologous chicken antisera. Antigenic cartography revealed a clear distinction of clade 2.3.4.4 from others and notable intra-clade diversity. Pairwise antigenic and genetic comparisons identified 48 putative antigenic residues. These were individually introduced into a candidate HA by site-directed mutagenesis, and antigenic influence was assessed by HI using sera raised against the non-mutated HA. Four residues R82K, A83T, T204I, and F229Y had significant antigenic effects, with three (R82K, T204I, and F229Y [H5 numbering]) being novel. These findings demonstrate that combining serology and *in silico* residue analysis can identify key antigenic determinants. This work highlights the need for precise antigenic matching in vaccine design and highlights the value of combining molecular and immunological tools to optimize vaccine seed selection against diverse and evolving H5 strains.

**IMPORTANCE:**

The continued evolution of H5 avian influenza viruses (AIVs), particularly the Gs/GD lineage, poses major challenges for poultry disease control and zoonotic risk mitigation. Vaccine effectiveness is undermined by antigenic drift and the co-circulation of diverse clades, often leading to mismatches between vaccine and field strains. This study addresses the critical need to improve vaccine strain selection by identifying hemagglutinin (HA) residues driving antigenic variation across H5 clades. Using recombinant viruses, antigenic cartography, hemagglutination inhibition assays, and mutagenesis, we pinpointed 48 key residues, with four R82K, A83T, T204I, and F229Y having major antigenic effects, including three novel markers. These findings advance our understanding of H5 antigenic evolution and provide a framework for predicting vaccine performance. By integrating molecular and serological data, our work informs rational vaccine seed strain selection, contributing to more broadly protective vaccines and improved control of H5 AIV in poultry, while reducing the risk of zoonotic transmission.

## INTRODUCTION

Low pathogenic avian influenza (LPAI) viruses circulate naturally in wild aquatic birds, particularly in species from the orders *Anseriformes* and *Charadriiformes* (1). These birds serve as reservoirs for all avian influenza virus (AIV) subtypes and typically show little to no clinical disease (1). Their migratory behavior drives global virus distribution, with occasional spillover of LPAI subtypes into domestic poultry populations. In poultry, these viruses can establish persistent circulation and cause mild to severe disease (2). Notably, highly pathogenic avian influenza (HPAI) viruses can emerge when LPAI H5 or H7 subtypes infect galliform poultry, acquiring mutations in the hemagglutinin (HA) that enable systemic infection, leading to increased severity and mortality (3). These HPAI strains can spill back into wild bird populations, often causing subclinical or less severe disease, and may be reintroduced into naïve poultry flocks (4, 5).

A novel outbreak of H5 HPAI with high morbidity was first detected in domestic geese in China in 1996, resulting in the emergence of the H5 HA A/Goose/Guangdong/1/1996 (Gs/GD) lineage (1). This strain differed from earlier, sporadic H5N1 HPAI viruses (6, 7). Since its emergence, the Gs/GD lineage has diversified into 10 genetically distinct clades (0–9), each with multiple subclades (8). Clade 2.3.4.4 was first identified in Asia between 2013 and 2014, following earlier reassortment events among clade 2.3.4 H5N1 viruses. This clade has since given rise to multiple H5Nx subtypes through reassortment with different neuraminidase (NA) genes and has become one of the dominant H5 lineages in outbreaks across Asia, Europe, the Middle East, and other regions. This clade further diversified into subclades 2.3.4.4a–h, initially named based on their geographical origins (9). September 2022 to February 2023 saw unprecedented international loss of domestic and wild bird populations from HPAI H5N1 of clade 2.3.4.4b, presenting as multiple reassortments, H5N1, N2, N4, N5, N6, and N8 (10). Most notably, the host range expanded into domestic and predatory mammals, aquatic porpoises, whales, and cohabiting semi-aquatic mammals, including seals, significantly increasing the risk of zoonotic potential.

Vaccination remains a key strategy to reduce both the socio-economic impacts of AIV infections and the zoonotic risk in endemic regions. However, the rapid genetic and antigenic evolution of AIVs, particularly H5Nx viruses from clade 2.3.4.4, complicates effective vaccine seed strain selection. Poor antigenic matching may result in suboptimal protective immunity or drive immune escape, accelerating viral evolution (11). Vaccine strain updates are typically based on hemagglutination inhibition (HI) assays that measure the cross-reactivity of vaccine-induced sera against circulating viruses (12). When cross-reactivity drops below a defined threshold, a new seed strain is selected to restore vaccine efficacy and reduce the risk of vaccine failure. While whole inactivated virus vaccines are favored for their low cost and scalability, they may quickly become antigenically outdated and fail to cover the breadth of circulating diversity. In response, *in silico* approaches, including synthetic antigen design, chimeric constructs, and multivalent formulations, have been developed to expand vaccine coverage but are often constrained by production complexity and time (13).

The antigenic diversity and co-circulation of H5Nx clades often confound efficient vaccination regimes or challenge accurate antigenic matching. Therefore, this research sought to adopt *in silico* methods to identify the residues driving antigenic disparity between the clades of H5Nx. The identification of these residues can not only aid our understanding of the antigenic variability of H5Nx AIVs but also direct more accurate and regionally targeted vaccine seed selection and alleviate the socio-economic burden faced by the poultry industry.

## RESULTS

### Genetic variability within and between H5Nx clade 2.3.4.4 subclades is significantly greater than in other clades

Representative candidate viruses were selected from both current and recently active clades of the Gs/GD lineage of H5Nx AIVs, based on WHO/FAO/OIE biannual reports on antigenic characterization and candidate vaccine virus (CVV) preparedness (8, 9). Virus selection was guided by these reports as well as the revised nomenclature established in 2015 (14). However, due to the rapid and ongoing antigenic diversification of clade 2.3.4.4, nomenclature updates have become outdated. Therefore, representatives from this clade were additionally selected based on WHO and Centers for Disease Control and Prevention (CDC) risk assessment reports and current CVVs (10, 15).

**Fig 1:**
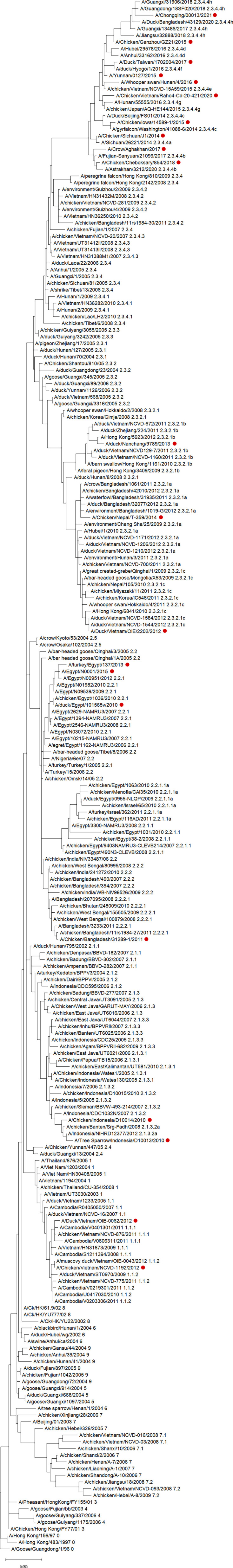
Maximum-likelihood phylogenetic tree showing evolutionary relationships among H5Nx HA sequences from Gs/Gd lineage, including clades 1–9, without 2.3.4.4. Multiple virus strains branch into distinct groups with selected sequences highlighted in red. Tree constructed using MEGAX using JTT + G model selected for best fit with a fixed rate of heterogeneity across sites and 90% deletion criteria (18).

Full-length HA sequences were retrieved from public databases, including NCBI (16) and Global Initiative on Sharing All Influenza Data (GISAID) (17). Sequences were trimmed to the mature open reading frame, aligned, and quality checked. A maximum-likelihood phylogenetic tree was constructed using the GTR + G substitution model, selected based on the lowest Bayesian information criterion (BIC) among best-fit models; the tree was rooted to the clade 0 Gs/GD strain, with the assumption of equal evolutionary rates across lineages (Fig. 1).

In all, 22 viruses were selected based upon amino acid genetic consensus within the monophyletic branch of each clade, annotated according to the WHO/OIE/FAO-assigned reference strains within the established nomenclature system (10, 14), spanning clades 1.1.1 to 2.3.4.4h (Table 1). For clarity, strain names are hereafter referred to by shortened designations.

**TABLE 1:** Representative H5Nx strains with associated clade and subtype chosen for this study^*a*^

Strain name	Short name	Clade	Subtype	Accession number
A/Duck/Vietnam/OIE-0062/2012	VMN12a	1.1.1	H5N1	EPI_ISL_121358
A/Chicken/Vietnam/NCVD-1192/2012	VMN12b	1.1.2	H5N1	EPI_ISL_136197
A/Tree_Sparrow/Indonesia/D10013/2010	IDN10a	2.1.3.2a	H5N1	EPI_ISL_93322
A/Chicken/Indonesia/D10014/2010	IDN10b	2.1.3.2b	H5N1	EPI_ISL_93252
A/Egypt/N0001/2015	EGY15	2.2.1	H5N1	KP864432.1*
A/Duck/Egypt/101565v/2010	EGY10	2.2.1	H5N1	EPI_ISL_80439
A/Turkey/Egypt/137/2013	EGY13	2.2.1.2	H5N1	KJ522737.1*
A/Chicken/Bangladesh/31289-1/2011	BGD11	2.2.2.1	H5N1	EPI_ISL_141271
A/Chicken/Nepal/T-359/2014	NPL14	2.3.2.1a	H5N1	EPI_ISL_161008
A/Duck/Nanchang/9789/2013	CHN13	2.3.2.1b	H5N1	EPI_ISL_181417
A/Duck/Vietnam/OIE-2202/2012	VMN12c	2.3.2.1c	H5N1	EPI_ISL_133016
A/Chicken/Sichuan/J1/2014	CHN14	2.3.4.4a	H5N6	EPI_ISL_202554
A/Chicken/Cheboksary/854/2018	RUS18	2.3.4.4b	H5N8	EPI_ISL_320957
A/Crow/Aghakhan/2017	IRN17	2.3.4.4b	H5N8	EPI_ISL_380991
A/Chicken/Iowa/14589-1/2015	USA15	2.3.4.4c	H5N2	EPI_ISL_190464
A/Chicken/Ganzhou/GZ21/2015	CHN15a	2.3.4.4d	H5N6	EPI_ISL_244536
A/Anhui/33162/2016	CHN16a	2.3.4.4d	H5N6	EPI_ISL_284650
A/Yunnan/0127/2015	CHN15b	2.3.4.4d	H5N6	EPI_ISL_195294
A/Duck/Taiwan/1702004/2017	TWN17	2.3.4.4e	H5N6	EPI_ISL_248634
A/Whooper swan/Hunan/4/2016	CHN16b	2.3.4.4f	H5N6	EPI_ISL_400489
A/Chicken/Vietnam/Raho4-Cd-20-421/2020	VMN20	2.3.4.4g	H5N6	EPI_ISL_1379443
A/Chongqing/00013/2021	CHN21	2.3.4.4h	H5N6	EPI_ISL_1081369

^*a*^ Short names adopt ISO country three-letter codes with the year of isolation. Strains sourced from GISAID except for (*) sourced from NCBI.

**Fig 2:**
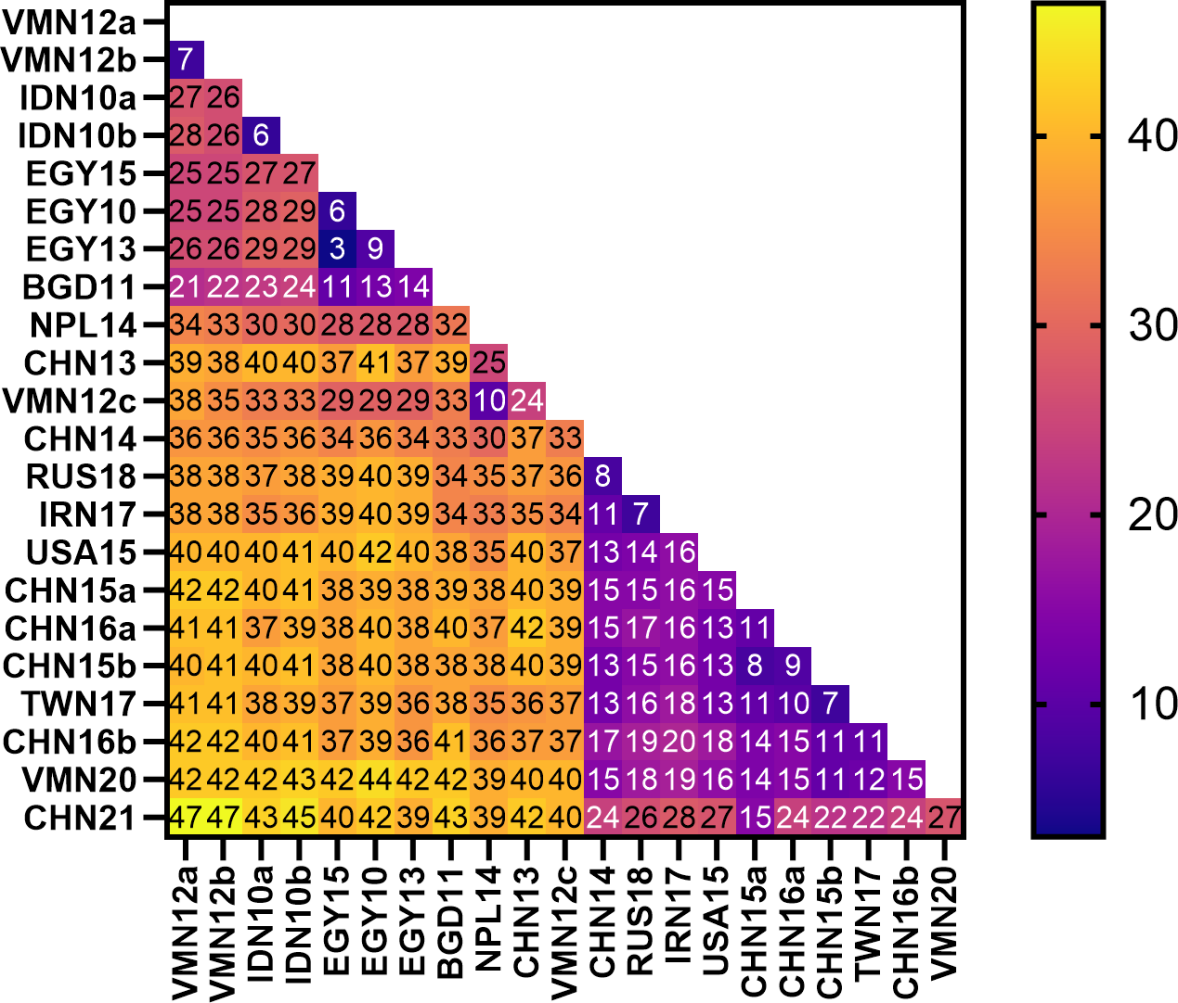
Heatmap of pairwise amino acid variances of the mature HA sequence between strains. Pairwise differences were compiled in MEGA X and presented as a heatmap using GraphPad (19).

To further investigate the extent of genetic diversity among the selected strains and support downstream analyses, pairwise amino acid differences across the mature HA sequences (excluding the signal peptide) were calculated (Fig. 2). A heatmap was generated to visualize these variations, with color gradients ranging from purple (low divergence) to yellow (high divergence), representing the degree of pairwise sequence dissimilarity.

Amino acid variation among the selected strains ranged from 3 to 47 differences. Lower pairwise variation was generally observed between viruses from closely related clades and similar geographic origins; three differences were observed between EGY13 and EGY15 (clades 2.2.1.2 and 2.2.1, respectively), and six differences between IDN10a and IDN10b (2.1.3.2a and 2.1.3.2b) and EGY10 and EGY15 (clades 2.2.1), and seven differences between VMN12a and VMN12b (1.1.1 and 1.1.2) as well as TWN17 and CHN15b (clades 2.3.4.4e and 2.3.4.4d, respectively). In contrast, the highest pairwise divergence (47 differences) was observed between CHN21 (clade 2.3.4.4h) and VMN12a and VMN12b (clades 1.1.1 and 1.1.2, respectively), reflecting the substantial genetic evolution and distance. Within clade 2.3.4.4 itself, intraclade variation ranged widely from 7 to 28 differences. The lowest variation was between CHN15a and CHN15b (both 2.3.4.4d) and RUS18 and IRAN17 (both 2.3.4.4b), with seven variances, while comparisons between CHN15b (2.3.4.4d) and TWN17 (2.3.4.4e) also showed limited divergence with eight variances. The greatest variation within clade 2.3.4.4 was consistently associated with CHN21 (2.3.4.4h), which differed by at least 21 amino acids from all other 2.3.4.4 representatives.

Antigenic diversity in AIVs is predominantly driven by amino acid changes within the HA1 region of the HA protein, located upstream of the proteolytic cleavage site. This region encompasses the receptor-binding domain (RBD), a critical target for neutralizing antibodies. Due to its immunological importance, the HA1 region was extracted from each HA sequence for focused analysis of genetic variation and used in subsequent antigenic mapping (Table 2).

**TABLE 2:** Amino acid variance of HA1 between the representative clade strains^*a*^

Strain	Clade	Residue position																							
		35	40	43	47	54	66	71	82	83	94	97	114	115	118	119	120	123	124	126	127	129	133	136	138
RUS18	2.3.4.4b	K	K	D	V	N	M	I	R	A	S	N	I	L	P	K	S	P	N	E	T	L	A	P	Q
VMN12a	1.1.1	.	.	.	I	D	.	.	K	.	V	D	.	Q	.	.	.	.	.	.	A	.	.	.	.
VMN12b	1.1.2	.	.	.	.	D	.	.	K	.	V	D	.	Q	.	.	.	.	S	.	A	.	.	.	.
IDN10a	2.1.3.2a	R	.	.	.	D	.	.	K	.	.	D	.	Q	.	.	.	S	D	.	A	S	S	.	L
IDN10b	2.1.3.2b	.	.	N	.	D	.	.	K	.	.	D	.	Q	H	.	.	S	D	.	A	S	S	.	L
EGY15	2.2.1	.	.	N	.	D	.	L	K	I	N	D	.	Q	.	.	D	S	D	.	A	del	S	.	.
EGY10	2.2.1	.	.	N	.	D	.	L	K	I	N	D	.	Q	.	.	N	S	D	.	A	del	S	.	.
EGY13	2.2.1.2	.	.	.	.	D	.	L	K	I	N	D	.	Q	.	.	D	S	D	.	V	del	.	.	.
BGD11	2.2.2.1	.	.	.	.	D	.	L	K	I	.	D	.	Q	.	.	.	S	D	.	A	S	S	.	.
NPL14	2.3.2.1a	.	.	.	.	.	.	.	K	.	N	D	.	R	.	.	D	S	D	.	A	.	.	.	.
CHN13	2.3.2.1b	.	.	.	.	.	.	T	K	T	.	D	.	R	.	Q	D	.	D	.	A	.	.	S	.
VMN12c	2.3.2.1c	.	.	.	.	.	L	.	K	.	N	D	.	Q	.	.	D	S	D	.	A	.	.	S	.
CHN14	2.3.4.4a	.	.	.	.	.	.	.	.	.	N	D	.	.	.	.	.	T	.	.	.	.	.	.	.
IRN17	2.3.4.4b	.	.	.	.	.	.	.	K	.	.	D	.	.	.	.	.	.	.	.	.	.	.	.	L
USA15	2.3.4.4c	.	.	.	.	.	I	.	.	.	T	D	T	.	.	R	.	.	.	.	.	.	.	.	.
CHN15a	2.3.4.4d	.	.	.	.	.	.	.	.	.	N	D	T	.	.	.	.	.	.	del	.	S	.	.	.
CHN16	2.3.4.4d	.	.	.	.	.	.	.	.	.	N	D	T	.	.	.	.	.	.	del	.	P	.	.	L
CHN15b	2.3.4.4d	.	R	.	.	.	.	.	.	.	N	D	T	.	.	.	.	.	.	del	.	S	.	.	.
TWN17	2.3.4.4e	.	R	.	.	.	.	.	.	T	N	D	T	.	.	.	.	.	.	.	.	del	.	.	.
CHN16	2.3.4.4f	.	R	.	.	.	.	.	.	.	N	D	T	.	.	.	.	.	.	.	.	.	.	.	.
VMN20	2.3.4.4g	.	R	.	.	.	.	.	.	.	N	D	T	.	.	.	.	.	.	.	.	.	.	Q	.
CHN21	2.3.4.4h	.	.	.	.	.	.	.	.	.	N	D	T	Q	.	.	E	S	.	T	.	del	.	.	.

		140	151	154	155	156	161	168	169	184	185	195	198	204	207	210	223	229	234	235	275	277	282	297	309
RUS18	2.3.4.4b	T	I	N	D	A	K	N	R	A	E	T	I	T	Q	V	R	F	K	P	N	K	V	L	N
VMN12a	1.1.1	R	.	.	S	T	.	G	Q	.	A	.	.	.	.	T	S	.	.	.	S	.	M	.	.
VMN12b	1.1.2	Q	.	.	S	T	.	.	Q	.	A	.	.	.	.	T	S	.	.	.	.	.	M	.	T
IDN10a	2.1.3.2a	S	.	.	S	T	.	.	Q	E	A	.	.	.	.	.	S	.	.	.	.	.	M	I	.
IDN10b	2.1.3.2b	S	.	.	S	T	.	.	Q	E	A	.	.	.	.	.	S	.	.	.	.	.	M	.	D
EGY15	2.2.1	R	T	.	.	.	.	.	Q	.	A	.	.	.	.	.	S	.	.	S	.	.	I	.	.
EGY10	2.2.1	R	T	D	N	.	.	.	Q	.	T	.	.	.	.	.	S	.	.	S	.	.	I	.	.
EGY13	2.2.1.2	R	T	.	.	.	.	.	Q	D	A	.	.	.	.	.	S	.	.	S	.	.	I	.	.
BGD11	2.2.2.1	R	.	.	.	.	.	.	Q	.	A	.	.	.	L	.	S	.	.	.	.	.	.	.	.
NPL14	2.3.2.1a	N	.	D	N	T	.	.	Q	E	A	.	.	.	.	.	S	.	.	.	.	R	I	.	.
CHN13	2.3.2.1b	N	L	.	.	.	T	.	K	E	A	.	V	.	.	.	.	.	.	.	S	R	M	.	.
VMN12c	2.3.2.1c	N	.	D	N	.	.	.	.	E	A	.	.	.	.	.	S	.	.	.	.	R	I	.	.
CHN14	2.3.4.4a	.	.	.	.	.	.	.	Q	.	A	.	.	.	.	.	.	.	.	.	.	.	I	.	.
IRN17	2.3.4.4b	M	.	.	.	.	.	.	.	.	.	.	.	.	.	.	.	.	.	.	.	R	.	.	.
USA15	2.3.4.4c	A	.	.	.	.	.	.	.	.	A	A	V	.	.	.	S	.	.	.	.	.	I	.	.
CHN15a	2.3.4.4d	M	T	.	.	.	.	.	.	.	.	.	V	.	.	.	.	.	R	.	.	.	I	.	.
CHN16	2.3.4.4d	M	T	.	.	S	.	.	.	.	A	.	V	I	.	.	S	.	.	.	.	.	I	F	.
CHN15b	2.3.4.4d	M	T	.	.	.	.	Y	.	.	A	.	V	.	.	E	.	.	.	.	.	.	I	.	.
TWN17	2.3.4.4e	V	T	.	.	.	.	.	G	E	A	.	V	.	.	.	Q	.	.	.	.	.	I	.	.
CHN16	2.3.4.4f	M	T	.	.	.	.	.	.	E	A	.	V	.	.	.	.	.	.	S	.	.	I	.	.
VMN20	2.3.4.4g	M	.	.	.	.	E	.	S	.	A	.	V	.	.	.	.	Y	.	.	.	.	I	.	.
CHN21	2.3.4.4h	V	T	.	.	.	.	.	K	V	.	.	V	.	.	.	.	.	R	.	.	.	I	.	.

^*a*^ Residue positions with variances between the strains are shown in comparison to the RUS18 2.3.4.4b strain. Deletions indicated with (del), identical residues to RUS18 indicated with (.). Mature H5 numbering used (20, 21). Amino acids are stated in their single-letter code.

A total of 48 amino acid variations were identified within the mature HA1 region (excluding the signal peptide and cleavage site) of the selected strains. Of these, 21 positions (43.75%) exhibited two amino acid variants, another 21 positions (43.75%) showed three variants, and the remaining six positions (12.5%) had four or more variants when compared to the RUS18 reference sequence. The most variable sites, residues 94, 120, 129, 140, 169, and 184 (H5 numbering used henceforth), exhibited four, four, four, eight, five, and four amino acid variations, respectively.

**Fig 3:**
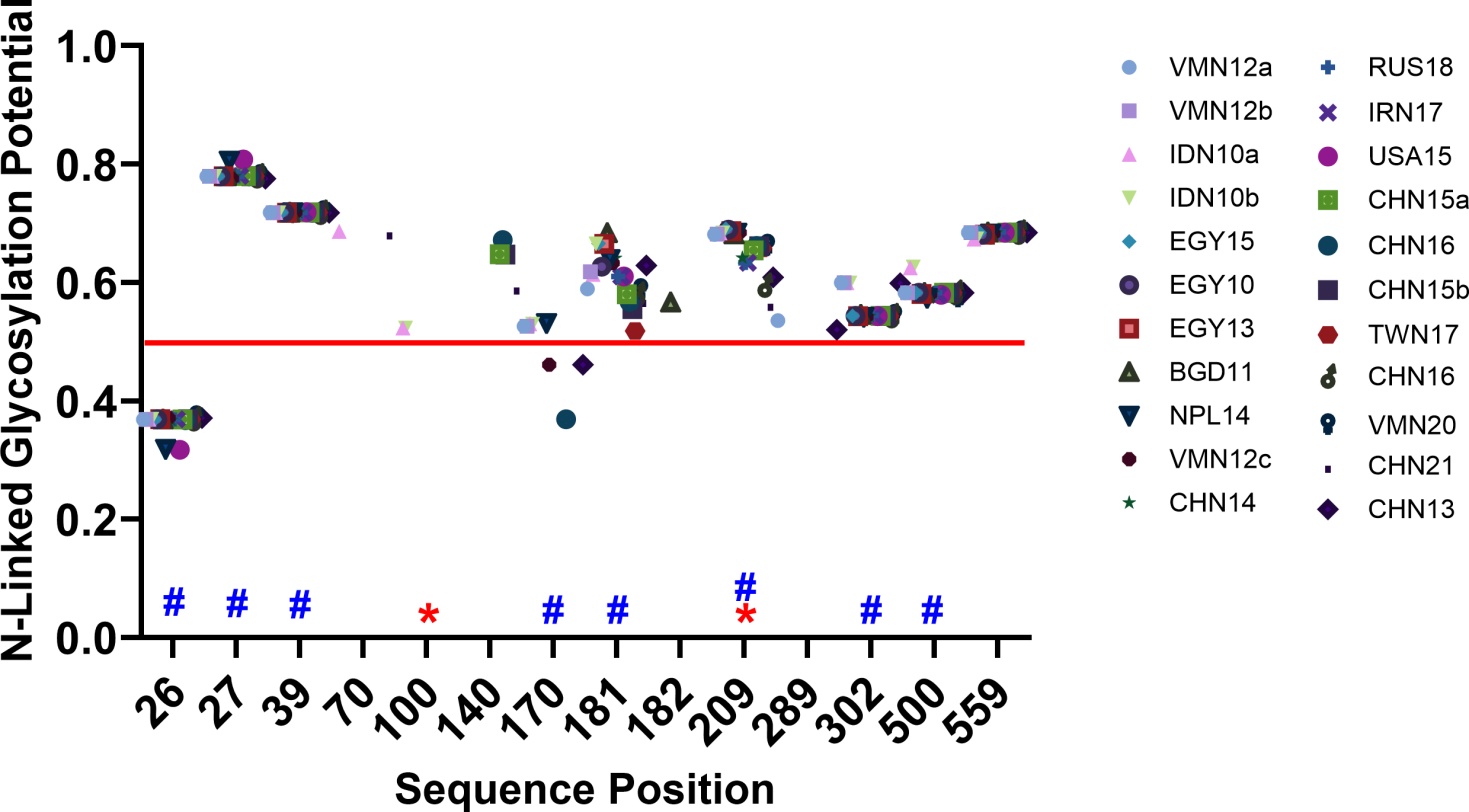
N-linked glycosylation prediction of study HA sequences. The “potential” score is the mean output of nine neural networks. Any symbol crossing the default threshold of 0.5 (red line) represents a predicted glycosylated site (if it occurs in the required sequon Asn-X-Ser/Thr without proline at X). Sequons with Pro present but achieved a positive confidence are indicated with (*). Conserved/frequently predicted sequons are indicated with (#). Mature H5 numbering used. Prediction run using NetNGlyc (22).

As glycosylation of HA can also influence antigenic diversity, sequences were analyzed for potential N-linked glycosylation using the NetNGlyc server (22) (Fig. 3). Glycan sites were predicted based on the presence of the canonical sequon Asn-X-Ser/Thr (where X is any amino acid except proline), with a confidence threshold of 0.5 (mean score from nine neural networks). No O-linked or C-linked glycosylation sites were identified.

Of the 14 predicted N-linked glycosylation sites which span the length of the HA sequences, sites 26, 27, 39, 181, 302, 500, and 559 are conserved across all strains and are known to be heavily conserved cross-clade. Sites 100 and 209, although above the threshold of predictive confidence, were not deemed N-glycans due to the presence of proline at X the Asn-X-Ser/Thr sequon. Site 182 was unique to BGD11, site 70 to IND10a and CHN21, site 289 to VNM12a and CHN13, and site 140 to CHN15a, CHN16a, CHN15b, and CHN21.

### All subclades within clade 2.3.4.4 are significantly antigenically distinct from other co-circulating or recently circulating clades and exhibit considerable intra-clade diversity

To identify correlates of antigenicity among clades, the selected strains were assessed for cross-reactive neutralizing antibody responses by measuring their ability to inhibit the binding of heterologous strains. Recombinant viruses were generated using reverse genetics (RG), incorporating the HA gene from each selected strain and the neuraminidase (NA) and internal genes of A/Puerto Rico/8/1934 (PR8). HA sequences were modified to remove the polybasic cleavage site and adapt non-coding regions for compatibility with the pHW2000 eight-plasmid bidirectional expression system. Polyclonal antisera were raised in chickens vaccinated with inactivated, concentrated, and adjuvanted recombinant viruses. Per strain, four to ten birds were inoculated at 1 day of age and boosted at 7 days post-vaccination. Serum samples were collected every 7 days, and antibody titers were assessed by HI assay. The final bleed at 42 days post-vaccination was used for cross-strain antigenic comparisons (Table 3). HI assays were additionally validated by microneutralization (MN) tests, with a sample of HI titers compared to MN titers (Fig. S1).

**TABLE 3:** Geometric mean titers (GMTs) of hemagglutination inhibition assays (log_2_) using homologous (bold) and heterologous whole virus antisera raised in chickens^*a*^

Strain name		Antisera																					
		NPL14	BGD11	IDN10a	CHN15b	USA15	VMN12a	EGY13	EGY15	EGY10	CHN13	VMN12b	IDN10b	VMN12c	CHN15a	RUS18	IRN17	CHN14	CHN15b	TWN17	CHN16	VMN20	CHN21
Virus	NPL14	**8.4**	6.3	6.8	3.1	3.5	7.2	7.3	5.7	7.1	8.2	3.3	7.9	9.2	4.7	3.1	4.7	4.6	2.7	2.3	3.7	4.4	4.0
	BGD11	4.7	**9.6**	5.9	2.6	5.3	9.1	8.6	7.4	7.7	5.6	7.5	7.1	7.5	6.2	5.4	6.7	7.3	4.2	4.1	5.2	5.8	5.4
	IDN10a	5.2	5.9	**10.8**	4.3	3.9	6.3	5.9	5.1	6.6	5.5	6.4	12.0	8.5	6.0	6.2	8.3	6.3	4.1	3.7	5.2	6.5	4.9
	CHN15b	2.7	3.9	5.6	**9.7**	4.6	3.0	3.7	3.0	4.2	4.4	1.4	4.9	2.8	11.5	4.6	6.3	6.7	9.3	7.7	4.7	5.8	7.9
	USA15	5.0	7.2	7.1	6.9	**11.3**	5.0	7.1	4.4	5.7	5.2	7.7	7.8	6.8	11.3	10.7	11.5	11.9	8.6	9.3	11.0	11.7	5.0
	VMN12a	3.6	6.1	5.9	1.2	3.1	**10.8**	6.6	4.4	5.4	5.0	9.2	6.6	6.7	5.5	3.4	4.9	4.1	3.1	1.3	3.1	4.0	2.5
	EGY13	4.5	5.0	4.3	1.4	2.2	4.2	**8.8**	7.2	8.4	5.7	3.6	5.7	7.6	3.8	2.0	2.4	2.9	2.9	1.6	2.9	3.0	4.1
	EGY15	5.8	7.0	5.2	2.8	3.2	6.4	9.6	**9.3**	10.0	6.9	5.5	6.9	8.2	5.7	2.8	3.7	4.5	3.7	1.4	3.2	4.9	5.7
	EGY10	4.3	6.1	5.1	2.3	3.1	5.4	8.5	7.8	**9.8**	5.1	5.6	6.0	7.8	6.3	3.3	3.1	5.4	3.1	2.2	2.9	4.3	5.2
	CHN13	6.6	2.8	4.8	1.1	1.2	3.7	4.7	2.8	4.7	**10.4**	2.4	4.5	8.1	2.4	0.8	1.3	2.6	1.0	1.0	2.5	3.4	2.4
	VMN12b	3.4	4.1	5.2	1.4	2.5	9.7	4.3	3.9	5.3	4.6	**8.5**	6.2	4.9	5.6	6.3	7.3	5.7	4.5	2.3	4.1	5.0	3.8
	IDN10b	5.1	5.7	10.7	3.2	5.1	5.2	5.6	5.3	6.1	5.7	5.2	**12.0**	8.3	4.3	2.0	8.4	6.1	4.0	3.2	5.6	6.3	3.8
	VMN12c	8.0	5.8	5.6	1.2	1.4	6.4	5.5	5.0	7.5	7.8	10.0	7.7	**11.5**	6.2	5.0	5.7	4.6	2.5	1.4	3.6	4.8	3.8
	CHN15a	4.1	4.9	6.4	8.1	3.8	4.1	3.9	3.2	5.6	4.0	3.8	6.3	4.8	**10.5**	6.7	6.9	7.7	8.5	7.2	6.4	8.0	8.7
	RUS18	5.4	8.8	10.3	9.8	12.0	9.1	7.6	7.7	10.1	7.5	8.5	10.8	7.1	10.1	**9.5**	10.7	11.0	9.3	9.0	11.0	10.7	7.5
	IRN17	4.0	5.8	7.5	6.6	8.8	6.2	6.2	5.1	7.7	5.8	6.6	9.1	5.5	7.7	8.2	**9.9**	9.0	7.2	7.2	9.5	10.1	6.5
	CHN14	3.6	6.0	6.2	4.8	7.6	4.3	5.6	3.5	6.0	4.3	4.9	6.8	4.9	7.3	6.4	8.7	**11.8**	7.6	6.8	9.5	9.8	6.5
	CHN15b	3.4	4.4	6.0	7.9	5.3	4.7	3.8	4.2	6.1	3.6	4.6	6.6	4.7	9.0	3.8	6.7	8.6	**9.6**	7.6	6.7	7.7	6.5
	TWN17	2.6	4.1	5.1	5.9	6.6	3.1	4.6	2.7	4.8	3.1	4.2	5.8	4.5	7.1	4.6	6.7	10.1	8.6	**9.0**	7.9	7.5	7.1
	CHN16	2.8	4.8	5.3	4.6	7.0	4.9	4.7	2.4	5.0	4.3	4.2	7.1	5.2	6.5	6.4	8.6	9.6	6.8	7.1	**11.2**	8.8	5.6
	VMN20	2.7	4.2	5.4	4.6	6.1	3.7	4.0	3.0	5.5	3.7	4.5	6.1	4.5	7.3	6.3	8.3	11.7	6.9	6.3	8.4	**10.6**	6.2
	CHN21	3.1	4.0	5.0	5.7	1.6	4.6	4.3	3.4	5.7	3.7	4.2	5.5	4.5	7.5	3.0	5.2	6.8	6.8	4.6	5.2	6.3	**10.7**

^*a*^ Columns and rows represent sera and viral antigens, respectively. Confidence intervals are displayed in Table S2 and Wilcoxon test statistic two-pair *P*-values are displayed in Table S6.

Geometric mean titers (GMTs) were calculated using data from four to ten biological replicates (individual bird sera) and three technical replicates per strain. A titer of ≥5 log₂ (equivalent to 32 hemagglutinating units [HAU]) is considered protective (23–25). All homologous titers (i.e., where the test antigen matched the immunizing strain) exceeded this threshold substantially, ranging from 8.4 to 12 log₂ (384–4,096) HAU. As anticipated, heterologous titers varied widely. Several were below the protective threshold, highlighting limited cross-protection between certain clades. Interestingly, RUS18 (clade 2.3.4.4b) and USA15 (clade 2.3.4.4c) were the most broadly reactive strains, with their HA inhibited by all heterologous sera tested, showing titers up to 10.7 and 12 log₂ (2,432 and 3,896 HAU), respectively. In contrast, CHN13 was the most antigenically distinct virus, showing cross-reactive inhibition with sera from only four heterologous viruses: NPL14, EGY13, EGY10, and IDN10a.

Although most reciprocal HI titers between antigen/antiserum pairs correlated well, some showed considerable discrepancies. For instance, sera raised against RUS18 tested against the CHN15a virus yielded titers of 6.7 log₂ (109 HAU), whereas CHN15a sera against RUS18 produced a markedly higher titer of 10.1 log₂ (1,728 HAU). A total of 42 antigen/antiserum pairs exhibited differences greater than 3 log₂, with the majority associated with RUS18 (16 pairs), USA15 (11 pairs), and CHN13 (six pairs). This may be due to glycan presentation differentials, as previously mentioned, and epitope exposure/shielding.

**Fig 4:**
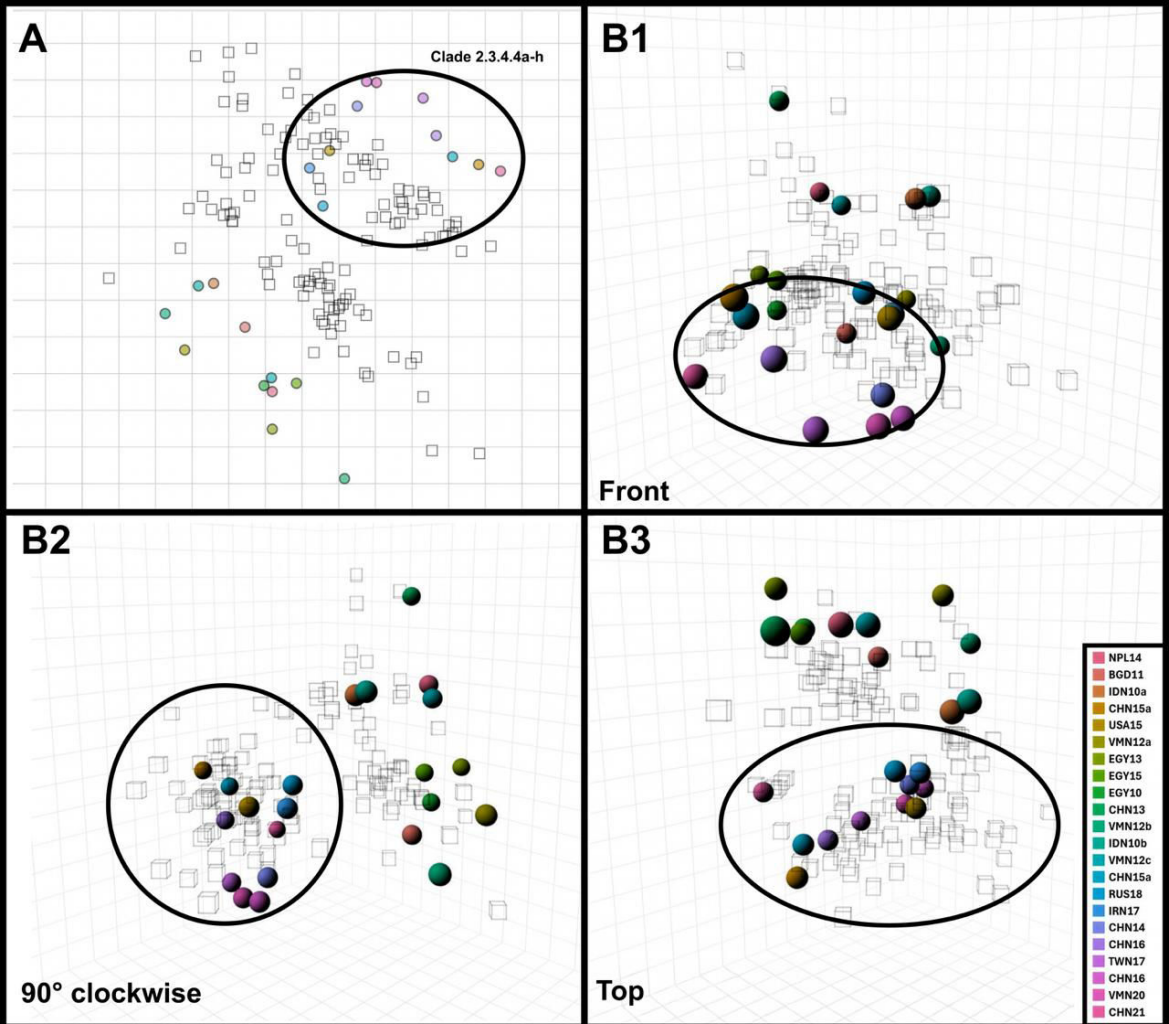
Antigenic cartography visualizing serological relationships (GMTs) from Table 3. Maps plotted in two (**A**) and three dimensions (**B1-3**). Circles/spheres are antigens, colored according to the key within B3; squares/cubes are sera. The grid plot represents one antigenic unit per square, that is, 2 log_2_ HI titer. The 2.3.4.4 clade is circled to indicate its distinction. Maps were constructed in RStudio, using the Racmacs package.

To visualize antigenic cross-reactivity among clades, an antigenic map was generated using the GMTs from Table 3. Antigens and sera were plotted in both two- (2D) and three-dimensional (3D) spaces, where spatial proximity reflected antigenic similarity, and closer points indicated greater cross-reactivity. Initial mapping and optimization were performed using ACMACS (26), followed by refinement and plotting in RStudio with the Racmacs package. An optimal minimum column basis (OMCB) of 128 and 500 optimization runs were applied to minimize error and stress. Additional relaxation and randomization through dimensional annealing confirmed the robustness of the final maps (Fig. 4).

The 2D and 3D antigenic maps clearly distinguished clade 2.3.4.4, which was visually segregated from the other clades. The antisera (represented as squares/cubes) from clade 2.3.4.4 largely clustered around the antigens (circles/spheres) of the same clade, whereas antisera from other clades were positioned more centrally on the map, situated between the 2.3.4.4 cluster and the remaining clade viruses. Additionally, the distribution of viruses within clade 2.3.4.4 revealed substantial intra-clade antigenic diversity.

### Comparative analysis of genetic and antigenic diversity highlighted putative antigenic residues driving variability between H5Nx clades

**Fig 5:**
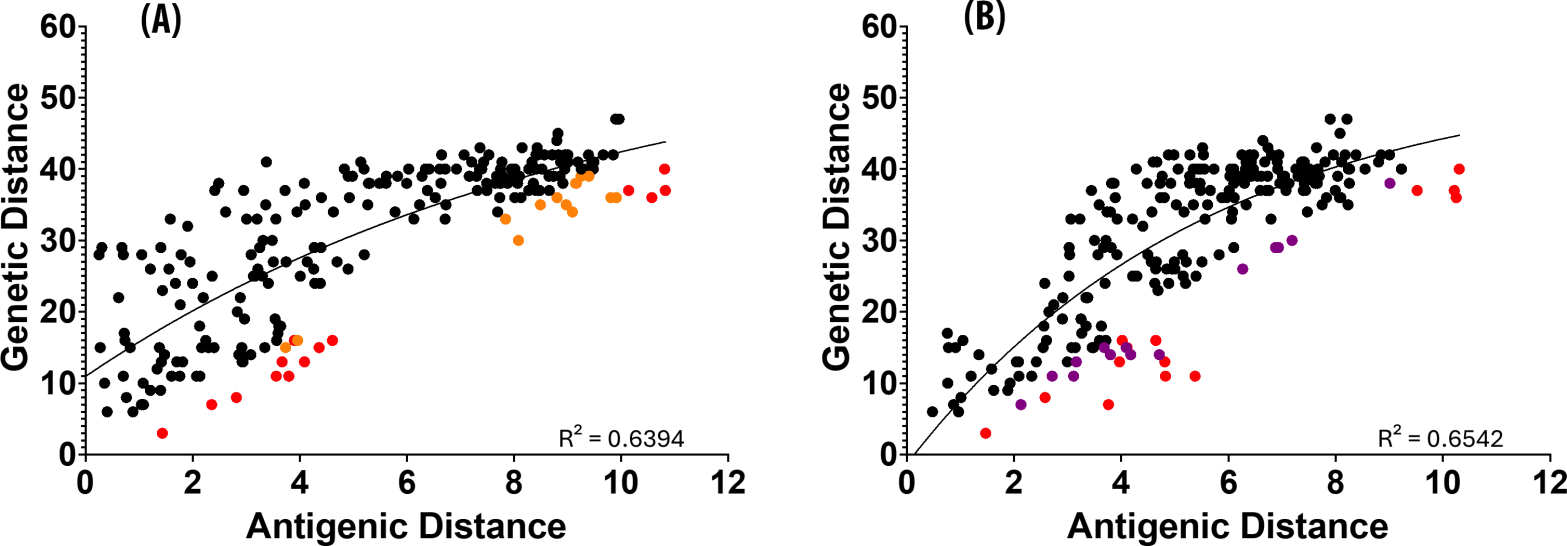
Scatterplots comparing HA1 genetic versus antigenic distances with non-linear association curves. Points with highest antigenic-to-genetic distance ratios are highlighted, showing similarities and differences between (**A**) 2D and (**B**) 3D cartography analyses.

Antigenic distances derived from both 2D and 3D antigenic cartography maps were plotted against the corresponding amino acid variances in the highly variable HA1 region (Fig. 5). Instances where large antigenic differences corresponded to small genetic differences suggest that a limited number of residue changes significantly impact antigenicity. To quantify this relationship, the ratio of antigenic distance to genetic distance was calculated for each antigen pair (Table S3). The pairs with the highest ratios (*n* = 28) were highlighted on the scatter plots, with shared pairs between 2D and 3D maps colored in red, and pairs unique to the 2D or 3D maps colored orange and purple, respectively.

Both plots shared 14 out of 28 (50%) viral pairings, with the remaining 14 pairings unique to each plot. This difference in pair matching reflects the influence of the z-axis in the 3D plot on the overall distances between points. To determine which plot offered the most robust representation of the data, a non-linear, one-phase association curve was fitted to each, with the coefficient of determination (*R*²) reported. The 2D and 3D models yielded *R*² values of 0.6394 and 0.6542, respectively.

Residue variances in the HA1 region between the virus pairs were identified and highlighted (Table 4). Given its central position on the antigenic map, RUS18 was selected as the template HA for site-directed mutagenesis. Putative residues were mutated from the original amino acids in RUS18 to the alternatives predicted from paired comparisons. Additionally, the prevalence of each residue variant within the study strains was highlighted.

**TABLE 4:** Putative antigenic residue selection and abundance in H5Nx study strains^*a*^^,^^*b*^

Strain name	Residue position																							
	35	40	43	47	54	66	71	82	83	94	97	114	115	118	119	120	123	124	126*	127	129	133	136	138
RUS18	K	K	D	V	N	M	I	R	A	S	N	I	L	P	K	S	P	N	E	T	L	A	P	Q
VMN12a	.	.	.	I	D	.	.	K	.	V	D	.	Q	.	.	.	.	.	.	A	.	.	.	.
VMN12b	.	.	.	.	D	.	.	K	.	V	D	.	Q	.	.	.	.	S	.	A	.	.	.	.
IDN10a	R	.	.	.	D	.	.	K	.	.	D	.	Q	.	.	.	S	D	.	A	S	S	.	L
IDN10b	.	.	N	.	D	.	.	K	.	.	D	.	Q	H	.	.	S	D	.	A	S	S	.	L
EGY15	.	.	N	.	D	.	L	K	I	N	D	.	Q	.	.	D	S	D	.	A	del	S	.	.
EGY10	.	.	N	.	D	.	L	K	I	N	D	.	Q	.	.	N	S	D	.	A	del	S	.	.
EGY13	.	.	.	.	D	.	L	K	I	N	D	.	Q	.	.	D	S	D	.	V	del	.	.	.
BGD11	.	.	.	.	D	.	L	K	I	.	D	.	Q	.	.	.	S	D	.	A	S	S	.	.
NPL14	.	.	.	.	.	.	.	K	.	N	D	.	R	.	.	D	S	D	.	A	.	.	.	.
CHN13	.	.	.	.	.	.	T	K	T	.	D	.	R	.	Q	D	.	D	.	A	.	.	S	.
VMN12c	.	.	.	.	.	L	.	K	.	N	D	.	Q	.	.	D	S	D	.	A	.	.	S	.
CHN14	.	.	.	.	.	.	.	.	.	N	D	.	.	.	.	.	T	.	.	.	.	.	.	.
IRN17	.	.	.	.	.	.	.	K	.	.	D	.	.	.	.	.	.	.	.	.	.	.	.	L
USA15	.	.	.	.	.	I	.	.	.	T	D	T	.	.	R	.	.	.	.	.	.	.	.	.
CHN15a	.	.	.	.	.	.	.	.	.	N	D	T	.	.	.	.	.	.	del	.	S	.	.	.
CHN16	.	.	.	.	.	.	.	.	.	N	D	T	.	.	.	.	.	.	del	.	P	.	.	L
CHN15b	.	R	.	.	.	.	.	.	.	N	D	T	.	.	.	.	.	.	del	.	S	.	.	.
TWN17	.	R	.	.	.	.	.	.	T	N	D	T	.	.	.	.	.	.	.	.	del	.	.	.
CHN16	.	R	.	.	.	.	.	.	.	N	D	T	.	.	.	.	.	.	.	.	.	.	.	.
VMN20	.	R	.	.	.	.	.	.	.	N	D	T	.	.	.	.	.	.	.	.	.	.	Q	.
CHN21	.	.	.	.	.	.	.	.	.	N	D	T	Q	.	.	E	S	.	T	.	del	.	.	.

	140	151	154	155	156	161	168	169	184	185	195	198	204	207	210	223	229	234	235	275	277	282	297	309
RUS18	T	I	N	D	A	K	N	R	A	E	T	I	T	Q	V	R	F	K	P	N	K	V	L	N
VMN12a	R	.	.	S	T	.	G	Q	.	A	.	.	.	.	T	S	.	.	.	S	.	M	.	.
VMN12b	Q	.	.	S	T	.	.	Q	.	A	.	.	.	.	T	S	.	.	.	.	.	M	.	T
IDN10a	S	.	.	S	T	.	.	Q	E	A	.	.	.	.	.	S	.	.	.	.	.	M	I	.
IDN10b	S	.	.	S	T	.	.	Q	E	A	.	.	.	.	.	S	.	.	.	.	.	M	.	D
EGY15	R	T	.	.	.	.	.	Q	.	A	.	.	.	.	.	S	.	.	S	.	.	I	.	.
EGY10	R	T	D	N	.	.	.	Q	.	T	.	.	.	.	.	S	.	.	S	.	.	I	.	.
EGY13	R	T	.	.	.	.	.	Q	D	A	.	.	.	.	.	S	.	.	S	.	.	I	.	.
BGD11	R	.	.	.	.	.	.	Q	.	A	.	.	.	L	.	S	.	.	.	.	.	.	.	.
NPL14	N	.	D	N	T	.	.	Q	E	A	.	.	.	.	.	S	.	.	.	.	R	I	.	.
CHN13	N	L	.	.	.	T	.	K	E	A	.	V	.	.	.	.	.	.	.	S	R	M	.	.
VMN12c	N	.	D	N	.	.	.	.	E	A	.	.	.	.	.	S	.	.	.	.	R	I	.	.
CHN14	.	.	.	.	.	.	.	Q	.	A	.	.	.	.	.	.	.	.	.	.	.	I	.	.
IRN17	M	.	.	.	.	.	.	.	.	.	.	.	.	.	.	.	.	.	.	.	R	.	.	.
USA15	A	.	.	.	.	.	.	.	.	A	A	V	.	.	.	S	.	.	.	.	.	I	.	.
CHN15a	M	T	.	.	.	.	.	.	.	.	.	V	.	.	.	.	.	R	.	.	.	I	.	.
CHN16	M	T	.	.	S	.	.	.	.	A	.	V	I	.	.	S	.	.	.	.	.	I	F	.
CHN15b	M	T	.	.	.	.	Y	.	.	A	.	V	.	.	E	.	.	.	.	.	.	I	.	.
TWN17	V	T	.	.	.	.	.	G	E	A	.	V	.	.	.	Q	.	.	.	.	.	I	.	.
CHN16	M	T	.	.	.	.	.	.	E	A	.	V	.	.	.	.	.	.	S	.	.	I	.	.
VMN20	M	.	.	.	.	E	.	S	.	A	.	V	.	.	.	.	Y	.	.	.	.	I	.	.
CHN21	V	T	.	.	.	.	.	K	V	.	.	V	.	.	.	.	.	R	.	.	.	I	.	.

^*a*^ Mutations highlighted in grayscale, with differences shown and identical residues (.) in reference to A/Chicken/Cheboksary/854/2018 (RUS18). Other variances within the putative position are unhighlighted. Each position is colored according to antigenic site location (**Site A**: 114–142; **Site B**: 151–156, 182–190, 192–195; **Site C**: 39–40, 43–44, 267–273, 275; **Site D**: 163, 197–204, 212–216, 218–223, 238; **Site E**: 53–54, 66, 68–74, 82–85).

^*b*^ del = residue deletion; * – the addition of an N-linked glycan by forming the glycan sequon following deletion; underlined – the removal of a predicted N-linked glycan.

**Fig 6:**
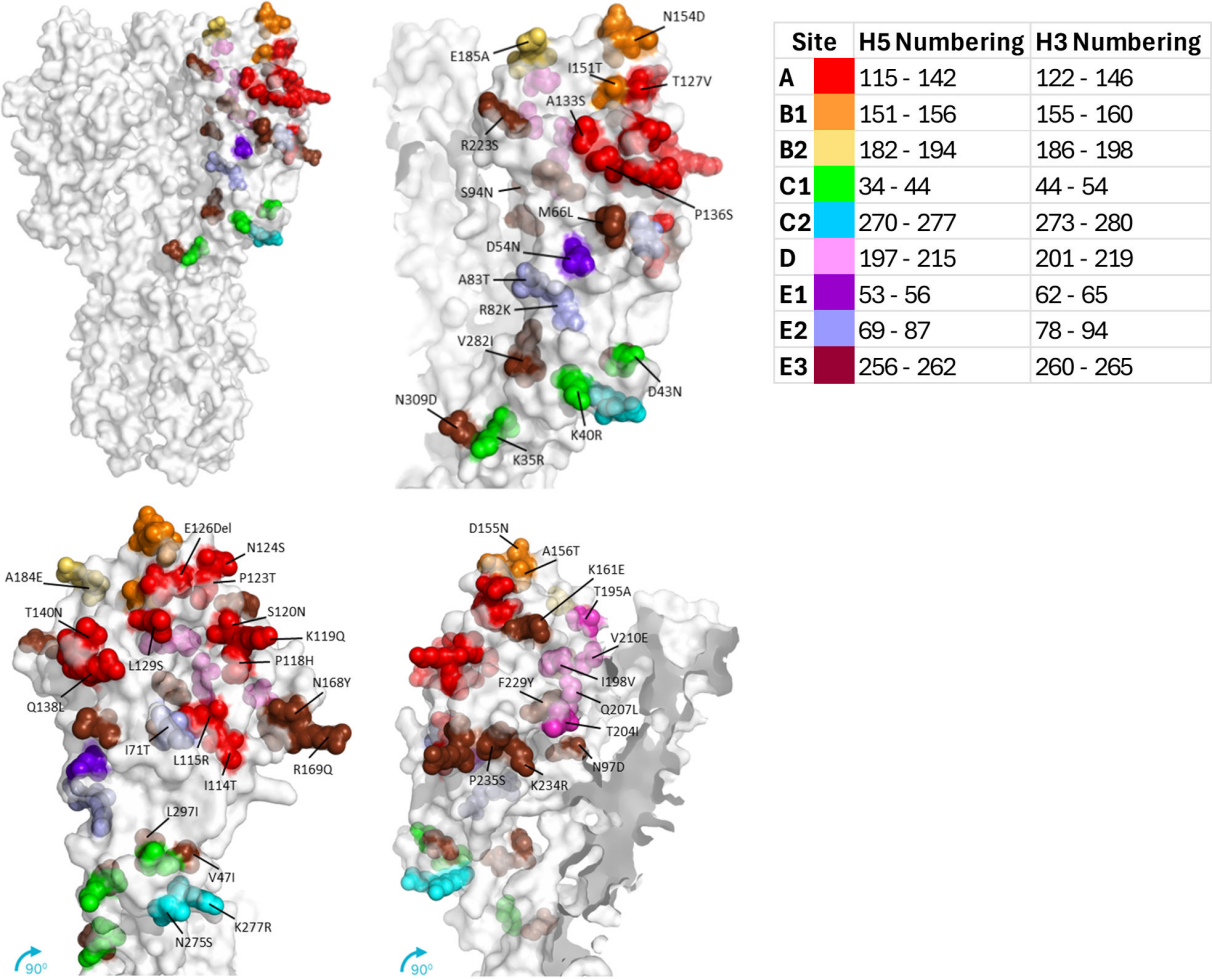
Protein model of the putative residue locations on A/Chicken/Cheboksary/854/2018 HA. Putative residues are displayed as spheres on the model surface, colored by estimated antigenic site location (21, 27), or colored brown if no corresponding site applies. Models are turned 90° clockwise and labeled for best visualization. Models constructed and edited in Swiss-Model (28, 29) (using PDB: 6pcx.1 as template) and PyMOL (30). Mature numbering used for site location, with H5 numbering for putative residues.

From the 28 pairwise comparisons, 48 amino acid variations within the HA1 region were identified. Of these, 21 out of 48 (43.8%) variations were unique to a single strain, while the remaining variations were observed across multiple strains in this study (Fig. 6). Among these residues, four corresponded to predicted alterations in N-linked glycosylation sites, including one instance where an amino acid deletion introduced a new N-linked sequon. Subsequently, the putative residues were mapped onto a monomeric HA protein model to evaluate their location relative to known antigenic sites and overall structural position. Residues buried within the HA structure are less likely to result from immune escape and were therefore excluded from further consideration. Each putative antigenic epitope was then highlighted and color-coded based on its predicted antigenic site using the H5 HA model (PDB ID: 6pcx.1) (27) (Fig. 6).

The antigenic sites of H5 are spread within the HA1 of the HA. Sites A, B, and D are located within the receptor-binding site (RBS), with A and B surrounding the receptor-binding pocket. Sites E and C2 are distal to the RBS, within the less variable vestigial esterase domain at the base of the globular head. Site C1 is the most distal region, forming a concentrated region at the top of the stem, and is the first site within the open-reading frame (ORF). All putative epitopes were on the surface of the HA or protruded from the protein, including those outside of a previously determined antigenic region (27), and so were taken forward for further investigation.

### Antigenic characterization of putative antigenic epitopes

**Fig 7:**
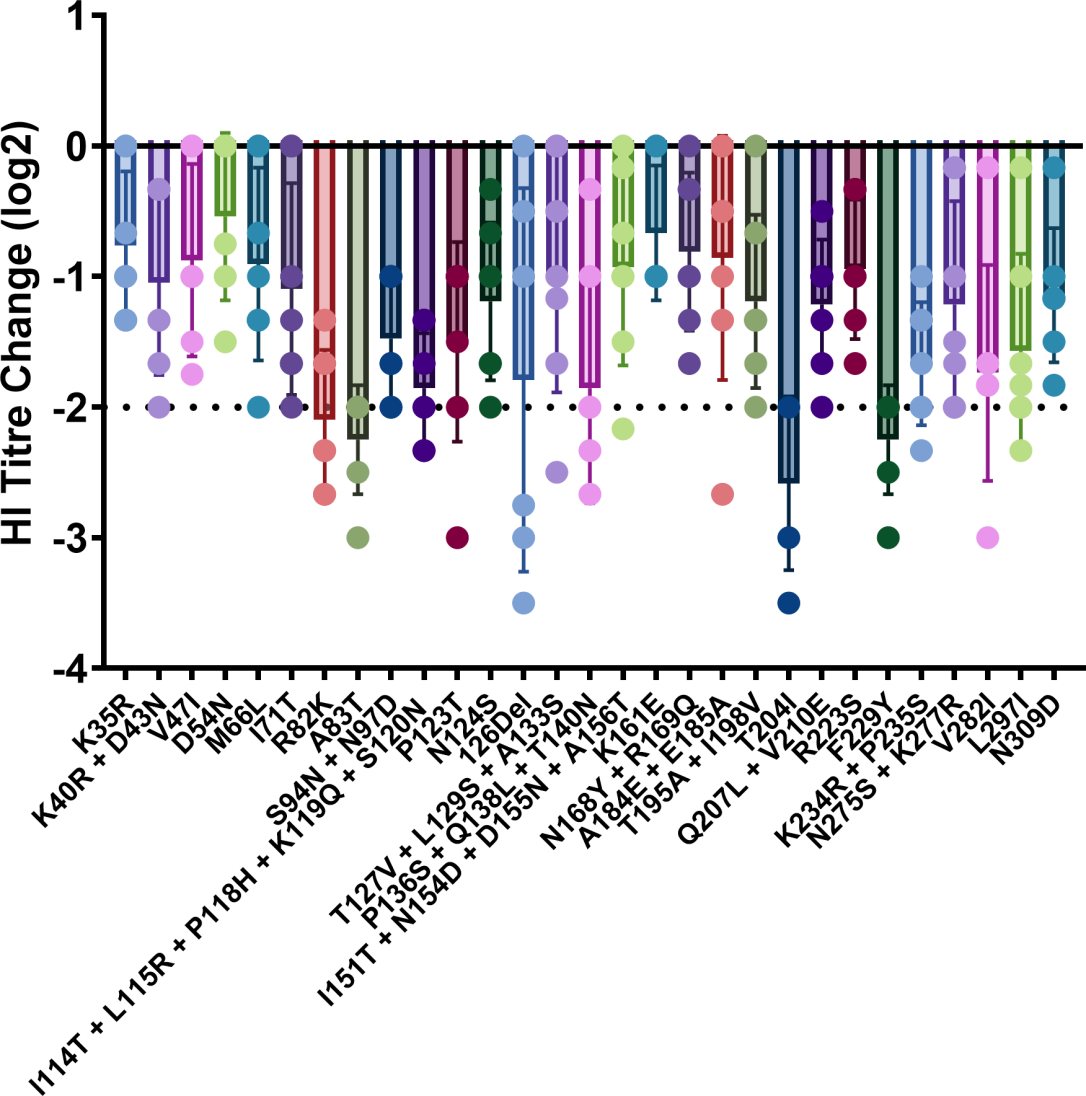
GMT change by HI of putative antigenic residues to previously homologous whole virus antisera. Titer changes greater than 2 log_2_ are considered a significant antigenic change (31). The residues are indicated by the original amino acid, the position, and the amino acid change. Plots are individual White Leghorn chicken sera and bars the range of standard deviation. Each plot GMT is the combination of three technical repeats. Mean, standard deviation, and percentage coefficient of variation displayed in Table S5.

Putative antigenic residues were introduced into the template HA, RUS18, via site-directed mutagenesis, with residues in close proximity mutated simultaneously. The 48 individual residue changes were consolidated into 29 mutagenesis reactions. Following confirmation by Sanger sequencing, the mutated HAs were rescued as recombinant viruses and evaluated for their impact on antigenicity using the HI assay with sera raised against the wild-type RUS18 HA. The changes in antigenic titers of the homologous antisera against each mutant virus were plotted as averages from three technical repeats and individual biological repeats (*n* = 7) (Fig. 7). A change in HI titer of ≥ 2log_2_ (equivalent to a 4 HAU difference) was used as the threshold to classify an antigenic variant (31).

Each putative residue mutation or combination influenced antigenicity to varying degrees, with three mutations, A83T, T204I, and F229Y, exceeding the antigenic variant threshold, showing GMT changes greater than 2log_2_. Among the mutants, 18 (62.1%) exhibited individual sera titer changes of 2 log_2_ or more, although their GMTs did not surpass the threshold. Notably, mutations A83T, 126del, T204I, F229Y, and V282I induced sera titer changes equal to or greater than 3 log_2_. The data also revealed substantial individual variation in neutralizing antibody responses, with some mutant titers differing by up to 2.5 log_2_ between birds vaccinated with the same antigen, suggesting considerable heterogeneity in immune response despite identical antigen exposure and vaccination conditions.

## DISCUSSION

The control of H5Nx AIVs in endemic regions relies heavily on vaccination strategies aimed at reducing poultry morbidity and mortality and limiting viral transmission. However, the recent unprecedented global spread of H5Nx AIVs, particularly those belonging to clade 2.3.4.4b, has heightened the demand for vaccine interventions not only in endemic regions but also for the protection of poultry and vulnerable wild bird populations worldwide. Despite the dominance of clade 2.3.4.4b, co-circulation with other antigenically distinct lineages, including endemic clades 2.3.2.1a, 2.3.2.1c, and other subclades of 2.3.4.4, continues to pose a significant challenge (32, 33). The success of vaccination campaigns is hindered by both the antigenic diversity among circulating strains and mismatches between vaccine seed strains and field viruses. Therefore, this study aimed to identify specific amino acid residues that contribute to antigenic differences between recently or currently circulating H5Nx clades, which may compromise vaccine efficacy and inform vaccine seed strain selection. While genetic evolution is typically characterized through phylogenetic analysis (34), antigenic evolution can be more directly assessed using antigenic cartography derived from HI assay data (35). Accordingly, both phylogenetic and antigenic mapping approaches were employed in tandem to identify and evaluate putative residues driving antigenic diversity.

Clade-representative viruses were selected through phylogenetic analysis. Notably, clade 2.3.4.4 was greatly distinguished with substantial inter- and intra-clade genetic variability, with up to 28 amino acid intra-clade differences observed. de Vries et al. (36) previously identified seven characteristic mutations within the HA protein of clade 2.3.4.4 (K82R, T156A, N183D, K218Q, S223R, N240H, and A263T). All but one (S223R) were consistently conserved across 2.3.4.4 subclades; S223R appeared to have been lost in subclades 2.3.4.4c to 2.3.4.4h. Interestingly, A263T, although conserved within clade 2.3.4.4, also appeared across several other clades (1 to 2.2.2.1), with notable exceptions in clades 2.1.3.2, 2.3.2.1a–c, and 2.4. Across the data set, residue 263 exhibited a roughly 50:50 distribution of alanine and threonine. Furthermore, 12 additional residues were conserved within HA1 across all 2.3.4.4 viruses, with two more residues conserved specifically in subclades 2.3.4.4c–h. Collectively, clade 2.3.4.4 exhibited the most conserved antigenic markers, followed by clade 2.3.2.1, another genetically and antigenically distinct clade frequently implicated in recent poultry outbreaks (9, 10). These findings highlight the profound antigenic and genetic divergence of clade 2.3.4.4 and the resulting complexity in formulating a broadly protective H5 vaccine.

Another immune escape mechanism employed by influenza A viruses involves the acquisition of N-linked glycosylation sites near antigenic regions. The addition of bulky oligosaccharide moieties can sterically hinder antibody access to epitopes. Seven N-linked glycosylation sites were conserved across all strains, with three (302, 500, and 559) located within the stem domain of HA. These stem glycans are critical for maintaining the structural integrity and fusion functionality of HA (37). In contrast, glycosylation sites within the globular head are highly variable and contribute to immune evasion (38) and modulation of receptor binding (39). Frequently occurring glycosylation sites at positions 26, 27, 39, 100, 170, 181, and 209 have been previously reported across multiple H5 clades and are thought to stabilize the protein or shield antigenic epitopes (40, 41). Less common glycosylation sites at positions 70, 140, 182, and 289 were observed sporadically, likely reflecting strain-specific adaptations. These variable glycans, often located within or near antigenic sites in the globular head region of HA, may arise in response to immune pressure or serve to alter receptor binding affinity. Some GMTs presented greater cross-reactive titers from heterologous antisera compared to homologous. This discrepancy was largely seen with USA15 and may be attributed to the loss of a conserved N-linked glycan at site 209. The other circumstance appeared with VMN12b and VMN12c; due to the genetic variation within the sequence, the frequently conserved sequon 170 fell below the predicted threshold, which therefore may suggest a lack of N-linked glycan and thus exposure of critical epitopes.

Importantly, genetic diversity does not always correlate with antigenic diversity. Even minor amino acid substitutions in HA can lead to substantial antigenic shifts. Vaccinated chickens produced titers well above protective thresholds to homologous antigen–sera pairs, confirming successful inoculation and seroconversion. While there was a general correlation between genetic and antigenic variation, clade 2.3.4.4 again demonstrated marked antigenic heterogeneity. Consistent with previous findings, clade 2.3.4.4 viruses were poorly neutralized by monoclonal or polyclonal antibodies raised against other clades, including those co-circulating within the same geographical regions (42). The rapid and ongoing diversification of 2.3.4.4 subclades has been widely documented, most notably, the persistence of clade 2.3.4.4b in wild bird populations across Europe, the Americas, and Africa, and the endemic circulation of clades 2.3.4.4d-h in Asian poultry (43). This independent evolution within each subclade is driving further antigenic divergence. Consequently, the subclades are becoming antigenically distinct entities, potentially warranting a reassessment of the current H5 clade nomenclature to better align with phylogenetic and antigenic boundaries (44).

Antigenic cartography enables the visualization of antigenic evolution and the relationships between viral strains and corresponding antisera in two- and three-dimensional space. A key observation from both maps was the clear segregation of clade 2.3.4.4a–h antigens from all other clades (1.1 to 2.3.2.1b). In line with the genetic analysis, clade 2.3.4.4 exhibited marked antigenic uniqueness, demonstrating minimal cross-reactivity with other clades and confirming its pronounced antigenic divergence. Furthermore, the dispersion of antigen plots within clade 2.3.4.4 revealed considerable intra-clade antigenic variation. These findings align with multiple recent studies reporting the pronounced antigenic distinctiveness of clade 2.3.4.4, including the currently dominant subclade 2.3.4.4b, which exhibits notable divergence even within its own lineage (43, 45, 46).

The virus pairs exhibiting the highest antigenic-to-genetic distance ratios, indicating substantial antigenic divergence with minimal amino acid variation, were selected for further analysis. Amino acid differences between these virus pairs were identified, yielding a list of putative residues for downstream analysis. Notably, the highest antigenic/genetic ratios were observed between viruses from closely related clades (e.g., 2.2.1 and 2.2.1.2, or 2.3.4.4d and 2.3.4.4e). Several amino acid substitutions were recurrent across multiple strains, particularly among those belonging to similar clades or subject to shared geographical constraints. As anticipated, the majority of variations were concentrated in the globular head domain of HA1 (amino acids 42–272), a region commonly implicated in antibody recognition.

To differentiate putative antigenic epitopes from synonymous or structurally buried mutations, each candidate residue was mapped onto the HA monomer model of RUS18. Mutations not exposed on the surface of the protein were excluded, as their likelihood of contributing to antibody escape is low. Surface-exposed residues were further analyzed in the context of defined antigenic regions. For H5 HAs, five principal antigenic sites (A–E) have been described based on their spatial distribution across the HA head. While consideration of these canonical regions helps reduce false positives (47), this study also aimed to identify novel epitopes located outside these predefined sites. This approach is supported by reports of escape mutations arising beyond the established antigenic regions, often near the receptor-binding site or adjacent to residues known to critically influence receptor affinity factors that may confound HI assay interpretations (48, 49).

A total of 48 putative antigenic residues were identified, of which 34 have been previously reported as antigenic epitopes (48–60) while 16 were either novel or located outside established antigenic sites. Of the 32 residues situated within defined antigenic regions, their distribution was as follows: Site A (*n* = 14), Site B (*n* = 7), Site C (*n* = 3), Site D (*n* = 3), and Site E (*n* = 5). The most influential epitopes were predominantly located in or near the highly variable and well-characterized RBD of HA. Site A includes the critical 130-loop (H5 numbering: residues 131–134), while Site B encompasses both the 150-loop (residues 151–159) and the 190-helix (residues 186–194), all structural motifs essential for receptor affinity, specificity, and host range (61, 62). Site D contributes the final RBD element, the 220-loop (residues 217–224), which also influences receptor specificity, although to a lesser extent than Sites A and B. In contrast, Sites C and E do not overlap with the structural components of the RBD but instead coincide with the vestigial esterase and fusion domains of HA. As a result, antibodies targeting the RBD often exert potent neutralizing activity and tend to be strain- or clade-specific, contributing to rapid immune-driven selection at these sites. Conversely, antibodies directed against the vestigial esterase domain are typically non-neutralizing but may confer cross-clade protection by engaging Fc-mediated effector functions such as antibody-dependent cellular phagocytosis (ADCP) and antibody-dependent cellular cytotoxicity (ADCC) (63–67).

Each putative residue was introduced into a candidate HA (RUS18) for investigation of antigenic influence using previously homologous antisera. RUS18 was chosen due to its central positioning within the antigenic cartography maps, low saturation of N-linked glycans, as some were to be removed and introduced, which may distort intracellular trafficking, and relevance to currently dominating strains. Of the 29 mutants, 18 (62.1%) achieved individual values over or equal to a 2 log_2_ titer change but did not surpass the threshold by GMT. However, A83T, 126del, T204I, F229Y, and V282I each induced sera titer above or equal to a 3 log_2_ change. These mutations were within 2.3.2.1b, 2.3.4.4d, 2.3.4.4d, 2.3.4.4g, and both 2.2.1 and 2.3.4.4a-h, except for 2.3.4.4b, respectively. A83T, 126del, and T204I mutations are situated within antigenic sites E, A, and D, respectively, with F229T and V282 situated outside of an assigned site.

Three residues A83T, 126del, and V282I are recognized antigenic epitopes. Residue 83 was identified through peptide mutagenesis studies, in which its substitution resulted in the loss of neutralization by monoclonal antibodies, confirming its antigenic relevance (53). This study supports and complements those earlier findings. Site 83 has become more variable since 2024 and has presented V, S, N, and D, with very few isolates of T in the USA strictly limited to avian isolates (GISAID). Site 126 has been reported to acquire N-linked glycosylation, effectively masking an epitope targeted by monoclonal antibodies. This modification not only reduces virulence in mice, potentially due to increased susceptibility to serum inhibitors, but has also emerged as an antigenic escape mutation (57, 68). In agreement with Kaverin et al. (68), the loss of glycosylation at this site in the present study was associated with reduced cross-reactivity to antisera and significantly decreased HI titers. Similarly, V282I has been previously implicated in antigenic drift and identified as a monoclonal antibody escape mutation (50). Again, V282I has been absent until 2025 but has been repeatedly isolated in the United States (genotype D1.3 [genotypes apply to clade 2.3.4.4b henceforth]), across Europe (genotype EA-2022-BB), and Bangladesh (GISAID) from domestic and wild avians. Moreover, V282I has been sporadically isolated from cattle milk in the USA (genotype B3.13). Interestingly, both A83T and V282I have emerged in a chicken isolate in the United States in 2025 (genotype unknown).

In contrast, T204I and F229Y have not been directly characterized as epitope-defining mutations. However, residue 204 resides within the highly antigenic site D, proximal to the RBD, and is generally conserved as T across clades. Although residue 229 does not fall within any defined antigenic site, it is structurally adjacent to residue 204 (Fig. 6), suggesting it may exert an indirect influence on local antigenicity through conformational effects or epitope shielding. F229Y has been absent from strains until 2025, where it has emerged in chickens in the United States (GISAID) within a genotype D1.1 isolate. Similarly, T204I has also been absent until a very recent genotype D1.2.1 isolate from a Mute Swan in England in late 2025 (GISAID).

### Conclusion

The dominant and currently circulating clade 2.3.4.4 is both genetically and antigenically distinct from predecessor clades, exhibiting substantial intra-clade variability. Through detailed mapping of the highly variable HA1 region, a combination of antigenic and non-antigenic residues was identified that differentiates clades. Of the mutations assessed, 18 (62.1%) induced a ≥2 log_2_ change in HI titer individually but did not exceed the threshold when evaluated by GMT. In contrast, five mutations A83T, 126del, T204I, F229Y, and V282I each resulted in ≥3 log_2_ reductions in HI titer, highlighting their potential as key antigenic determinants. More importantly, A83T, T204I, F229Y, and V282I have sporadically emerged in 2025 in avian clade 2.3.4.4b isolates independently or in combination, and V282I has additionally been isolated in American cattle milk. These may pose as emerging variants, and our early identification may significantly aid antigenic mapping of the emerging and evolving epizootic.

In conclusion, this integrative approach, combining genetic and antigenic data, protein modeling exclusion, and comparative analysis, provides a more tailored experimental approach to both identify clade antigenic disparities and enhance our understanding of the antigenic evolution of H5 HA, particularly within subclade 2.3.4.4b. The identification of residues that drive or fail to drive antigenic drift provides valuable insight for refining vaccine design and can be applied to identify potentially emerging variants. These findings support the development of more effective immunogens for poultry and, in light of recent zoonotic spillovers, potentially for use in mammals, including humans.

## MATERIALS AND METHODS

### Acquisition and modification of sequences

Virus strains were selected as representatives of recently or currently active clades of H5Nx Gs/Gd lineages (clade 1-2.3.4.4h). Representative strains from clades 1.1.1 to 2.3.2.1c were selected based on the 2014 updated nomenclature published by the WHO/OIE/FAO (14), while strains from clades 2.3.4.4a and 2.3.4.4d to 2.3.4.4h were selected according to a study by Bui et al. (15) for the CDC. Complete HA sequences of the selected strains were downloaded from the NCBI (National Center for Biotechnology Information) (16) and GISAID (17) databases.

Downloaded HA sequences were inspected for nucleotide errors or frameshifts. Sequences were visualized and aligned using MUSCLE (69), and clustering was performed using UPGMA (70) in MEGA (version 10) (19). Nucleotide sequences were trimmed to the open reading frame (ORF) and translated to ensure correct structural formation. In cases of frameshifting or sequence errors, alignments were reviewed, and the most common nucleotide at the affected position was introduced to correct the sequence.

Untranslated regions (UTRs) complementary to the pHW2000 expression system were added to both the 5′ and 3′ ends of the sequences. These UTRs included gene- and subtype-specific segments along with BsmBI (Esp3I) restriction enzyme sites (71, 72). The translated region was further modified by replacing the polybasic amino acid cleavage site with a monobasic site to remove high virulence, enabling experimentation at containment level 2. The entire sequence was also examined for additional BsmBI recognition sites, which were removed through synonymous codon substitutions. The modified HA sequences were synthesized by GeneArt Gene Synthesis (Thermo Fisher Scientific) and cloned into the pMT vector by the manufacturer.

### Gene subcloning

The pMT vectors containing HA gene inserts and empty pHW2000 vectors were digested at the designated restriction sites within the UTRs using the BsmBI enzyme and CutSmart Buffer (NEB), according to the manufacturer’s instructions. The pHW2000 vector was additionally dephosphorylated prior to ligation using Antarctic Phosphatase (NEB). HA inserts were isolated by gel electrophoresis and extracted using the QIAquick Gel Extraction Kit (Qiagen). The digested HA genes and digested/dephosphorylated pHW2000 vector were then ligated using the T4 DNA Ligase Kit (NEB), following the manufacturer’s instructions.

### Bacterial transformation and plasmid expansion

Ligation products were transformed into in-house-prepared chemically competent *Escherichia coli* (DH5-α, NEB) using the standard heat-shock protocol (73). For plasmid sequencing by Sanger, plasmids were extracted using the QIAprep Spin Miniprep Kit (Qiagen), following the manufacturer’s instructions. For preparation of working stocks, plasmids were extracted from larger bacterial cultures using the QIAprep Spin Maxiprep Kit (Qiagen), also according to the manufacturer’s instructions.

### Generation of recombinant influenza viruses

The well-established eight-plasmid bidirectional reverse genetics (RG) system was used to generate recombinant H5Nx viruses, incorporating the HA and NA genes from H5Nx strains of interest and the internal genes from the lab-adapted H1N1 strain A/Puerto Rico/8/1934 (PR8) (74–77). Virus stocks were expanded by *in ovo* propagation using 10-day-old embryonated White Leghorn chicken eggs (VALO). Eggs were candled daily to monitor embryo viability. Propagation was terminated by chilling at 4°C for a minimum of four hours. Following successful viral propagation, viral RNA (vRNA) was extracted to confirm the HA and NA sequences. Extraction was performed from allantoic fluid using the QIAamp Viral RNA Kit (Qiagen) and the centrifugation protocol, following the manufacturer’s instructions.

### Hemagglutination assay and HI assay

Standard influenza virus hemagglutination assays were performed to titrate virus stocks and assess viral activity based on hemagglutinin binding. These assays are applicable to both active and inactivated influenza viruses (78, 79). Briefly, HI assays were carried out using four HAU of each virus, incubated with twofold serial dilutions of antiserum raised in specific-pathogen-free (SPF) White Leghorn chickens. HI titers were expressed as the reciprocal of the highest antiserum dilution at which hemagglutination was completely inhibited.

### Raising antisera

Stocks of recombinant viruses were prepared in embryonated chicken eggs and subsequently inactivated using 0.1% (vol/vol) β-propiolactone (BPL) (Sigma-Aldrich). To confirm successful inactivation, 200 µL of each virus preparation in allantoic fluid was inoculated into embryonated eggs across three consecutive passages, with each subsequent egg inoculated using allantoic fluid from the previous passage. Virus replication was assessed after each passage by hemagglutination assay and recorded as HAUs. Any virus preparations testing positive for hemagglutination activity required repeated inactivation and passaging.

To achieve the desired viral titers of 1024 HAU/dose of vaccine, the allantoic fluid was concentrated via ultracentrifugation. Vaccines were formulated at a 30:70 antigen-to-adjuvant ratio using MONTANIDE ISA 71 VG adjuvant (SEPPIC), following the manufacturer’s instructions.

Day-old White Leghorn chickens were inoculated subcutaneously at the back of the neck with a single 200 µL vaccine dose and similarly boosted at 21 days post-vaccination. Birds were bled according to their respective weights at 7, 14, 21, 28, 35, and 42 days post-vaccination, with final bleed at day 42 marking the end of the experiment.

### TCID_50_ and microneutralization assay

The amount of live virus in the allantoic fluid was estimated using TCID₅₀ (Tissue Culture Infectious Dose 50%) assays for subsequent use in microneutralization (MN) assays (80). Confluent monolayers of Madin-Darby Canine Kidney (MDCK) cells, seeded into 96-well plates, were washed with PBS and infected with twofold serially diluted virus preparations in quadruplicate. After a one-hour incubation at 37°C, the virus inoculum was removed and replaced with DMEM supplemented with TPCK-trypsin (Sigma-Aldrich). Plates were incubated for 72 hours, after which the cells were stained with crystal violet solution. Viral titers were calculated using the Spearman–Karber method (81).

MN assays were performed using 150 TCID₅₀ of each virus per well. MDCK cells were seeded as described above. Chicken antisera were heat-inactivated at 56°C for 30 minutes, then twofold serially diluted in DMEM. The diluted antisera were incubated with virus in triplicate wells of a separate 96-well plate for one hour at 37°C. Prepared MDCK cells were washed with PBS, and the virus-antisera mixtures were added to the wells and incubated for a further 72 hours at 37°C. Cells were then fixed and stained with crystal violet solution to assess the neutralizing activity of anti-influenza antibodies present in the antisera.

### Site-directed mutagenesis

Putative antigenic epitopes were introduced into the H5 HA of A/chicken/Cheboksary/854/2018 (RUS18) (used a backbone template) by site mutagenesis, using the QuikChange Lightning Site-directed Mutagenesis kit (Agilent), following the manufacturer’s instructions. In all, 18 overlapping primer pairs were designed using the Agilent Primer Design (Agilent) for optimal primer design (Table S4).

### Phylogenetic analysis

Phylogenetic analysis of trimmed amino acid sequences was performed using IQTree (29, 82). Trees were initially constructed as neighbor-joining to predict the most accurate tree model for maximum-likelihood (18). The classification and assignment of clades of H5Nx viruses assume a common ancestral node and monophyletic evolution with a bootstrap value ≥60 at the defining node following 1,000 bootstrap replicates (14). Trees were annotated and colored using FigTree (83).

### Antigenic cartography

Antigenic cartography was used to visualize antigenic relationships and cross-reactivity among a panel of influenza virus antigens and corresponding antisera, based on HI assay titers (35). The maps employ the theoretical framework of “shape space” (84) in which antigenic distances reflect differences in antibody recognition, with shorter distances indicating higher antigenic similarity, often due to structural similarity or shared epitopes. Antigenic maps were constructed and analyzed using Acmacs (26) and RStudio, incorporating the Racmacs package and associated tools. A modified multidimensional scaling (MDS) algorithm was applied to transform HI titers into Euclidean distances, minimizing error to best fit the observed serological data.

### Development of putative antigenic epitopes

To identify key antigenic epitopes that may drive changes in virus antigenicity and impact vaccine efficacy, antigenic distances derived from antigenic cartography were compared pairwise with the corresponding genetic distances, measured as amino acid residue differences, following the approaches described in (35, 85). Virus pairs exhibiting the greatest antigenic distance but the smallest genetic distance, thus yielding the highest antigenic/genetic ratio, were prioritized for further analysis. Amino acid differences between these virus pairs were considered putative antigenic determinants and were subsequently introduced into a template HA sequence via site-directed mutagenesis for functional investigation.

### Statistical analysis

Geometric mean titers were calculated on log_2_-transformed HI titers per virus group (*n* = 22) of antisera (*n* = 4–6). Mean logarithmic titers, standard error, and Student’s t-distribution were generated to calculate linear GMT and confidence intervals of each biological group to validate the individual variation of antisera raised in white Leghorn chickens. Wilcoxon test statistics were applied to non-zero paired differences of log_2_-transformed HI titers to calculate two-paired *P*-values of heterologous and homologous antisera comparison per group. These values were calculated in Microsoft Excel.

All other statistical analyses were carried out using GraphPad Prism 10 software. Non-linear regression models were fitted to the pairwise comparisons of genetic and antigenic distances. The model with the highest *R*-squared value, indicating the greatest proportion of explained variance between the variables, was selected. Pearson’s correlation was fitted to compare HI and MN titers.

## Data Availability

All sequences utilized within this study are available through public databases (GISAID - gisaid.org and Home - Nucleotide - NCBI) under accession numbers shown in Table 1. HI data achieved in this study have been published as geometric mean titers; raw experimental data sets are available upon request. Additional materials (e.g., plasmids or viral isolates) are also available from the corresponding author upon reasonable request and in accordance with institutional and biosafety regulations.

## SUPPLEMENTAL MATERIAL

Figure S1 jvi.00095-26-s0001.tif Comparison of GMT of hemagglutination inhibition and microneutralization tests between NPL14, EGY10, RUS18, USA15, and CHN15b antigens and antisera.

Supplemental legends jvi.00095-26-s0002.docx Descriptive legends for supplemental material.

Table S1 jvi.00095-26-s0003.docx N-linked glycosylation prediction of H5 AIV HA sequences selected for this study.

Table S2 jvi.00095-26-s0004.docx Mean logarithmic HI titers.

Table S3 jvi.00095-26-s0005.docx Pairwise comparisons of antigenic and genetic variances between the study strains, sorted in decreasing values of antigenic distance per genetic distance (ratio).

Table S4 jvi.00095-26-s0006.docx QuikChange Lightning site-directed mutagenesis primer pairs.

Table S5 jvi.00095-26-s0007.docx Mean, standard deviation, and percent coefficient of variation of hemagglutination inhibition of putative antigenic residues.

Table S6 jvi.00095-26-s0008.docx Two-paired p-value of comparison of homologous and heterologous virus antisera.

TABLE S7 jvi.00095-26-s0009.xlsx GISAID Acknowledgement for the contributors of the listed sequences used in this study.

ASM does not own the copyrights to Supplemental Material that may be linked to, or accessed through, an article. The authors have granted ASM a non-exclusive, world-wide license to publish the Supplemental Material files. Please contact the corresponding author directly for reuse.
