# Supplementary figures and images for: Mapping hemagglutinin residues driving antigenic diversity in H5Nx avian influenza viruses
Source: J Virol. 2026 Apr 30;100(6):e00095-26. doi: 10.1128/jvi.00095-26 (PMC13288987; doi:10.1128/jvi.00095-26)

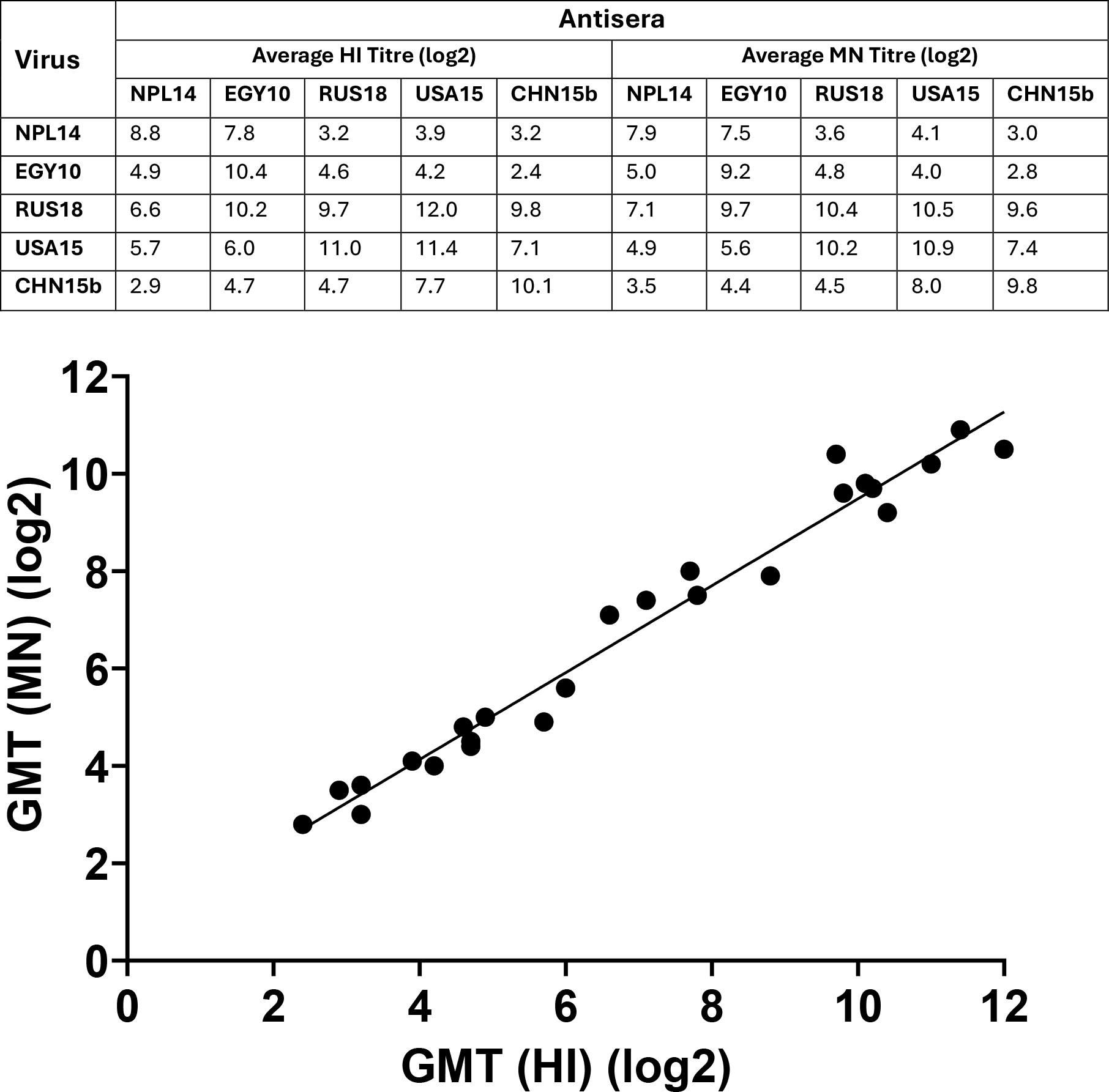

Supplement: Figure S1 — Comparison of GMT of hemagglutination inhibition and microneutralization tests between NPL14, EGY10, RUS18, USA15, and CHN15b antigens and antisera. [file jvi.00095-26-s0001.tif]
