# Supplementary material for: Mapping hemagglutinin residues driving antigenic diversity in H5Nx avian influenza viruses
Source: J Virol. 2026 Apr 30;100(6):e00095-26. doi: 10.1128/jvi.00095-26 (PMC13288987; doi:10.1128/jvi.00095-26)
Supplement: Supplemental legends — Descriptive legends for supplemental material. [file jvi.00095-26-s0002.docx]

Figure S1: Comparison of the geometric mean titre (GMT) of haemagglutination inhibition (HI) and mircroneutralisation (MN) tests between NPL14, EGY10, RUS18, USA15 and CHN15b antigens and antisera, with viruses paired against homologous antisera in bold. Scatterplot of HI against MN titres, with a Pearson correlation R2 value of 0.9712.

Table S1: N-linked glycosylation prediction of H5 AIV HA sequences selected for this study.

Table S2: Mean logarithmic HI titres.

Table S3: Pairwise comparisons of antigenic and genetic variances between the study strains, sorted in decreasing values of antigenic distance per genetic distance (ratio).

Table S4: QuikChange Lightning Site-directed Mutagenesis (Agilent) Primer Pairs.

Table S5: Mean, standard deviation (Std Dev) and percentage coefficient of variation (%CV) of haemagglutination inhibition (HI) of putative antigenic residues

Table S6: Two-paired p-value of comparison of homologous and heterologous virus antisera.
