# Supplementary material for: Mapping hemagglutinin residues driving antigenic diversity in H5Nx avian influenza viruses
Source: J Virol. 2026 Apr 30;100(6):e00095-26. doi: 10.1128/jvi.00095-26 (PMC13288987; doi:10.1128/jvi.00095-26)
Supplement: Table S1 — N-linked glycosylation prediction of H5 AIV HA sequences selected for this study. [file jvi.00095-26-s0003.docx]

**Table S1:** N-linked glycosylation prediction of H5 AIV HA sequences selected for this study. The 'potential' score is the mean output of nine neural networks (Jury Agreement). Values above 0.5 with the sequon Asn-X-Ser/Thr without proline at X are deemed positive predictions. Positions coloured red possess proline at X and are negative. Positions coloured blue are highly conserved/predicted sites.

| **Position** | **VMN12a** | | | | **VMN12b** | | | | **IDN10a** | | | |
| --- | --- | --- | --- | --- | --- | --- | --- | --- | --- | --- | --- | --- |
|  | **Sequon** | **Potential** | **Jury Agreement** | **N-Glyc Result** | **Sequon** | **Potential** | **Jury Agreement** | **N-Glyc Result** | **Sequon** | **Potential** | **Jury Agreement** | **N-Glyc Result** |
| **26** | NNST | 0.3684 | (9/9) | -- | NNST | 0.3684 | (9/9) | -- | NNST | 0.3686 | (9/9) | -- |
| **27** | NSTE | 0.7796 | (9/9) | +++ | NSTE | 0.7796 | (9/9) | +++ | NSTE | 0.7796 | (9/9) | +++ |
| **39** | NVTV | 0.7181 | (9/9) | ++ | NVTV | 0.718 | (9/9) | ++ | NVTV | 0.7181 | (9/9) | ++ |
| **70** |  |  |  |  |  |  |  |  | NCSV | 0.6863 | (9/9) | ++ |
| **100** |  |  |  |  |  |  |  |  | NPTN | 0.523 | (7/9) | + |
| **140** |  |  |  |  |  |  |  |  |  |  |  |  |
| **170** | NSTY | 0.5262 | (8/9) | + | NSTY | 0.5261 | (8/9) | + | NSTY | 0.5299 | (8/9) | + |
| **181** | NNTG | 0.5889 | (6/9) | + | NNTN | 0.6183 | (8/9) | + | NNTN | 0.6139 | (7/9) | + |
| **182** |  |  |  |  |  |  |  |  |  |  |  |  |
| **209** | NPTT | 0.6813 | (8/9) | + | NPTT | 0.6816 | (8/9) | + | NPTT | 0.6836 | (9/9) | ++ |
| **289** | NCST | 0.5356 | (7/9) | + |  |  |  |  |  |  |  |  |
| **302** | NSSM | 0.5996 | (6/9) | + | NSSM | 0.5997 | (6/9) | + | NSSM | 0.5994 | (6/9) | + |
| **500** | NGTY | 0.5824 | (6/9) | + | NGTY | 0.5827 | (6/9) | + | NGTY | 0.6246 | (9/9) | ++ |
| **559** | NGSL | 0.6843 | (9/9) | ++ | NGSL | 0.6843 | (9/9) | ++ | NGSL | 0.6734 | (9/9) | ++ |

| **Position** | **IDN10b** | | | | **EGY15** | | | | **EGY10** | | | |
| --- | --- | --- | --- | --- | --- | --- | --- | --- | --- | --- | --- | --- |
|  | **Sequon** | **Potential** | **Jury Agreement** | **N-Glyc Result** | **Sequon** | **Potential** | **Jury Agreement** | **N-Glyc Result** | **Sequon** | **Potential** | **Jury Agreement** | **N-Glyc Result** |
| **26** | NNST | 0.3686 | (9/9) | -- | NNST | 0.3684 | (9/9) | -- | NNST | 0.3686 | (9/9) | -- |
| **27** | NSTE | 0.7796 | (9/9) | +++ | NSTE | 0.7796 | (9/9) | +++ | NSTE | 0.7796 | (9/9) | +++ |
| **39** | NVTV | 0.7178 | (9/9) | ++ | NVTV | 0.7182 | (9/9) | ++ | NVTV | 0.7181 | (9/9) | ++ |
| **70** |  |  |  |  |  |  |  |  |  |  |  |  |
| **100** | NPTN | 0.523 | (7/9) | + |  |  |  |  |  |  |  |  |
| **140** |  |  |  |  |  |  |  |  |  |  |  |  |
| **170** | NSTY | 0.5293 | (8/9) | + |  |  |  |  |  |  |  |  |
| **181** | NNTN | 0.6652 | (8/9) | + | NNTN | 0.6656 | (8/9) | + | NNTN | 0.6268 | (8/9) | + |
| **182** |  |  |  |  |  |  |  |  |  |  |  |  |
| **209** | NPTT | 0.6836 | (9/9) | ++ | NPTT | 0.6909 | (9/9) | ++ | NPTT | 0.6892 | (9/9) | ++ |
| **289** |  |  |  |  |  |  |  |  |  |  |  |  |
| **302** | NSSM | 0.5993 | (6/9) | + | NSSM | 0.5434 | (6/9) | + | NSSM | 0.5434 | (6/9) | + |
| **500** | NGTY | 0.6261 | (9/9) | ++ | NGTY | 0.5824 | (6/9) | + | NGTY | 0.5824 | (6/9) | + |
| **559** | NGSL | 0.6736 | (9/9) | ++ | NGSL | 0.6841 | (9/9) | ++ | NGSL | 0.681 | (9/9) | ++ |

| **Position** | **EGY13** | | | | **BGD11** | | | | **NPL14** | | | |
| --- | --- | --- | --- | --- | --- | --- | --- | --- | --- | --- | --- | --- |
|  | **Sequon** | **Potential** | **Jury Agreement** | **N-Glyc Result** | **Sequon** | **Potential** | **Jury Agreement** | **N-Glyc Result** | **Sequon** | **Potential** | **Jury Agreement** | **N-Glyc Result** |
| **26** | NNST | 0.3686 | (9/9) | -- | NNST | 0.3682 | (9/9) | -- | NNST | 0.3186 | (9/9) | --- |
| **27** | NSTE | 0.7796 | (9/9) | +++ | NSTE | 0.7794 | (9/9) | +++ | NSTK | 0.8059 | (9/9) | +++ |
| **39** | NVTV | 0.7179 | (9/9) | ++ | NVTV | 0.7179 | (9/9) | ++ | NVTV | 0.719 | (9/9) | ++ |
| **70** |  |  |  |  |  |  |  |  |  |  |  |  |
| **100** |  |  |  |  |  |  |  |  |  |  |  |  |
| **140** |  |  |  |  |  |  |  |  |  |  |  |  |
| **170** |  |  |  |  |  |  |  |  | NSSF | 0.5314 | (6/9) | + |
| **181** | NNTN | 0.6654 | (8/9) | + | NNTS | 0.6832 | (8/9) | + | NNTN | 0.6398 | (8/9) | + |
| **182** |  |  |  |  | NTSQ | 0.5659 | (6/9) | + |  |  |  |  |
| **209** | NPTT | 0.6866 | (9/9) | ++ | NPTT | 0.6813 | (8/9) | + | NPTT | 0.6842 | (9/9) | ++ |
| **289** |  |  |  |  |  |  |  |  |  |  |  |  |
| **302** | NSSM | 0.5433 | (6/9) | + | NSSM | 0.5449 | (6/9) | + | NSSM | 0.542 | (6/9) | + |
| **500** | NGTY | 0.5807 | (6/9) | + | NGTY | 0.5823 | (6/9) | + | NGTY | 0.5741 | (5/9) | + |
| **559** | NGSL | 0.6808 | (9/9) | ++ | NGSL | 0.6841 | (9/9) | ++ | NGSL | 0.6826 | (9/9) | ++ |

| **Position** | **CHN13** | | | | **VMN12c** | | | | **CHN14** | | | |
| --- | --- | --- | --- | --- | --- | --- | --- | --- | --- | --- | --- | --- |
|  | **Sequon** | **Potential** | **Jury Agreement** | **N-Glyc Result** | **Sequon** | **Potential** | **Jury Agreement** | **N-Glyc Result** | **Sequon** | **Potential** | **Jury Agreement** | **N-Glyc Result** |
| **26** | NNST | 0.3708 | (9/9) | -- | NNST | 0.3709 | (9/9) | -- | NNST | 0.3671 | (9/9) | -- |
| **27** | NSTE | 0.7754 | (9/9) | +++ | NSTE | 0.7756 | (9/9) | +++ | NSTE | 0.7796 | (9/9) | +++ |
| **39** | NVTV | 0.718 | (9/9) | ++ | NVTV | 0.7183 | (9/9) | ++ | NVTV | 0.7181 | (9/9) | ++ |
| **70** |  |  |  |  |  |  |  |  |  |  |  |  |
| **100** |  |  |  |  |  |  |  |  |  |  |  |  |
| **140** |  |  |  |  |  |  |  |  |  |  |  |  |
| **170** | NSSF | 0.4609 | (6/9) | - | NSSF | 0.4609 | (6/9) | - |  |  |  |  |
| **181** | NNTN | 0.6288 | (8/9) | + | NNTN | 0.6326 | (8/9) | + | NNTN | 0.641 | (7/9) | + |
| **182** |  |  |  |  |  |  |  |  |  |  |  |  |
| **209** | NPTT | 0.6086 | (8/9) | + | NPTT | 0.6841 | (9/9) | ++ | NPTT | 0.6419 | (8/9) | + |
| **289** | NCST | 0.5202 | (7/9) | + |  |  |  |  |  |  |  |  |
| **302** | NSSM | 0.5987 | (6/9) | + | NSSM | 0.542 | (6/9) | + | NSSM | 0.5433 | (6/9) | + |
| **500** | NGTY | 0.5824 | (6/9) | + | NGTY | 0.58 | (6/9) | + | NGTY | 0.5823 | (6/9) | + |
| **559** | NGSL | 0.6842 | (9/9) | ++ | NGSL | 0.6827 | (9/9) | ++ | NGSL | 0.6841 | (9/9) | ++ |

| **Position** | **RUS18** | | | | **IRN17** | | | | **USA15** | | | |
| --- | --- | --- | --- | --- | --- | --- | --- | --- | --- | --- | --- | --- |
|  | **Sequon** | **Potential** | **Jury Agreement** | **N-Glyc Result** | **Sequon** | **Potential** | **Jury Agreement** | **N-Glyc Result** | **Sequon** | **Potential** | **Jury Agreement** | **N-Glyc Result** |
| **26** | NNST | 0.3684 | (9/9) | -- | NNST | 0.3686 | (9/9) | -- | NNST | 0.317 | (9/9) | --- |
| **27** | NSTE | 0.7796 | (9/9) | +++ | NSTE | 0.7797 | (9/9) | +++ | NSTK | 0.808 | (9/9) | +++ |
| **39** | NVTV | 0.7181 | (9/9) | ++ | NVTV | 0.7181 | (9/9) | ++ | NVTV | 0.7189 | (9/9) | ++ |
| **70** |  |  |  |  |  |  |  |  |  |  |  |  |
| **100** |  |  |  |  |  |  |  |  |  |  |  |  |
| **140** |  |  |  |  |  |  |  |  |  |  |  |  |
| **170** |  |  |  |  |  |  |  |  |  |  |  |  |
| **181** | NNTN | 0.6101 | (7/9) | + | NNTN | 0.6101 | (7/9) | + | NNTN | 0.61 | (7/9) | + |
| **182** |  |  |  |  |  |  |  |  |  |  |  |  |
| **209** | NPTT | 0.6333 | (8/9) | + | NPTT | 0.6332 | (8/9) | + |  |  |  |  |
| **289** |  |  |  |  |  |  |  |  |  |  |  |  |
| **302** | NSSM | 0.5448 | (6/9) | + | NSSM | 0.5434 | (6/9) | + | NSSM | 0.5432 | (6/9) | + |
| **500** | NGTY | 0.5824 | (6/9) | + | NGTY | 0.579 | (6/9) | + | NGTY | 0.5791 | (6/9) | + |
| **559** | NGSL | 0.6853 | (9/9) | ++ | NGSL | 0.6842 | (9/9) | ++ | NGSL | 0.6843 | (9/9) | ++ |

| **Position** | **CHN15a** | | | | **CHN16a** | | | | **CHN15b** | | | |
| --- | --- | --- | --- | --- | --- | --- | --- | --- | --- | --- | --- | --- |
|  | **Sequon** | **Potential** | **Jury Agreement** | **N-Glyc Result** | **Sequon** | **Potential** | **Jury Agreement** | **N-Glyc Result** | **Sequon** | **Potential** | **Jury Agreement** | **N-Glyc Result** |
| **26** | NNST | 0.3684 | (9/9) | -- | NNST | 0.3673 | (9/9) | -- | NNST | 0.3686 | (9/9) | -- |
| **27** | NSTE | 0.7797 | (9/9) | +++ | NSTE | 0.7796 | (9/9) | +++ | NSTE | 0.7796 | (9/9) | +++ |
| **39** | NVTV | 0.7181 | (9/9) | ++ | NVTV | 0.718 | (9/9) | ++ | NVTV | 0.7181 | (9/9) | ++ |
| **70** |  |  |  |  |  |  |  |  |  |  |  |  |
| **100** |  |  |  |  |  |  |  |  |  |  |  |  |
| **140** | NHTS | 0.6477 | (9/9) | ++ | NHTS | 0.6719 | (9/9) | ++ | NHTS | 0.6474 | (9/9) | ++ |
| **170** |  |  |  |  | NDSY | 0.3688 | (9/9) | -- |  |  |  |  |
| **181** | NNTN | 0.5799 | (7/9) | + | NNTN | 0.5673 | (7/9) | + | NNTY | 0.5549 | (6/9) | + |
| **182** |  |  |  |  |  |  |  |  |  |  |  |  |
| **209** | NPTT | 0.6542 | (9/9) | ++ | NPTT | 0.6616 | (9/9) | ++ | NPTT | 0.6614 | (9/9) | ++ |
| **289** |  |  |  |  |  |  |  |  |  |  |  |  |
| **302** | NSSM | 0.5434 | (6/9) | + | NSSM | 0.5436 | (6/9) | + | NSSM | 0.5434 | (6/9) | + |
| **500** | NGTY | 0.5824 | (6/9) | + | NGTY | 0.5823 | (6/9) | + | NGTY | 0.5824 | (6/9) | + |
| **559** | NGSL | 0.6843 | (9/9) | ++ | NGSL | 0.6842 | (9/9) | ++ | NGSL | 0.6844 | (9/9) | ++ |

| **Position** | **TWN17** | | | | **CHN16b** | | | | **VMN20** | | | |
| --- | --- | --- | --- | --- | --- | --- | --- | --- | --- | --- | --- | --- |
|  | **Sequon** | **Potential** | **Jury Agreement** | **N-Glyc Result** | **Sequon** | **Potential** | **Jury Agreement** | **N-Glyc Result** | **Sequon** | **Potential** | **Jury Agreement** | **N-Glyc Result** |
| **26** | NNST | 0.3686 | (9/9) | -- | NNST | 0.3687 | (9/9) | -- | NNST | 0.3718 | (9/9) | -- |
| **27** | NSTE | 0.7797 | (9/9) | +++ | NSTE | 0.7796 | (9/9) | +++ | NSTE | 0.7796 | (9/9) | +++ |
| **39** | NVTV | 0.7182 | (9/9) | ++ | NVTV | 0.718 | (9/9) | ++ | NVTV | 0.7182 | (9/9) | ++ |
| **70** |  |  |  |  |  |  |  |  |  |  |  |  |
| **100** |  |  |  |  |  |  |  |  |  |  |  |  |
| **140** |  |  |  |  |  |  |  |  |  |  |  |  |
| **170** |  |  |  |  |  |  |  |  |  |  |  |  |
| **181** | NNTN | 0.5178 | (6/9) | + | NNTN | 0.5797 | (7/9) | + | NNTN | 0.5867 | (7/9) | + |
| **182** |  |  |  |  |  |  |  |  |  |  |  |  |
| **209** | NPTT | 0.6578 | (9/9) | ++ | NPTT | 0.5947 | (8/9) | + | NPTT | 0.6616 | (9/9) | ++ |
| **289** |  |  |  |  |  |  |  |  |  |  |  |  |
| **302** | NSSM | 0.5436 | (6/9) | + | NSSM | 0.5433 | (6/9) | + | NSSM | 0.5433 | (6/9) | + |
| **500** | NGTY | 0.5827 | (6/9) | + | NGTY | 0.5824 | (6/9) | + | NGTY | 0.5739 | (5/9) | + |
| **559** | NGSL | 0.6847 | (9/9) | ++ | NGSL | 0.6842 | (9/9) | ++ | NGSL | 0.6843 | (9/9) | ++ |

| **Position** | **CHN21** | | | |
| --- | --- | --- | --- | --- |
|  | **Sequon** | **Potential** | **Jury Agreement** | **N-Glyc Result** |
| **26** | NNST | 0.3686 | (9/9) | -- |
| **27** | NSTE | 0.7796 | (9/9) | +++ |
| **39** | NVTV | 0.7181 | (9/9) | ++ |
| **70** | NCSV | 0.6849 | (9/9) | ++ |
| **100** |  |  |  |  |
| **140** | NHTT | 0.5917 | (6/9) | + |
| **170** |  |  |  |  |
| **181** | NNTN | 0.5707 | (7/9) | + |
| **182** |  |  |  |  |
| **209** | NPTT | 0.564 | (7/9) | + |
| **289** |  |  |  |  |
| **302** | NSSM | 0.5518 | (6/9) | + |
| **500** | NGTY | 0.574 | (5/9) | + |
| **559** | NGSL | 0.6843 | (9/9) | ++ |
