# Supplementary material for: Mapping hemagglutinin residues driving antigenic diversity in H5Nx avian influenza viruses
Source: J Virol. 2026 Apr 30;100(6):e00095-26. doi: 10.1128/jvi.00095-26 (PMC13288987; doi:10.1128/jvi.00095-26)
Supplement: Table S2 — Mean logarithmic HI titers. [file jvi.00095-26-s0004.docx]

**Table S2:** Mean logarithmic titres (mean), standard error (SE), Student’s *t* distribution (t-value) and lower and higher confidence intervals (lower/higher) of log_2_ HI titres and geometric mean (GMT) and lower and higher confidence intervals (lower/higher 95% CI) of raw HI titres associated with **Table 3**.

|  | **NPL14** | | | | | | | | **BGD11** | | | | | | | | **IDN10a** | | | | | | | |
| --- | --- | --- | --- | --- | --- | --- | --- | --- | --- | --- | --- | --- | --- | --- | --- | --- | --- | --- | --- | --- | --- | --- | --- | --- |
|  | **Mean** | **SE** | **t-value (95% CI)** | **Lower** | **Higher** | **GMT** | **Lower 95% CI** | **Upper 95% CI** | **Mean** | **SE** | **t-value (95% CI)** | **Lower** | **Higher** | **GMT** | **Lower 95% CI** | **Upper 95% CI** | **Mean** | **SE** | **t-value (95% CI)** | **Lower** | **Higher** | **GMT** | **Lower 95% CI** | **Upper 95% CI** |
| **NPL14** | 8.45 | 0.43 | 2.57 | 7.34 | 9.55 | 348.63 | 162.57 | 747.64 | 6.32 | 0.51 | 2.78 | 4.90 | 7.74 | 79.87 | 29.94 | 213.09 | 6.77 | 0.40 | 2.57 | 5.74 | 7.80 | 108.93 | 53.28 | 222.71 |
| **BGD11** | 4.73 | 0.48 | 2.57 | 3.50 | 5.96 | 26.54 | 11.34 | 62.12 | 9.59 | 0.24 | 2.78 | 8.91 | 10.27 | 769.25 | 480.11 | 1232.53 | 5.87 | 0.39 | 2.57 | 4.87 | 6.87 | 58.61 | 29.32 | 117.16 |
| **IDN10a** | 5.19 | 0.41 | 2.57 | 4.15 | 6.23 | 36.57 | 17.75 | 75.32 | 5.90 | 0.26 | 2.78 | 5.18 | 6.61 | 59.57 | 36.28 | 97.81 | 10.78 | 0.27 | 2.57 | 10.08 | 11.48 | 1760.87 | 1082.13 | 2865.33 |
| **CHN15b** | 2.68 | 0.31 | 2.57 | 1.89 | 3.47 | 6.43 | 3.72 | 11.12 | 3.95 | 0.32 | 2.78 | 3.07 | 4.83 | 15.44 | 8.40 | 28.38 | 5.60 | 0.88 | 2.57 | 3.35 | 7.85 | 48.58 | 10.20 | 231.29 |
| **USA15** | 4.97 | 0.60 | 2.57 | 3.43 | 6.52 | 31.40 | 10.76 | 91.61 | 7.16 | 0.37 | 2.78 | 6.12 | 8.20 | 143.00 | 69.60 | 293.81 | 7.15 | 0.39 | 2.57 | 6.16 | 8.14 | 141.93 | 71.29 | 282.56 |
| **VMN12a** | 3.59 | 0.77 | 2.57 | 1.61 | 5.57 | 12.04 | 3.06 | 47.36 | 6.07 | 0.42 | 2.78 | 4.91 | 7.22 | 66.96 | 29.98 | 149.57 | 5.94 | 0.37 | 2.57 | 5.01 | 6.88 | 61.56 | 32.12 | 117.99 |
| **EGY13** | 4.48 | 0.61 | 2.57 | 2.90 | 6.06 | 22.29 | 7.45 | 66.63 | 4.97 | 0.43 | 2.78 | 3.78 | 6.17 | 31.37 | 13.69 | 71.87 | 4.26 | 0.27 | 2.57 | 3.57 | 4.94 | 19.14 | 11.89 | 30.80 |
| **EGY15** | 5.82 | 0.68 | 2.57 | 4.06 | 7.58 | 56.51 | 16.73 | 190.87 | 7.00 | 0.35 | 2.78 | 6.02 | 7.99 | 128.35 | 64.89 | 253.86 | 5.22 | 0.31 | 2.57 | 4.41 | 6.02 | 37.19 | 21.31 | 64.93 |
| **EGY10** | 4.28 | 0.54 | 2.57 | 2.89 | 5.67 | 19.38 | 7.39 | 50.79 | 6.13 | 0.49 | 2.78 | 4.77 | 7.49 | 69.93 | 27.24 | 179.51 | 5.12 | 0.31 | 2.57 | 4.33 | 5.91 | 34.76 | 20.10 | 60.10 |
| **CHN13** | 6.60 | 0.53 | 2.57 | 5.25 | 7.96 | 97.30 | 37.94 | 249.50 | 2.76 | 0.73 | 2.78 | 0.72 | 4.80 | 6.77 | 1.65 | 27.85 | 4.82 | 0.17 | 2.57 | 4.39 | 5.25 | 28.20 | 20.95 | 37.95 |
| **VMN12b** | 3.45 | 0.72 | 2.57 | 1.59 | 5.31 | 10.92 | 3.02 | 39.54 | 4.13 | 0.37 | 2.78 | 3.09 | 5.17 | 17.50 | 8.52 | 35.95 | 5.20 | 0.35 | 2.57 | 4.30 | 6.11 | 36.87 | 19.67 | 69.13 |
| **IDN10b** | 5.12 | 0.57 | 2.57 | 3.66 | 6.59 | 34.80 | 12.61 | 96.00 | 5.72 | 0.33 | 2.78 | 4.80 | 6.64 | 52.69 | 27.86 | 99.64 | 10.72 | 0.43 | 2.57 | 9.61 | 11.84 | 1690.85 | 781.23 | 3659.54 |
| **VMN12c** | 7.96 | 0.75 | 2.57 | 6.04 | 9.89 | 249.70 | 65.73 | 948.55 | 5.75 | 0.37 | 2.78 | 4.71 | 6.79 | 53.92 | 26.24 | 110.78 | 5.65 | 0.21 | 2.57 | 5.10 | 6.19 | 50.08 | 34.40 | 72.91 |
| **CHN15a** | 4.07 | 0.46 | 2.57 | 2.88 | 5.27 | 16.84 | 7.36 | 38.54 | 4.92 | 0.08 | 2.78 | 4.70 | 5.14 | 30.22 | 25.91 | 35.25 | 6.36 | 0.62 | 2.57 | 4.77 | 7.95 | 82.11 | 27.25 | 247.46 |
| **RUS18** | 5.36 | 1.31 | 2.57 | 2.00 | 8.72 | 41.16 | 4.01 | 422.19 | 8.77 | 0.37 | 2.78 | 7.73 | 9.81 | 436.14 | 212.28 | 896.09 | 10.27 | 0.49 | 2.57 | 9.00 | 11.54 | 1232.18 | 510.61 | 2973.46 |
| **IRN17** | 3.99 | 1.11 | 2.57 | 1.14 | 6.84 | 15.92 | 2.21 | 114.63 | 5.75 | 0.37 | 2.78 | 4.71 | 6.79 | 53.92 | 26.24 | 110.78 | 7.52 | 0.42 | 2.57 | 6.45 | 8.60 | 184.07 | 87.61 | 386.73 |
| **CHN14** | 3.60 | 0.60 | 2.57 | 2.05 | 5.14 | 12.09 | 4.14 | 35.26 | 5.97 | 0.32 | 2.78 | 5.09 | 6.84 | 62.52 | 34.02 | 114.90 | 6.24 | 0.49 | 2.57 | 4.96 | 7.51 | 75.37 | 31.23 | 181.87 |
| **CHN15b** | 3.43 | 0.34 | 2.57 | 2.55 | 4.31 | 10.76 | 5.85 | 19.77 | 4.37 | 0.24 | 2.78 | 3.69 | 5.05 | 20.73 | 12.94 | 33.21 | 6.01 | 0.65 | 2.57 | 4.33 | 7.69 | 64.56 | 20.13 | 207.07 |
| **TWN17** | 2.57 | 0.33 | 2.57 | 1.71 | 3.43 | 5.94 | 3.28 | 10.75 | 4.13 | 0.37 | 2.78 | 3.09 | 5.17 | 17.50 | 8.52 | 35.95 | 5.10 | 0.40 | 2.57 | 4.06 | 6.13 | 34.19 | 16.72 | 69.90 |
| **CHN16** | 2.83 | 0.45 | 2.57 | 1.68 | 3.98 | 7.10 | 3.20 | 15.76 | 4.78 | 0.20 | 2.78 | 4.23 | 5.34 | 27.51 | 18.72 | 40.42 | 5.28 | 0.33 | 2.57 | 4.42 | 6.13 | 38.78 | 21.41 | 70.24 |
| **VMN20** | 2.67 | 0.58 | 2.57 | 1.18 | 4.15 | 6.35 | 2.27 | 17.77 | 4.18 | 0.20 | 2.78 | 3.63 | 4.74 | 18.16 | 12.36 | 26.68 | 5.44 | 0.34 | 2.57 | 4.56 | 6.32 | 43.41 | 23.62 | 79.79 |
| **CHN21** | 3.09 | 0.31 | 2.57 | 2.30 | 3.88 | 8.49 | 4.91 | 14.68 | 4.04 | 0.58 | 2.78 | 2.42 | 5.66 | 16.48 | 5.37 | 50.62 | 4.97 | 0.26 | 2.57 | 4.30 | 5.63 | 31.26 | 19.73 | 49.52 |

|  | **CHN15b** | | | | | | | | **USA15** | | | | | | | | **VMN12a** | | | | | | | |
| --- | --- | --- | --- | --- | --- | --- | --- | --- | --- | --- | --- | --- | --- | --- | --- | --- | --- | --- | --- | --- | --- | --- | --- | --- |
|  | **Mean** | **SE** | **t-value (95% CI)** | **Lower** | **Higher** | **GMT** | **Lower 95% CI** | **Upper 95% CI** | **Mean** | **SE** | **t-value (95% CI)** | **Lower** | **Higher** | **GMT** | **Lower 95% CI** | **Upper 95% CI** | **Mean** | **SE** | **t-value (95% CI)** | **Lower** | **Higher** | **GMT** | **Lower 95% CI** | **Upper 95% CI** |
| **NPL14** | 3.15 | 0.17 | 2.57 | 2.72 | 3.58 | 8.86 | 6.58 | 11.92 | 3.51 | 0.38 | 2.57 | 2.52 | 4.49 | 11.38 | 5.75 | 22.51 | 7.19 | 0.74 | 2.57 | 5.29 | 9.10 | 146.50 | 39.18 | 547.70 |
| **BGD11** | 2.60 | 0.00 | 2.57 | 2.60 | 2.60 | 6.06 | 6.06 | 6.06 | 5.33 | 0.39 | 2.57 | 4.33 | 6.32 | 40.14 | 20.16 | 79.91 | 9.12 | 0.40 | 2.57 | 8.09 | 10.15 | 557.02 | 272.44 | 1138.87 |
| **IDN10a** | 4.34 | 0.56 | 2.57 | 2.90 | 5.79 | 20.31 | 7.45 | 55.35 | 3.93 | 0.67 | 2.57 | 2.22 | 5.64 | 15.25 | 4.65 | 50.01 | 6.32 | 0.67 | 2.57 | 4.59 | 8.04 | 79.69 | 24.12 | 263.34 |
| **CHN15b** | 9.71 | 0.45 | 2.57 | 8.56 | 10.87 | 839.08 | 377.38 | 1865.64 | 4.58 | 0.45 | 2.57 | 3.44 | 5.73 | 23.98 | 10.82 | 53.12 | 2.97 | 0.48 | 2.57 | 1.74 | 4.21 | 7.85 | 3.34 | 18.50 |
| **USA15** | 6.89 | 0.33 | 2.57 | 6.04 | 7.75 | 118.84 | 65.62 | 215.23 | 11.25 | 0.24 | 2.57 | 10.64 | 11.87 | 2442.29 | 1591.45 | 3748.02 | 4.97 | 1.06 | 2.57 | 2.25 | 7.68 | 31.26 | 4.76 | 205.43 |
| **VMN12a** | 1.21 | 0.15 | 2.78 | 0.80 | 1.61 | 2.31 | 1.74 | 3.06 | 3.11 | 0.56 | 2.57 | 1.67 | 4.54 | 8.62 | 3.19 | 23.28 | 10.83 | 0.17 | 2.57 | 10.40 | 11.26 | 1816.12 | 1349.51 | 2444.08 |
| **EGY13** | 1.37 | 0.26 | 2.57 | 0.71 | 2.03 | 2.59 | 1.64 | 4.09 | 2.18 | 0.20 | 4.30 | 1.32 | 3.04 | 4.54 | 2.50 | 8.24 | 4.17 | 0.83 | 2.57 | 2.03 | 6.31 | 17.99 | 4.07 | 79.40 |
| **EGY15** | 2.82 | 0.37 | 2.57 | 1.86 | 3.77 | 7.04 | 3.64 | 13.65 | 3.24 | 0.33 | 2.57 | 2.38 | 4.09 | 9.43 | 5.21 | 17.08 | 6.35 | 0.56 | 2.57 | 4.91 | 7.80 | 81.84 | 30.03 | 223.06 |
| **EGY10** | 2.29 | 0.21 | 2.57 | 1.75 | 2.83 | 4.89 | 3.36 | 7.12 | 3.07 | 0.65 | 2.57 | 1.41 | 4.73 | 8.37 | 2.65 | 26.45 | 5.44 | 0.97 | 2.57 | 2.94 | 7.93 | 43.29 | 7.67 | 244.34 |
| **CHN13** | 1.12 | 0.17 | 2.57 | 0.69 | 1.55 | 2.18 | 1.62 | 2.93 | 1.17 | 0.27 | 2.57 | 0.49 | 1.86 | 2.25 | 1.40 | 3.63 | 3.69 | 0.59 | 2.57 | 2.19 | 5.20 | 12.95 | 4.56 | 36.73 |
| **VMN12b** | 1.36 | 0.20 | 2.57 | 0.84 | 1.88 | 2.57 | 1.79 | 3.69 | 2.46 | 0.85 | 2.57 | 0.27 | 4.64 | 5.48 | 1.21 | 24.87 | 9.65 | 0.21 | 2.57 | 9.11 | 10.20 | 806.14 | 553.70 | 1173.68 |
| **IDN10b** | 3.17 | 0.84 | 2.57 | 1.01 | 5.33 | 8.98 | 2.01 | 40.15 | 5.12 | 0.57 | 2.57 | 3.66 | 6.59 | 34.80 | 12.61 | 96.04 | 5.16 | 1.11 | 2.57 | 2.30 | 8.03 | 35.81 | 4.91 | 260.97 |
| **VMN12c** | 1.19 | 0.25 | 3.18 | 0.39 | 1.98 | 2.28 | 1.31 | 3.96 | 1.41 | 0.22 | 2.57 | 0.84 | 1.99 | 2.67 | 1.79 | 3.97 | 6.37 | 0.92 | 2.57 | 4.01 | 8.73 | 82.77 | 16.15 | 424.19 |
| **CHN15a** | 8.07 | 0.54 | 2.57 | 6.67 | 9.46 | 268.14 | 101.97 | 705.11 | 3.84 | 0.62 | 2.57 | 2.25 | 5.44 | 14.36 | 4.76 | 43.27 | 4.14 | 0.49 | 2.57 | 2.86 | 5.41 | 17.57 | 7.28 | 42.40 |
| **RUS18** | 9.78 | 0.48 | 2.57 | 8.55 | 11.01 | 878.89 | 375.51 | 2057.06 | 12.00 | 0.00 | 2.57 | 12.00 | 12.00 | 4096.00 | 4096.00 | 4096.00 | 9.06 | 1.04 | 2.57 | 6.40 | 11.73 | 535.10 | 84.46 | 3390.26 |
| **IRN17** | 6.63 | 0.33 | 2.57 | 5.77 | 7.48 | 98.81 | 54.56 | 178.95 | 8.78 | 0.40 | 2.57 | 7.75 | 9.82 | 440.72 | 215.56 | 901.08 | 6.19 | 0.76 | 2.57 | 4.23 | 8.16 | 73.19 | 18.77 | 285.41 |
| **CHN14** | 4.82 | 0.17 | 2.57 | 4.39 | 5.25 | 28.20 | 20.95 | 37.95 | 7.61 | 0.42 | 2.57 | 6.53 | 8.70 | 195.59 | 92.27 | 414.58 | 4.30 | 0.86 | 2.57 | 2.10 | 6.50 | 19.67 | 4.28 | 90.44 |
| **CHN15b** | 7.89 | 0.58 | 2.57 | 6.41 | 9.38 | 237.81 | 85.01 | 665.27 | 5.35 | 0.50 | 2.57 | 4.06 | 6.63 | 40.66 | 16.68 | 99.09 | 4.74 | 0.68 | 2.57 | 2.98 | 6.50 | 26.72 | 7.91 | 90.26 |
| **TWN17** | 5.88 | 0.52 | 2.57 | 4.55 | 7.20 | 58.73 | 23.40 | 147.38 | 6.65 | 0.21 | 2.57 | 6.11 | 7.19 | 100.38 | 68.95 | 146.15 | 3.14 | 0.76 | 2.57 | 1.19 | 5.09 | 8.82 | 2.28 | 34.17 |
| **CHN16** | 4.64 | 0.21 | 2.57 | 4.10 | 5.18 | 24.96 | 17.14 | 36.34 | 6.95 | 0.37 | 2.57 | 6.01 | 7.89 | 123.82 | 64.60 | 237.32 | 4.89 | 0.55 | 2.78 | 3.37 | 6.41 | 29.68 | 10.34 | 85.16 |
| **VMN20** | 4.61 | 0.33 | 2.57 | 3.75 | 5.47 | 24.42 | 13.48 | 44.23 | 6.13 | 0.31 | 2.57 | 5.34 | 6.92 | 69.90 | 40.43 | 120.87 | 3.68 | 0.65 | 2.57 | 2.00 | 5.37 | 12.85 | 4.01 | 41.22 |
| **CHN21** | 5.73 | 0.73 | 2.57 | 3.85 | 7.60 | 52.96 | 14.42 | 194.58 | 1.57 | 0.68 | 2.57 | -0.19 | 3.33 | 2.97 | 0.88 | 10.03 | 4.61 | 0.33 | 2.57 | 3.75 | 5.47 | 24.42 | 13.48 | 44.23 |

|  | **EGY13** | | | | | | | | **EGY15** | | | | | | | | **EGY10** | | | | | | | |
| --- | --- | --- | --- | --- | --- | --- | --- | --- | --- | --- | --- | --- | --- | --- | --- | --- | --- | --- | --- | --- | --- | --- | --- | --- |
|  | **Mean** | **SE** | **t-value (95% CI)** | **Lower** | **Higher** | **GMT** | **Lower 95% CI** | **Upper 95% CI** | **Mean** | **SE** | **t-value (95% CI)** | **Lower** | **Higher** | **GMT** | **Lower 95% CI** | **Upper 95% CI** | **Mean** | **SE** | **t-value (95% CI)** | **Lower** | **Higher** | **GMT** | **Lower 95% CI** | **Upper 95% CI** |
| **NPL14** | 7.31 | 0.72 | 2.57 | 5.47 | 9.16 | 159.13 | 44.21 | 572.75 | 5.75 | 0.68 | 2.57 | 3.99 | 7.50 | 53.69 | 15.90 | 181.36 | 7.14 | 0.58 | 2.57 | 5.65 | 8.64 | 141.26 | 50.15 | 397.89 |
| **BGD11** | 8.56 | 0.37 | 2.57 | 7.62 | 9.50 | 377.96 | 197.19 | 724.45 | 7.44 | 0.43 | 2.57 | 6.34 | 8.54 | 173.42 | 80.86 | 371.89 | 7.70 | 0.87 | 2.57 | 5.46 | 9.95 | 208.53 | 43.91 | 990.23 |
| **IDN10a** | 5.90 | 0.45 | 2.57 | 4.75 | 7.05 | 59.86 | 26.98 | 132.81 | 5.08 | 0.40 | 2.57 | 4.05 | 6.12 | 33.93 | 16.60 | 69.38 | 6.60 | 0.42 | 2.57 | 5.51 | 7.68 | 96.71 | 45.62 | 204.99 |
| **CHN15b** | 3.72 | 0.23 | 2.57 | 3.12 | 4.32 | 13.20 | 8.71 | 20.01 | 2.99 | 0.48 | 2.57 | 1.77 | 4.22 | 7.97 | 3.40 | 18.64 | 4.23 | 0.42 | 2.57 | 3.15 | 5.32 | 18.82 | 8.88 | 39.88 |
| **USA15** | 7.10 | 0.93 | 2.57 | 4.71 | 9.50 | 137.37 | 26.12 | 722.32 | 4.39 | 0.77 | 2.57 | 2.40 | 6.38 | 20.90 | 5.26 | 83.03 | 5.71 | 0.35 | 2.57 | 4.82 | 6.61 | 52.46 | 28.22 | 97.53 |
| **VMN12a** | 6.55 | 0.37 | 2.57 | 5.61 | 7.49 | 93.76 | 48.91 | 179.70 | 4.42 | 0.56 | 2.57 | 2.99 | 5.86 | 21.47 | 7.95 | 58.00 | 5.41 | 0.43 | 2.57 | 4.31 | 6.52 | 42.65 | 19.89 | 91.47 |
| **EGY13** | 8.83 | 0.17 | 2.57 | 8.40 | 9.25 | 453.53 | 337.00 | 610.34 | 7.22 | 0.56 | 2.57 | 5.78 | 8.65 | 148.62 | 55.01 | 401.51 | 8.37 | 0.46 | 2.57 | 7.18 | 9.56 | 329.99 | 144.60 | 753.09 |
| **EGY15** | 9.62 | 0.42 | 2.57 | 8.53 | 10.70 | 786.23 | 370.92 | 1666.55 | 9.30 | 0.33 | 2.57 | 8.45 | 10.16 | 631.40 | 348.63 | 1143.53 | 9.98 | 0.64 | 2.57 | 8.33 | 11.63 | 1009.36 | 322.52 | 3158.90 |
| **EGY10** | 8.53 | 0.48 | 2.57 | 7.31 | 9.75 | 369.37 | 158.14 | 862.72 | 7.76 | 0.55 | 2.57 | 6.35 | 9.17 | 216.85 | 81.66 | 575.86 | 9.79 | 0.70 | 2.57 | 8.00 | 11.58 | 883.04 | 255.50 | 3051.96 |
| **CHN13** | 4.70 | 0.91 | 2.57 | 2.36 | 7.04 | 25.96 | 5.13 | 131.31 | 2.80 | 0.47 | 2.57 | 1.59 | 4.02 | 6.99 | 3.02 | 16.19 | 4.69 | 1.36 | 2.57 | 1.18 | 8.19 | 25.75 | 2.27 | 292.13 |
| **VMN12b** | 4.28 | 0.71 | 2.57 | 2.45 | 6.11 | 19.48 | 5.48 | 69.26 | 3.93 | 0.46 | 2.57 | 2.75 | 5.11 | 15.23 | 6.73 | 34.47 | 5.34 | 0.35 | 2.57 | 4.44 | 6.24 | 40.50 | 21.70 | 75.59 |
| **IDN10b** | 5.63 | 0.95 | 2.57 | 3.19 | 8.07 | 49.50 | 9.13 | 268.49 | 5.26 | 0.53 | 2.57 | 3.89 | 6.62 | 38.21 | 14.82 | 98.52 | 6.07 | 0.28 | 2.57 | 5.36 | 6.78 | 67.07 | 40.95 | 109.84 |
| **VMN12c** | 5.55 | 1.35 | 2.57 | 2.07 | 9.02 | 46.78 | 4.21 | 520.31 | 5.00 | 0.67 | 2.57 | 3.28 | 6.71 | 31.91 | 9.73 | 104.68 | 7.48 | 0.55 | 2.57 | 6.06 | 8.90 | 178.49 | 66.54 | 478.77 |
| **CHN15a** | 3.90 | 0.21 | 2.57 | 3.36 | 4.44 | 14.96 | 10.27 | 21.77 | 3.24 | 0.40 | 2.57 | 2.20 | 4.28 | 9.42 | 4.58 | 19.38 | 5.65 | 0.21 | 2.57 | 5.10 | 6.19 | 50.08 | 34.40 | 72.91 |
| **RUS18** | 7.59 | 1.43 | 2.57 | 3.92 | 11.27 | 193.10 | 15.11 | 2467.27 | 7.75 | 0.86 | 2.57 | 5.54 | 9.95 | 214.67 | 46.68 | 987.26 | 10.07 | 0.60 | 2.57 | 8.53 | 11.62 | 1078.15 | 369.55 | 3145.48 |
| **IRN17** | 6.21 | 1.02 | 2.57 | 3.59 | 8.84 | 74.26 | 12.02 | 458.75 | 5.06 | 0.76 | 2.57 | 3.11 | 7.01 | 33.38 | 8.61 | 129.31 | 7.72 | 0.31 | 2.57 | 6.92 | 8.52 | 210.83 | 121.44 | 366.00 |
| **CHN14** | 5.56 | 0.75 | 2.57 | 3.63 | 7.48 | 47.03 | 12.38 | 178.66 | 3.49 | 0.73 | 2.57 | 1.61 | 5.37 | 11.22 | 3.05 | 41.21 | 5.97 | 0.26 | 2.57 | 5.31 | 6.64 | 62.77 | 39.62 | 99.43 |
| **CHN15b** | 3.85 | 0.52 | 2.57 | 2.52 | 5.17 | 14.39 | 5.73 | 36.11 | 4.19 | 0.49 | 2.57 | 2.92 | 5.46 | 18.23 | 7.56 | 44.00 | 6.13 | 0.31 | 2.57 | 5.34 | 6.92 | 69.90 | 40.43 | 120.87 |
| **TWN17** | 4.65 | 0.60 | 2.57 | 3.10 | 6.19 | 25.07 | 8.59 | 73.13 | 2.72 | 0.52 | 2.57 | 1.39 | 4.05 | 6.59 | 2.63 | 16.54 | 4.78 | 0.31 | 2.57 | 3.99 | 5.57 | 27.57 | 15.94 | 47.66 |
| **CHN16** | 4.71 | 0.87 | 2.57 | 2.47 | 6.96 | 26.25 | 5.55 | 124.23 | 2.42 | 0.77 | 2.57 | 0.43 | 4.41 | 5.35 | 1.34 | 21.25 | 4.97 | 0.26 | 2.57 | 4.30 | 5.63 | 31.26 | 19.73 | 49.52 |
| **VMN20** | 4.04 | 0.48 | 2.57 | 2.81 | 5.26 | 16.40 | 7.01 | 38.39 | 2.99 | 0.48 | 2.57 | 1.77 | 4.22 | 7.97 | 3.40 | 18.64 | 5.48 | 0.22 | 2.57 | 4.90 | 6.05 | 44.55 | 29.91 | 66.35 |
| **CHN21** | 4.28 | 0.84 | 2.57 | 2.11 | 6.45 | 19.42 | 4.32 | 87.27 | 3.40 | 0.34 | 2.57 | 2.52 | 4.27 | 10.53 | 5.73 | 19.36 | 5.73 | 0.48 | 2.57 | 4.50 | 6.95 | 52.96 | 22.63 | 123.96 |

|  | **CHN13** | | | | | | | | **VMN12b** | | | | | | | | **IDN10b** | | | | | | | |
| --- | --- | --- | --- | --- | --- | --- | --- | --- | --- | --- | --- | --- | --- | --- | --- | --- | --- | --- | --- | --- | --- | --- | --- | --- |
|  | **Mean** | **SE** | **t-value (95% CI)** | **Lower** | **Higher** | **GMT** | **Lower 95% CI** | **Upper 95% CI** | **Mean** | **SE** | **t-value (95% CI)** | **Lower** | **Higher** | **GMT** | **Lower 95% CI** | **Upper 95% CI** | **Mean** | **SE** | **t-value (95% CI)** | **Lower** | **Higher** | **GMT** | **Lower 95% CI** | **Upper 95% CI** |
| **NPL14** | 8.22 | 0.59 | 2.78 | 6.60 | 9.85 | 298.97 | 96.95 | 921.92 | 7.94 | 0.45 | 2.57 | 6.79 | 9.09 | 245.85 | 110.82 | 545.43 | 3.31 | 0.65 | 3.18 | 1.26 | 5.36 | 9.92 | 2.39 | 41.18 |
| **BGD11** | 5.62 | 1.05 | 2.78 | 2.70 | 8.54 | 49.14 | 6.49 | 372.00 | 7.10 | 0.58 | 2.57 | 5.61 | 8.59 | 137.16 | 48.83 | 385.27 | 7.48 | 0.29 | 3.18 | 6.56 | 8.40 | 178.94 | 94.66 | 338.26 |
| **IDN10a** | 5.52 | 0.92 | 2.78 | 2.97 | 8.06 | 45.85 | 7.86 | 267.53 | 12.00 | 0.00 | 2.57 | 12.00 | 12.00 | 4096.00 | 4096.00 | 4096.00 | 6.44 | 0.50 | 3.18 | 4.84 | 8.03 | 86.54 | 28.72 | 260.74 |
| **CHN15b** | 4.40 | 0.28 | 2.78 | 3.63 | 5.18 | 21.17 | 12.38 | 36.18 | 4.94 | 0.60 | 2.57 | 3.41 | 6.48 | 30.77 | 10.62 | 89.17 | 1.41 | 0.50 | 12.71 | -4.94 | 7.77 | 2.67 | 0.03 | 217.87 |
| **USA15** | 5.23 | 0.49 | 2.78 | 3.86 | 6.60 | 37.58 | 14.57 | 96.93 | 7.76 | 0.48 | 2.57 | 6.53 | 8.98 | 216.21 | 92.37 | 506.04 | 7.75 | 1.15 | 3.18 | 4.07 | 11.42 | 214.67 | 16.81 | 2741.48 |
| **VMN12a** | 5.02 | 0.73 | 2.78 | 2.98 | 7.06 | 32.53 | 7.91 | 133.81 | 6.55 | 0.95 | 2.57 | 4.12 | 8.98 | 93.91 | 17.41 | 506.47 | 9.24 | 0.25 | 3.18 | 8.44 | 10.04 | 604.76 | 348.40 | 1049.74 |
| **EGY13** | 5.73 | 1.32 | 2.78 | 2.06 | 9.39 | 52.96 | 4.18 | 670.57 | 5.69 | 0.40 | 2.57 | 4.66 | 6.72 | 51.56 | 25.22 | 105.41 | 3.56 | 0.63 | 3.18 | 1.55 | 5.56 | 11.77 | 2.94 | 47.14 |
| **EGY15** | 6.92 | 1.07 | 2.78 | 3.95 | 9.88 | 120.89 | 15.49 | 943.55 | 6.91 | 0.60 | 2.57 | 5.36 | 8.46 | 120.08 | 41.00 | 351.73 | 5.48 | 0.29 | 3.18 | 4.56 | 6.40 | 44.55 | 23.56 | 84.21 |
| **EGY10** | 5.10 | 1.59 | 2.78 | 0.70 | 9.51 | 34.34 | 1.62 | 727.99 | 5.96 | 0.79 | 2.57 | 3.93 | 8.00 | 62.42 | 15.21 | 256.12 | 5.63 | 0.24 | 3.18 | 4.88 | 6.39 | 49.68 | 29.50 | 83.67 |
| **CHN13** | 10.45 | 1.20 | 2.78 | 7.11 | 13.78 | 1395.54 | 138.61 | 14050.57 | 4.51 | 0.56 | 2.57 | 3.07 | 5.94 | 22.76 | 8.43 | 61.50 | 2.42 | 0.46 | 3.18 | 0.97 | 3.88 | 5.37 | 1.96 | 14.72 |
| **VMN12b** | 4.61 | 0.89 | 2.78 | 2.15 | 7.07 | 24.40 | 4.43 | 134.47 | 6.25 | 0.88 | 2.57 | 4.00 | 8.50 | 76.09 | 15.98 | 362.31 | 8.49 | 0.29 | 3.18 | 7.57 | 9.40 | 358.36 | 189.57 | 677.45 |
| **IDN10b** | 5.72 | 0.89 | 2.78 | 3.24 | 8.21 | 52.85 | 9.45 | 295.54 | 12.00 | 0.00 | 2.57 | 12.00 | 12.00 | 4096.00 | 4096.00 | 4096.00 | 5.21 | 0.96 | 3.18 | 2.16 | 8.25 | 36.93 | 4.47 | 305.23 |
| **VMN12c** | 7.80 | 0.84 | 2.78 | 5.47 | 10.12 | 222.26 | 44.42 | 1112.09 | 7.73 | 0.68 | 2.57 | 5.98 | 9.47 | 212.15 | 63.32 | 710.75 | 10.00 | 0.00 | 3.18 | 10.00 | 10.00 | 1024.00 | 1024.00 | 1024.00 |
| **CHN15a** | 4.04 | 0.58 | 2.78 | 2.42 | 5.66 | 16.48 | 5.37 | 50.62 | 6.32 | 0.21 | 2.57 | 5.77 | 6.86 | 79.69 | 54.74 | 116.02 | 3.83 | 0.71 | 3.18 | 1.58 | 6.08 | 14.26 | 3.00 | 67.84 |
| **RUS18** | 7.50 | 1.07 | 2.78 | 4.54 | 10.47 | 181.25 | 23.22 | 1414.72 | 10.81 | 0.31 | 2.57 | 10.02 | 11.60 | 1797.38 | 1039.51 | 3107.78 | 8.54 | 0.69 | 3.18 | 6.35 | 10.73 | 372.32 | 81.54 | 1700.02 |
| **IRN17** | 5.83 | 0.97 | 2.78 | 3.14 | 8.52 | 56.78 | 8.79 | 366.90 | 9.11 | 0.48 | 2.57 | 7.88 | 10.33 | 551.16 | 235.48 | 1290.00 | 6.62 | 0.75 | 3.18 | 4.23 | 9.01 | 98.33 | 18.80 | 514.25 |
| **CHN14** | 4.30 | 1.02 | 2.78 | 1.47 | 7.13 | 19.70 | 2.77 | 140.20 | 6.76 | 0.48 | 2.57 | 5.53 | 7.98 | 108.23 | 46.24 | 253.32 | 4.93 | 0.58 | 4.30 | 2.45 | 7.42 | 30.54 | 5.46 | 170.85 |
| **CHN15b** | 3.57 | 0.66 | 2.78 | 1.72 | 5.41 | 11.84 | 3.30 | 42.43 | 6.65 | 0.21 | 2.57 | 6.11 | 7.19 | 100.38 | 68.95 | 146.15 | 4.64 | 0.33 | 4.30 | 3.21 | 6.08 | 24.96 | 9.24 | 67.45 |
| **TWN17** | 3.10 | 0.75 | 2.78 | 1.03 | 5.18 | 8.60 | 2.04 | 36.29 | 5.79 | 0.31 | 2.57 | 5.00 | 6.58 | 55.45 | 32.07 | 95.88 | 4.16 | 0.88 | 4.30 | 0.37 | 7.95 | 17.88 | 1.29 | 248.10 |
| **CHN16** | 4.28 | 0.51 | 2.78 | 2.87 | 5.70 | 19.46 | 7.29 | 51.91 | 7.06 | 0.60 | 2.57 | 5.51 | 8.60 | 133.13 | 45.63 | 388.40 | 4.16 | 0.88 | 4.30 | 0.37 | 7.95 | 17.88 | 1.29 | 248.10 |
| **VMN20** | 3.73 | 0.71 | 2.78 | 1.76 | 5.69 | 13.25 | 3.40 | 51.67 | 6.13 | 0.31 | 2.57 | 5.34 | 6.92 | 69.90 | 40.43 | 120.87 | 4.48 | 0.88 | 4.30 | 0.69 | 8.28 | 22.34 | 1.61 | 309.97 |
| **CHN21** | 3.68 | 0.84 | 2.78 | 1.35 | 6.00 | 12.79 | 2.56 | 63.99 | 5.48 | 0.22 | 2.57 | 4.90 | 6.05 | 44.55 | 29.91 | 66.35 | 4.16 | 0.88 | 4.30 | 0.37 | 7.95 | 17.88 | 1.29 | 248.10 |

|  | **VMN12c** | | | | | | | | **CHN15a** | | | | | | | | **RUS18** | | | | | | | |
| --- | --- | --- | --- | --- | --- | --- | --- | --- | --- | --- | --- | --- | --- | --- | --- | --- | --- | --- | --- | --- | --- | --- | --- | --- |
|  | **Mean** | **SE** | **t-value (95% CI)** | **Lower** | **Higher** | **GMT** | **Lower 95% CI** | **Upper 95% CI** | **Mean** | **SE** | **t-value (95% CI)** | **Lower** | **Higher** | **GMT** | **Lower 95% CI** | **Upper 95% CI** | **Mean** | **SE** | **t-value (95% CI)** | **Lower** | **Higher** | **GMT** | **Lower 95% CI** | **Upper 95% CI** |
| **NPL14** | 9.21 | 0.48 | 3.18 | 7.69 | 10.74 | 592.86 | 206.23 | 1704.39 | 4.73 | 0.50 | 2.57 | 3.45 | 6.00 | 26.45 | 10.91 | 64.13 | 3.08 | 0.31 | 3.18 | 2.08 | 4.08 | 8.46 | 4.22 | 16.93 |
| **BGD11** | 7.45 | 0.50 | 3.18 | 5.86 | 9.05 | 175.33 | 58.19 | 528.27 | 6.18 | 0.48 | 2.57 | 4.94 | 7.41 | 72.25 | 30.68 | 170.16 | 5.44 | 0.50 | 3.18 | 3.85 | 7.03 | 43.37 | 14.40 | 130.69 |
| **IDN10a** | 8.49 | 0.29 | 3.18 | 7.57 | 9.40 | 358.36 | 189.57 | 677.45 | 5.99 | 0.51 | 2.57 | 4.67 | 7.31 | 63.73 | 25.54 | 159.01 | 6.24 | 0.25 | 3.18 | 5.44 | 7.03 | 75.36 | 43.42 | 130.81 |
| **CHN15b** | 2.83 | 1.00 | 12.71 | -9.88 | 15.53 | 7.10 | 0.00 | 47466.82 | 11.47 | 0.34 | 2.57 | 10.60 | 12.35 | 2843.09 | 1546.96 | 5225.18 | 4.61 | 0.24 | 3.18 | 3.84 | 5.37 | 24.35 | 14.36 | 41.28 |
| **USA15** | 6.76 | 0.42 | 3.18 | 5.41 | 8.11 | 108.21 | 42.44 | 275.88 | 11.27 | 0.49 | 2.57 | 10.00 | 12.54 | 2475.55 | 1025.85 | 5973.92 | 10.72 | 0.48 | 3.18 | 9.20 | 12.24 | 1685.23 | 586.20 | 4844.77 |
| **VMN12a** | 6.70 | 0.48 | 3.18 | 5.18 | 8.22 | 104.02 | 36.18 | 299.04 | 5.54 | 0.56 | 2.57 | 4.10 | 6.97 | 46.41 | 17.18 | 125.39 | 3.44 | 1.25 | 3.18 | -0.54 | 7.42 | 10.85 | 0.69 | 171.00 |
| **EGY13** | 7.61 | 0.85 | 3.18 | 4.90 | 10.33 | 195.84 | 29.77 | 1288.11 | 3.78 | 0.52 | 2.57 | 2.44 | 5.12 | 13.73 | 5.43 | 34.73 | 2.00 | 0.00 | 4.30 | 2.00 | 2.00 | 4.00 | 4.00 | 4.00 |
| **EGY15** | 8.18 | 0.63 | 3.18 | 6.18 | 10.18 | 290.27 | 72.45 | 1162.88 | 5.71 | 0.37 | 2.57 | 4.77 | 6.65 | 52.34 | 27.23 | 100.59 | 2.78 | 0.85 | 3.18 | 0.07 | 5.50 | 6.88 | 1.05 | 45.28 |
| **EGY10** | 7.80 | 0.76 | 3.18 | 5.38 | 10.21 | 222.41 | 41.65 | 1187.57 | 6.34 | 0.49 | 2.57 | 5.07 | 7.60 | 80.77 | 33.67 | 193.73 | 3.34 | 1.00 | 3.18 | 0.16 | 6.53 | 10.15 | 1.12 | 92.17 |
| **CHN13** | 8.10 | 0.51 | 3.18 | 6.49 | 9.71 | 274.78 | 90.04 | 838.58 | 2.45 | 0.22 | 2.57 | 1.87 | 3.02 | 5.46 | 3.67 | 8.14 | 0.84 | 0.13 | 3.18 | 0.44 | 1.24 | 1.79 | 1.36 | 2.36 |
| **VMN12b** | 4.90 | 0.58 | 3.18 | 3.06 | 6.74 | 29.84 | 8.35 | 106.62 | 5.58 | 0.42 | 2.57 | 4.50 | 6.67 | 47.89 | 22.59 | 101.51 | 6.30 | 0.96 | 3.18 | 3.25 | 9.35 | 78.78 | 9.53 | 651.07 |
| **IDN10b** | 8.32 | 0.96 | 3.18 | 5.28 | 11.37 | 320.37 | 38.76 | 2647.74 | 4.34 | 0.38 | 2.57 | 3.36 | 5.33 | 20.31 | 10.24 | 40.29 | 1.97 | 0.96 | 3.18 | -1.08 | 5.01 | 3.91 | 0.47 | 32.33 |
| **VMN12c** | 11.49 | 0.29 | 3.18 | 10.57 | 12.41 | 2874.56 | 1520.61 | 5434.07 | 6.18 | 0.47 | 2.57 | 4.98 | 7.38 | 72.44 | 31.54 | 166.38 | 5.00 | 0.00 | 3.18 | 5.00 | 5.00 | 32.00 | 32.00 | 32.00 |
| **CHN15a** | 4.85 | 0.42 | 3.18 | 3.51 | 6.18 | 28.78 | 11.39 | 72.74 | 10.47 | 0.34 | 2.57 | 9.59 | 11.35 | 1418.88 | 772.03 | 2607.69 | 6.74 | 0.25 | 3.18 | 5.94 | 7.53 | 106.55 | 61.38 | 184.95 |
| **RUS18** | 7.07 | 0.52 | 3.18 | 5.43 | 8.71 | 134.40 | 43.12 | 418.93 | 10.09 | 0.54 | 2.57 | 8.69 | 11.48 | 1088.62 | 413.98 | 2862.68 | 9.46 | 0.50 | 3.18 | 7.87 | 11.05 | 703.02 | 233.32 | 2118.22 |
| **IRN17** | 5.48 | 0.29 | 3.18 | 4.56 | 6.40 | 44.55 | 23.56 | 84.21 | 7.65 | 0.21 | 2.57 | 7.11 | 8.19 | 201.09 | 138.12 | 292.78 | 8.21 | 0.48 | 3.18 | 6.68 | 9.73 | 295.43 | 102.77 | 849.33 |
| **CHN14** | 4.95 | 0.41 | 3.18 | 3.65 | 6.25 | 30.89 | 12.55 | 76.03 | 7.32 | 0.21 | 2.57 | 6.78 | 7.86 | 159.63 | 109.64 | 232.41 | 6.40 | 0.65 | 3.18 | 4.35 | 8.46 | 84.58 | 20.36 | 351.27 |
| **CHN15b** | 4.68 | 0.48 | 3.18 | 3.16 | 6.20 | 25.65 | 8.92 | 73.73 | 8.98 | 0.26 | 2.57 | 8.32 | 9.65 | 505.44 | 319.06 | 800.69 | 3.83 | 1.25 | 3.18 | -0.14 | 7.81 | 14.26 | 0.90 | 224.68 |
| **TWN17** | 4.47 | 0.29 | 3.18 | 3.55 | 5.39 | 22.19 | 11.74 | 41.96 | 7.13 | 0.31 | 2.57 | 6.34 | 7.92 | 140.36 | 81.18 | 242.69 | 4.61 | 0.63 | 3.18 | 2.60 | 6.61 | 24.35 | 6.08 | 97.55 |
| **CHN16** | 5.23 | 0.25 | 3.18 | 4.44 | 6.03 | 37.61 | 21.67 | 65.29 | 6.45 | 0.34 | 2.57 | 5.57 | 7.33 | 87.45 | 47.58 | 160.72 | 6.45 | 0.50 | 3.18 | 4.86 | 8.04 | 87.27 | 28.96 | 262.95 |
| **VMN20** | 4.47 | 0.29 | 3.18 | 3.55 | 5.39 | 22.19 | 11.74 | 41.96 | 7.29 | 0.33 | 2.57 | 6.44 | 8.15 | 156.88 | 86.62 | 284.12 | 6.29 | 0.87 | 3.18 | 3.54 | 9.05 | 78.40 | 11.60 | 529.60 |
| **CHN21** | 4.47 | 0.29 | 3.18 | 3.55 | 5.39 | 22.19 | 11.74 | 41.96 | 7.46 | 0.34 | 2.57 | 6.59 | 8.34 | 176.52 | 96.05 | 324.42 | 2.99 | 0.87 | 3.18 | 0.23 | 5.75 | 7.95 | 1.18 | 53.70 |

|  | **IRN17** | | | | | | | | **CHN14** | | | | | | | | **CHN15b** | | | | | | | |
| --- | --- | --- | --- | --- | --- | --- | --- | --- | --- | --- | --- | --- | --- | --- | --- | --- | --- | --- | --- | --- | --- | --- | --- | --- |
|  | **Mean** | **SE** | **t-value (95% CI)** | **Lower** | **Higher** | **GMT** | **Lower 95% CI** | **Upper 95% CI** | **Mean** | **SE** | **t-value (95% CI)** | **Lower** | **Higher** | **GMT** | **Lower 95% CI** | **Upper 95% CI** | **Mean** | **SE** | **t-value (95% CI)** | **Lower** | **Higher** | **GMT** | **Lower 95% CI** | **Upper 95% CI** |
| **NPL14** | 4.68 | 0.48 | 3.18 | 3.16 | 6.20 | 25.65 | 8.92 | 73.73 | 4.56 | 0.42 | 2.57 | 3.48 | 5.64 | 23.60 | 11.13 | 50.03 | 2.70 | 0.37 | 2.78 | 1.66 | 3.74 | 6.51 | 3.17 | 13.37 |
| **BGD11** | 6.67 | 0.92 | 3.18 | 3.74 | 9.61 | 102.14 | 13.38 | 779.65 | 7.27 | 0.42 | 2.57 | 6.19 | 8.36 | 154.81 | 73.04 | 328.16 | 4.23 | 0.60 | 2.78 | 2.56 | 5.89 | 18.73 | 5.90 | 59.43 |
| **IDN10a** | 8.35 | 0.38 | 3.18 | 7.16 | 9.54 | 326.17 | 142.62 | 745.93 | 6.29 | 0.33 | 2.57 | 5.43 | 7.14 | 78.07 | 43.11 | 141.40 | 4.13 | 0.37 | 2.78 | 3.09 | 5.17 | 17.50 | 8.52 | 35.95 |
| **CHN15b** | 6.27 | 0.63 | 3.18 | 4.28 | 8.26 | 77.06 | 19.41 | 305.92 | 6.74 | 0.48 | 2.57 | 5.52 | 7.97 | 107.19 | 45.80 | 250.87 | 9.35 | 0.51 | 2.78 | 7.93 | 10.76 | 650.46 | 243.81 | 1735.36 |
| **USA15** | 11.49 | 0.29 | 3.18 | 10.57 | 12.41 | 2874.56 | 1520.61 | 5434.07 | 11.92 | 0.08 | 2.57 | 11.70 | 12.13 | 3862.13 | 3329.22 | 4480.35 | 8.56 | 0.40 | 2.78 | 7.45 | 9.68 | 378.59 | 175.33 | 817.51 |
| **VMN12a** | 4.95 | 0.41 | 3.18 | 3.65 | 6.25 | 30.89 | 12.55 | 76.03 | 4.11 | 0.31 | 2.57 | 3.32 | 4.90 | 17.23 | 9.97 | 29.80 | 3.10 | 0.37 | 2.78 | 2.06 | 4.14 | 8.60 | 4.18 | 17.66 |
| **EGY13** | 2.45 | 0.29 | 3.18 | 1.53 | 3.37 | 5.46 | 2.89 | 10.33 | 2.94 | 0.61 | 2.57 | 1.36 | 4.52 | 7.65 | 2.56 | 22.87 | 2.88 | 0.58 | 4.30 | 0.40 | 5.37 | 7.38 | 1.32 | 41.32 |
| **EGY15** | 3.72 | 0.25 | 3.18 | 2.93 | 4.52 | 13.20 | 7.60 | 22.91 | 4.49 | 0.71 | 2.57 | 2.65 | 6.33 | 22.47 | 6.28 | 80.31 | 3.73 | 0.92 | 2.78 | 1.18 | 6.27 | 13.25 | 2.27 | 77.31 |
| **EGY10** | 3.08 | 0.63 | 3.18 | 1.08 | 5.08 | 8.46 | 2.11 | 33.88 | 5.43 | 0.68 | 2.78 | 3.54 | 7.31 | 43.00 | 11.66 | 158.60 | 3.13 | 0.97 | 2.78 | 0.44 | 5.82 | 8.75 | 1.35 | 56.53 |
| **CHN13** | 1.32 | 0.55 | 3.18 | -0.45 | 3.08 | 2.49 | 0.73 | 8.46 | 2.55 | 0.24 | 2.78 | 1.87 | 3.23 | 5.86 | 3.66 | 9.39 | 1.00 | 0.00 | 4.30 | 1.00 | 1.00 | 2.00 | 2.00 | 2.00 |
| **VMN12b** | 7.32 | 0.55 | 3.18 | 5.55 | 9.08 | 159.46 | 46.95 | 541.66 | 5.69 | 0.36 | 2.57 | 4.77 | 6.62 | 51.71 | 27.26 | 98.10 | 4.46 | 0.32 | 2.78 | 3.58 | 5.34 | 21.97 | 11.96 | 40.38 |
| **IDN10b** | 8.37 | 0.87 | 3.18 | 5.61 | 11.12 | 330.06 | 48.86 | 2229.77 | 6.08 | 0.48 | 2.57 | 4.85 | 7.30 | 67.54 | 28.86 | 158.08 | 4.02 | 0.40 | 2.78 | 2.91 | 5.14 | 16.28 | 7.54 | 35.15 |
| **VMN12c** | 5.69 | 0.48 | 3.18 | 4.17 | 7.22 | 51.71 | 17.99 | 148.66 | 4.56 | 0.42 | 2.57 | 3.48 | 5.64 | 23.60 | 11.13 | 50.03 | 2.49 | 0.40 | 2.78 | 1.38 | 3.60 | 5.62 | 2.60 | 12.14 |
| **CHN15a** | 6.87 | 0.13 | 3.18 | 6.47 | 7.27 | 117.09 | 88.87 | 154.27 | 7.71 | 0.36 | 2.57 | 6.78 | 8.63 | 209.04 | 110.18 | 396.59 | 8.54 | 0.51 | 2.78 | 7.12 | 9.95 | 371.59 | 139.28 | 991.38 |
| **RUS18** | 10.67 | 0.75 | 3.18 | 8.28 | 13.06 | 1628.89 | 311.45 | 8519.25 | 11.00 | 0.00 | 2.57 | 11.00 | 11.00 | 2048.00 | 2048.00 | 2048.00 | 9.35 | 0.51 | 2.78 | 7.93 | 10.76 | 650.46 | 243.81 | 1735.36 |
| **IRN17** | 9.87 | 0.91 | 3.18 | 6.97 | 12.78 | 938.12 | 125.23 | 7027.46 | 8.98 | 0.26 | 2.57 | 8.32 | 9.65 | 505.44 | 319.06 | 800.69 | 7.16 | 0.37 | 2.78 | 6.12 | 8.20 | 143.00 | 69.60 | 293.81 |
| **CHN14** | 8.71 | 0.48 | 3.18 | 7.19 | 10.24 | 419.28 | 145.84 | 1205.35 | 11.83 | 0.17 | 2.57 | 11.40 | 12.26 | 3633.72 | 2700.11 | 4890.15 | 7.58 | 0.24 | 2.78 | 6.90 | 8.26 | 191.86 | 119.75 | 307.41 |
| **CHN15b** | 6.74 | 0.25 | 3.18 | 5.94 | 7.53 | 106.55 | 61.38 | 184.95 | 8.60 | 0.49 | 2.57 | 7.32 | 9.87 | 386.72 | 160.25 | 933.22 | 9.59 | 0.24 | 2.78 | 8.91 | 10.27 | 769.25 | 480.11 | 1232.53 |
| **TWN17** | 6.70 | 0.48 | 3.18 | 5.18 | 8.22 | 104.02 | 36.18 | 299.04 | 10.11 | 0.48 | 2.57 | 8.88 | 11.33 | 1101.45 | 470.60 | 2577.99 | 8.56 | 0.40 | 2.78 | 7.45 | 9.68 | 378.59 | 175.33 | 817.51 |
| **CHN16** | 8.63 | 0.85 | 3.18 | 5.91 | 11.35 | 395.88 | 60.19 | 2603.86 | 9.62 | 0.42 | 2.57 | 8.53 | 10.70 | 786.23 | 370.92 | 1666.55 | 6.76 | 0.37 | 2.78 | 5.72 | 7.80 | 108.35 | 52.74 | 222.61 |
| **VMN20** | 8.35 | 0.96 | 3.18 | 5.30 | 11.39 | 325.30 | 39.36 | 2688.46 | 11.66 | 0.21 | 2.57 | 11.12 | 12.20 | 3229.18 | 2217.97 | 4701.42 | 6.92 | 0.55 | 2.78 | 5.40 | 8.44 | 121.15 | 42.22 | 347.63 |
| **CHN21** | 5.18 | 0.48 | 3.18 | 3.66 | 6.70 | 36.25 | 12.61 | 104.22 | 6.82 | 0.17 | 2.57 | 6.39 | 7.25 | 113.18 | 84.10 | 152.31 | 6.76 | 0.37 | 2.78 | 5.72 | 7.80 | 108.35 | 52.74 | 222.61 |

|  | **TWN17** | | | | | | | | **CHN16** | | | | | | | | **VMN20** | | | | | | | |
| --- | --- | --- | --- | --- | --- | --- | --- | --- | --- | --- | --- | --- | --- | --- | --- | --- | --- | --- | --- | --- | --- | --- | --- | --- |
|  | **Mean** | **SE** | **t-value (95% CI)** | **Lower** | **Higher** | **GMT** | **Lower 95% CI** | **Upper 95% CI** | **Mean** | **SE** | **t-value (95% CI)** | **Lower** | **Higher** | **GMT** | **Lower 95% CI** | **Upper 95% CI** | **Mean** | **SE** | **t-value (95% CI)** | **Lower** | **Higher** | **GMT** | **Lower 95% CI** | **Upper 95% CI** |
| **NPL14** | 2.29 | 0.21 | 2.57 | 1.75 | 2.83 | 4.89 | 3.36 | 7.12 | 3.66 | 0.48 | 3.18 | 2.14 | 5.19 | 12.67 | 4.41 | 36.41 | 4.39 | 1.08 | 2.57 | 1.62 | 7.16 | 21.03 | 3.08 | 143.43 |
| **BGD11** | 4.13 | 0.63 | 2.57 | 2.52 | 5.74 | 17.51 | 5.72 | 53.60 | 5.23 | 0.25 | 3.18 | 4.44 | 6.03 | 37.61 | 21.67 | 65.29 | 5.79 | 0.31 | 2.57 | 5.00 | 6.58 | 55.45 | 32.07 | 95.88 |
| **IDN10a** | 3.65 | 0.71 | 2.57 | 1.82 | 5.49 | 12.58 | 3.52 | 44.96 | 5.18 | 1.04 | 3.18 | 1.87 | 8.49 | 36.25 | 3.65 | 360.15 | 6.48 | 0.67 | 2.57 | 4.76 | 8.19 | 88.98 | 27.13 | 291.87 |
| **CHN15b** | 7.66 | 0.93 | 2.57 | 5.26 | 10.05 | 201.89 | 38.43 | 1060.47 | 4.74 | 0.91 | 3.18 | 1.83 | 7.64 | 26.69 | 3.56 | 199.92 | 5.79 | 0.31 | 2.57 | 5.00 | 6.58 | 55.45 | 32.07 | 95.88 |
| **USA15** | 9.25 | 0.56 | 2.57 | 7.82 | 10.69 | 609.77 | 225.71 | 1647.35 | 10.98 | 0.41 | 3.18 | 9.68 | 12.28 | 2015.89 | 819.14 | 4961.05 | 11.66 | 0.21 | 2.57 | 11.12 | 12.20 | 3229.18 | 2217.97 | 4701.42 |
| **VMN12a** | 1.35 | 0.20 | 2.57 | 0.83 | 1.86 | 2.55 | 1.78 | 3.64 | 3.08 | 0.63 | 3.18 | 1.08 | 5.08 | 8.46 | 2.11 | 33.88 | 3.96 | 0.26 | 2.57 | 3.29 | 4.62 | 15.53 | 9.80 | 24.61 |
| **EGY13** | 1.64 | 0.37 | 2.78 | 0.60 | 2.68 | 3.12 | 1.52 | 6.42 | 2.91 | 0.41 | 3.18 | 1.61 | 4.21 | 7.53 | 3.06 | 18.53 | 2.99 | 0.56 | 2.57 | 1.56 | 4.43 | 7.97 | 2.95 | 21.52 |
| **EGY15** | 1.44 | 0.18 | 2.57 | 0.97 | 1.91 | 2.72 | 1.96 | 3.76 | 3.22 | 0.25 | 3.18 | 2.43 | 4.02 | 9.34 | 5.38 | 16.22 | 4.88 | 0.27 | 2.57 | 4.18 | 5.58 | 29.44 | 18.15 | 47.74 |
| **EGY10** | 2.22 | 0.40 | 2.78 | 1.11 | 3.33 | 4.66 | 2.16 | 10.06 | 2.91 | 0.41 | 3.18 | 1.61 | 4.21 | 7.53 | 3.06 | 18.53 | 4.31 | 0.21 | 2.57 | 3.77 | 4.85 | 19.82 | 13.61 | 28.86 |
| **CHN13** | 1.00 | 0.00 | 12.71 | 1.00 | 1.00 | 2.00 | 2.00 | 2.00 | 2.51 | 0.75 | 3.18 | 0.13 | 4.90 | 5.72 | 1.09 | 29.89 | 3.36 | 1.13 | 2.57 | 0.47 | 6.25 | 10.27 | 1.38 | 76.29 |
| **VMN12b** | 2.31 | 0.41 | 2.57 | 1.26 | 3.35 | 4.94 | 2.39 | 10.23 | 4.12 | 0.63 | 3.18 | 2.12 | 6.12 | 17.38 | 4.34 | 69.64 | 5.05 | 0.27 | 2.57 | 4.35 | 5.74 | 33.03 | 20.37 | 53.56 |
| **IDN10b** | 3.23 | 0.95 | 2.57 | 0.78 | 5.68 | 9.38 | 1.72 | 51.17 | 5.57 | 0.85 | 3.18 | 2.85 | 8.28 | 47.38 | 7.20 | 311.66 | 6.26 | 0.61 | 2.57 | 4.68 | 7.83 | 76.39 | 25.71 | 226.99 |
| **VMN12c** | 1.44 | 0.33 | 2.57 | 0.60 | 2.28 | 2.72 | 1.52 | 4.87 | 3.60 | 0.83 | 3.18 | 0.97 | 6.23 | 12.13 | 1.96 | 75.01 | 4.78 | 1.01 | 2.57 | 2.18 | 7.39 | 27.57 | 4.53 | 167.83 |
| **CHN15a** | 7.25 | 0.49 | 2.57 | 5.98 | 8.52 | 152.16 | 63.05 | 367.18 | 6.45 | 0.50 | 3.18 | 4.86 | 8.04 | 87.27 | 28.96 | 262.95 | 7.98 | 0.26 | 2.57 | 7.32 | 8.64 | 252.31 | 159.27 | 399.69 |
| **RUS18** | 8.98 | 0.61 | 2.57 | 7.41 | 10.55 | 504.47 | 169.76 | 1499.06 | 11.00 | 0.00 | 3.18 | 11.00 | 11.00 | 2048.00 | 2048.00 | 2048.00 | 10.66 | 0.21 | 2.57 | 10.11 | 11.20 | 1613.55 | 1108.27 | 2349.19 |
| **IRN17** | 7.15 | 0.54 | 2.57 | 5.76 | 8.55 | 142.42 | 54.04 | 375.38 | 9.46 | 0.50 | 3.18 | 7.87 | 11.05 | 703.02 | 233.32 | 2118.22 | 10.11 | 0.48 | 2.57 | 8.89 | 11.34 | 1106.77 | 472.87 | 2590.44 |
| **CHN14** | 6.78 | 0.40 | 2.57 | 5.74 | 7.81 | 109.57 | 53.59 | 224.02 | 9.46 | 0.50 | 3.18 | 7.87 | 11.05 | 703.02 | 233.32 | 2118.22 | 9.81 | 0.31 | 2.57 | 9.02 | 10.60 | 897.32 | 518.97 | 1551.53 |
| **CHN15b** | 7.61 | 0.42 | 2.57 | 6.52 | 8.69 | 194.75 | 91.88 | 412.81 | 6.70 | 0.48 | 3.18 | 5.18 | 8.22 | 104.02 | 36.18 | 299.04 | 7.65 | 0.21 | 2.57 | 7.11 | 8.19 | 201.09 | 138.12 | 292.78 |
| **TWN17** | 8.98 | 0.26 | 2.57 | 8.32 | 9.65 | 505.44 | 319.06 | 800.69 | 7.91 | 0.71 | 3.18 | 5.66 | 10.16 | 240.96 | 50.64 | 1146.46 | 7.48 | 0.22 | 2.57 | 6.91 | 8.06 | 178.94 | 120.14 | 266.52 |
| **CHN16** | 7.11 | 0.40 | 2.57 | 6.08 | 8.14 | 138.00 | 67.50 | 282.15 | 11.24 | 0.25 | 3.18 | 10.45 | 12.04 | 2421.87 | 1395.23 | 4203.91 | 8.78 | 0.40 | 2.57 | 7.75 | 9.82 | 440.72 | 215.56 | 901.08 |
| **VMN20** | 6.29 | 0.33 | 2.57 | 5.43 | 7.14 | 78.07 | 43.11 | 141.40 | 8.43 | 0.65 | 3.18 | 6.37 | 10.48 | 343.87 | 82.79 | 1428.21 | 10.64 | 0.33 | 2.57 | 9.78 | 11.50 | 1597.16 | 881.88 | 2892.61 |
| **CHN21** | 4.59 | 0.33 | 2.57 | 3.74 | 5.45 | 24.12 | 13.32 | 43.68 | 5.23 | 0.25 | 3.18 | 4.44 | 6.03 | 37.61 | 21.67 | 65.29 | 6.32 | 0.21 | 2.57 | 5.77 | 6.86 | 79.69 | 54.74 | 116.02 |

|  | **CHN21** | | | | | | | |
| --- | --- | --- | --- | --- | --- | --- | --- | --- |
|  | **Mean** | **SE** | **t-value (95% CI)** | **Lower** | **Higher** | **GMT** | **Lower 95% CI** | **Upper 95% CI** |
| **NPL14** | 3.96 | 0.26 | 2.57 | 3.29 | 4.62 | 15.53 | 9.80 | 24.61 |
| **BGD11** | 5.41 | 0.43 | 2.57 | 4.31 | 6.52 | 42.65 | 19.89 | 91.47 |
| **IDN10a** | 4.95 | 0.65 | 2.57 | 3.27 | 6.63 | 30.90 | 9.63 | 99.09 |
| **CHN15b** | 7.91 | 0.52 | 2.57 | 6.58 | 9.24 | 240.52 | 95.84 | 603.61 |
| **USA15** | 4.97 | 0.60 | 2.57 | 3.43 | 6.52 | 31.40 | 10.76 | 91.61 |
| **VMN12a** | 2.50 | 0.33 | 2.57 | 1.64 | 3.35 | 5.65 | 3.12 | 10.23 |
| **EGY13** | 4.11 | 0.31 | 2.57 | 3.32 | 4.90 | 17.23 | 9.97 | 29.80 |
| **EGY15** | 5.73 | 0.48 | 2.57 | 4.50 | 6.95 | 52.96 | 22.63 | 123.96 |
| **EGY10** | 5.15 | 0.17 | 2.57 | 4.73 | 5.58 | 35.61 | 26.46 | 47.92 |
| **CHN13** | 2.35 | 0.24 | 2.78 | 1.67 | 3.03 | 5.11 | 3.19 | 8.18 |
| **VMN12b** | 3.81 | 0.17 | 2.57 | 3.38 | 4.24 | 14.05 | 10.44 | 18.91 |
| **IDN10b** | 3.80 | 0.42 | 2.57 | 2.73 | 4.87 | 13.91 | 6.62 | 29.22 |
| **VMN12c** | 3.77 | 0.31 | 2.57 | 2.98 | 4.56 | 13.66 | 7.90 | 23.62 |
| **CHN15a** | 8.65 | 0.21 | 2.57 | 8.11 | 9.20 | 402.68 | 276.58 | 586.27 |
| **RUS18** | 7.46 | 0.34 | 2.57 | 6.58 | 8.34 | 175.78 | 95.65 | 323.06 |
| **IRN17** | 6.48 | 0.22 | 2.57 | 5.91 | 7.06 | 89.31 | 59.96 | 133.02 |
| **CHN14** | 6.48 | 0.22 | 2.57 | 5.91 | 7.06 | 89.31 | 59.96 | 133.02 |
| **CHN15b** | 6.45 | 0.34 | 2.57 | 5.57 | 7.33 | 87.45 | 47.58 | 160.72 |
| **TWN17** | 7.13 | 0.31 | 2.57 | 6.34 | 7.92 | 140.36 | 81.18 | 242.69 |
| **CHN16** | 5.62 | 0.33 | 2.57 | 4.76 | 6.48 | 49.17 | 27.15 | 89.06 |
| **VMN20** | 6.16 | 0.17 | 2.57 | 5.73 | 6.58 | 71.32 | 52.99 | 95.97 |
| **CHN21** | 10.66 | 0.21 | 2.57 | 10.11 | 11.20 | 1613.55 | 1108.27 | 2349.19 |
