# Supplementary material for: Mapping hemagglutinin residues driving antigenic diversity in H5Nx avian influenza viruses
Source: J Virol. 2026 Apr 30;100(6):e00095-26. doi: 10.1128/jvi.00095-26 (PMC13288987; doi:10.1128/jvi.00095-26)
Supplement: Table S3 — Pairwise comparisons of antigenic and genetic variances between the study strains, sorted in decreasing values of antigenic distance per genetic distance (ratio). [file jvi.00095-26-s0005.docx]

**Table S3:** Pairwise comparisons of antigenic and genetic variances between the study strains, sorted in decreasing values of antigenic distance per genetic distance (ratio). Antigenic distances are the total antigenic units from 2-dimensional and 3-dimensional antigenic cartography. Genetic distance was calculated as the total amino acid variance between each HA1. Highest n=28 ratios for each dimension shown.

| **Strain Comparison (2D)** | **Antigenic Distance** | **Genetic Distance** | **Ratio** | **Strain Comparison (3D)** | **Antigenic Distance** | **Genetic Distance** | **Ratio** |
| --- | --- | --- | --- | --- | --- | --- | --- |
|  |  |  |  |  |  |  |  |
| A/TURKEY/EGYPT/137/2013 vs A/EGYPT/N0001/2015 | 1.434 | 3 | 0.478 | A/YUNNAN/0127/2015 vs A/DUCK/TAIWAN/1702004/2017 | 3.76 | 7 | 0.537 |
| A/CHICKEN/CHEBOKSARY/854/2018 vs A/CHICKEN/SICHUAN/J1/2014 | 2.817 | 8 | 0.352 | A/TURKEY/EGYPT/137/2013 vs A/EGYPT/N0001/2015 | 1.473 | 3 | 0.491 |
| A/YUNNAN/0127/2015 vs A/WHOOPER SWAN/HUNAN/4/2016 | 3.799 | 11 | 0.345 | A/YUNNAN/0127/2015 vs A/WHOOPER SWAN/HUNAN/4/2016 | 5.377 | 11 | 0.489 |
| A/YUNNAN/0127/2015 vs A/DUCK/TAIWAN/1702004/2017 | 2.356 | 7 | 0.337 | A/YUNNAN/0127/2015 vs A/CHICKEN/VIETNAM/RAH04-CD-20-421/2020 | 4.825 | 11 | 0.439 |
| A/YUNNAN/0127/2015 vs A/CHICKEN/VIETNAM/RAH04-CD-20-421/2020 | 3.562 | 11 | 0.324 | A/YUNNAN/0127/2015 vs A/CHICKEN/SICHUAN/J1/2014 | 4.811 | 13 | 0.37 |
| A/YUNNAN/0127/2015 vs A/CHICKEN/IOWA/14589-1/2015 | 4.089 | 13 | 0.315 | A/CHICKEN/GANZHOU/GZ21/2015 vs A/WHOOPER SWAN/HUNAN/4/2016 | 4.71 | 14 | 0.336 |
| A/DUCK/NANCHANG/9789/2013 vs A/DUCK/TAIWAN/1702004/2017 | 10.577 | 36 | 0.294 | A/CHICKEN/CHEBOKSARY/854/2018 vs A/CHICKEN/SICHUAN/J1/2014 | 2.578 | 8 | 0.322 |
| A/DUCK/NANCHANG/9789/2013 vs A/WHOOPER SWAN/HUNAN/4/2016 | 10.828 | 37 | 0.293 | A/YUNNAN/0127/2015 vs A/CHICKEN/IOWA/14589-1/2015 | 3.967 | 13 | 0.305 |
| A/YUNNAN/0127/2015 vs A/CHICKEN/CHEBOKSARY/854/2018 | 4.362 | 15 | 0.291 | A/DUCK/VIETNAM/OIE/2002/2012 vs A/CHICKEN/VIETNAM/NCVD/1192-2012 | 2.129 | 7 | 0.304 |
| A/YUNNAN/0127/2015 vs A/CROWIAGHAKHAN/2017 | 4.613 | 16 | 0.288 | A/CHICKEN/GANZHOU/GZ21/2015 vs A/CHICKEN/VIETNAM/RAH04-CD-20-421/2020 | 4.178 | 14 | 0.298 |
| A/YUNNAN/0127/2015 vs A/CHICKEN/SICHUAN/J1/2014 | 3.672 | 13 | 0.282 | A/YUNNAN/0127/2015 vs A/CROWIAGHAKHAN/2017 | 4.645 | 16 | 0.29 |
| A/TURKEY/EGYPT/137/2013 vs A/DUCK/TAIWAN/1702004/2017 | 9.913 | 36 | 0.275 | A/DUCK/NANCHANG/9789/2013 vs A/DUCK/TAIWAN/1702004/2017 | 10.249 | 36 | 0.285 |
| A/DUCK/NANCHANG/9789/2013 vs A/CHICKEN/SICHUAN/J1/2014 | 10.142 | 37 | 0.274 | A/CHICKEN/GANZHOU/GZ21/2015 vs A/DUCK/TAIWAN/1702004/2017 | 3.11 | 11 | 0.283 |
| A/TURKEY/EGYPT/137/2013 vs A/WHOOPER SWAN/HUNAN/4/2016 | 9.81 | 36 | 0.273 | A/DUCK/NANCHANG/9789/2013 vs A/WHOOPER SWAN/HUNAN/4/2016 | 10.212 | 37 | 0.276 |
| A/DUCK/NANCHANG/9789/2013 vs A/CHICKEN/VIETNAM/RAH04-CD-20-421/2020 | 10.814 | 40 | 0.27 | A/YUNNAN/0127/2015 vs A/CHICKEN/CHEBOKSARY/854/2018 | 4.108 | 15 | 0.274 |
| A/CHICKEN/NEPAL/T-359/2014 vs A/CHICKEN/SICHUAN/J1/2014 | 8.086 | 30 | 0.27 | A/CHICKEN/GANZHOU/GZ21/2015 vs A/CHICKEN/SICHUAN/J1/2014 | 4.092 | 15 | 0.273 |
| A/TURKEY/EGYPT/137/2013 vs A/CHICKEN/SICHUAN/J1/2014 | 9.092 | 34 | 0.267 | A/CHICKEN/BANGLADESH/31289-1/2011 vs A/TURKEY/EGYPT/137/2013 | 3.798 | 14 | 0.271 |
| A/CHICKEN/NEPAL/T-359/2014 vs A/DUCK/TAIWAN/1702004/2017 | 8.976 | 35 | 0.256 | A/DUCK/NANCHANG/9789/2013 vs A/CHICKEN/VIETNAM/RAH04-CD-20-421/2020 | 10.306 | 40 | 0.258 |
| A/CHICKEN/GANZHOU/GZ21/2015 vs A/CHICKEN/CHEBOKSARY/854/2018 | 3.737 | 15 | 0.249 | A/DUCK/NANCHANG/9789/2013 vs A/CHICKEN/SICHUAN/J1/2014 | 9.519 | 37 | 0.257 |
| A/CHICKEN/CHEBOKSARY/854/2018 vs A/DUCK/TAIWAN/1702004/2017 | 3.962 | 16 | 0.248 | A/CHICKEN/GANZHOU/GZ21/2015 vs A/CROWIAGHAKHAN/2017 | 4.017 | 16 | 0.251 |
| A/CHICKEN/NEPAL/T-359/2014 vs A/WHOOPER SWAN/HUNAN/4/2016 | 8.807 | 36 | 0.245 | A/CHICKEN/BANGLADESH/31289-1/2011 vs A/EGYPT/N0001/2015 | 2.71 | 11 | 0.246 |
| A/CHICKEN/GANZHOU/GZ21/2015 vs A/CROWIAGHAKHAN/2017 | 3.899 | 16 | 0.244 | A/ANHUI/33162/2016 vs A/WHOOPER SWAN/HUNAN/4/2016 | 3.683 | 15 | 0.246 |
| A/DUCK/NANCHANG/9789/2013 vs A/CROWIAGHAKHAN/2017 | 8.496 | 35 | 0.243 | A/CHICKEN/IOWA/14589-1/2015 vs A/DUCK/TAIWAN/1702004/2017 | 3.162 | 13 | 0.243 |
| A/TURKEY/EGYPT/137/2013 vs A/ANHUI/33162/2016 | 9.167 | 38 | 0.241 | A/TURKEY/EGYPT/137/2013 vs A/CHICKEN/VIETNAM/NCVD/1192-2012 | 6.264 | 26 | 0.241 |
| A/TURKEY/EGYPT/137/2013 vs A/CHONGQING/00013/2021 | 9.401 | 39 | 0.241 | A/CHICKEN/NEPAL/T-359/2014 vs A/CHICKEN/SICHUAN/J1/2014 | 7.186 | 30 | 0.24 |
| A/YUNNAN/0127/2015 vs A/TURKEY/EGYPT/137/2013 | 9.153 | 38 | 0.241 | A/TREE_SPARROW/INDONESIA/D10013/2010 vs A/TURKEY/EGYPT/137/2013 | 6.932 | 29 | 0.239 |
| A/DUCK/VIETNAM/OIE/2020/2012 vs A/CHICKEN/SICHUAN/J1/2014 | 7.845 | 33 | 0.238 | A/TURKEY/EGYPT/137/2013 vs A/CHICKEN/INDONESIA/D10014/2010 | 6.877 | 29 | 0.237 |
| A/DUCK/EGYPT/10565V/2010 vs A/DUCK/TAIWAN/1702004/2017 | 9.254 | 39 | 0.237 | A/DUCK/NANCHANG/9789/2013 vs A/CHICKEN/VIETNAM/NCVD/1192-2012 | 9.012 | 38 | 0.237 |
