# Supplementary material for: Mapping hemagglutinin residues driving antigenic diversity in H5Nx avian influenza viruses
Source: J Virol. 2026 Apr 30;100(6):e00095-26. doi: 10.1128/jvi.00095-26 (PMC13288987; doi:10.1128/jvi.00095-26)
Supplement: Table S4 — QuikChange Lightning site-directed mutagenesis primer pairs. [file jvi.00095-26-s0006.docx]

**Table S4:** QuikChange Lightning Site-directed Mutagenesis (Agilent) Primer Pairs.

| **Mutation (Amino Acid)** | **Forward Primer** | **Reverse Primer** |
| --- | --- | --- |
| 5 | cccaagacatactggaaagaacacacaacgggaagc | gcttcccgtggtggttttccagtagtcttggg |
| 40-43 | aaaaacacacaacgggagggctctgcaatctaaatggggtgaag | cttcaccccattagatggagagcctcccggttgtttt |
| 47 | gaagctctgcactctaaatgggattaagcctctgattattaaaggatt | aatccttaaaatcagaggcttatcccatttagtgcagagcttc |
| 54 | ggggtaagcctctgatttaaagaatttgagtgagtcgga | tccagctacatacaattctttaaaactagaggctccacc |
| 66 | gatggctcctcggaaacccattgtgcgatgaattc | gaattcatcgacaacagggtttccgaggagccatc |
| 71 | acccaatgtgcgatgaattcaccagagtgccggaat | attccggcactctggtgaattcatcgacaattgggt |
| 82 | gaatggtcttacatagggagaaggctaatccagctaat | attagctggattaggcttctcccatagtgacaccattc |
| 83 | ggtcattagctggattaggcttctcccatgtagaga | tcttacatagggagagactaatccagctaattagacc |
| 94-97 | gaccttgttacccagggacctcaatgactatgaagaactga | tcagttcttcagtagtcattgaggtccctggtaacagaggtc |
| 114-120 | gagcagaaataaatcatttgagaagactcggatcatccaccagaatcttggcccaaatcagaaacatcat | atgatgtttcatgattgggccaaagaatctggtggatgatccgagtcttcaaaatgatttatctgctc |
| 123 | aatgatgtttcatgattggtccagagaaactcttggggatg | catcccaaagagttttggaccaattcatgaaaacatcatt |
| 124 | ccccaagagttcttggcccagtcagaaactactag | ctaatgatgtttcatgactgggccaaagaactcttgggg |
| 126 | gagttcttggcccaaatcatacatattaggggtga | tcacccctagatgtatgattgggccaaagaactcc |
| 127-133 | gttcttgcccaaatcatgaagtatcatcaggggtgagctcagcttgtccatacc | ggtatggacaagctgagctcacccctgatgcatcttcatgattgggccaaagaac |
| 136-140 | attaggggtgagcgacgtgttcataccgtggaaatccctccttttc | gaaaaaggagggatttcccaggtatgaacaagctgcgtcacccctaat |
| 151-156 | tccttttcagaaatgtgatggcttccaaaagggacaatactaccccaataagataagctacaa | ttgtagcttattatttgggtatgtattgtctttttgggtaatgcatcagctttcgaaaagga |
| 161 | gaacgatgcatcccaataagagaggtcacaataataccaatcgggaaga | tcttcccgatggtattatgtgacgttctttttgggtaatgcatcgttc |
| 168-169 | taaagataagctacaaatactatcaggaagatctcttgtatatgtggg | ccccacgtatcaagagatctcctgataggtattattgtagcttatcttta |
| 184-185 | ggggattcatcattccaaacatgaagcagagcagacaaatatctataaaa | ttttatagagattgtcgtctgcttcattgtggaatgatgaaatcccc |
| 195-198 | agacaaatatctataaaaacccagccacctatgtttcagttggaacatcaacatta | taatgttgatgttccaaactgaaaataggttggggttttatagagatttgct |
| 204 | ttggtaccaattctggtttaatatgtgtccaaactgaaataag | ctatatttcagttggaacatcaacattaagagattggatccaa |
| 207-210 | gttggaacatcaacattaaacctgagattggagccaaaataagctcatagatccc | gggatctagtgactatttggctccaaatctcagttaatgttgatgttccaac |
| 223 | tcccaaagtaaacgggcaaggtggaagaatggactt | aagtccattcttccatttgcccgtttactggga |
| 229 | cggttttaaaattgtccagtagaagtccattttccacg | cgtggaagaatggacttcactggacaattttaaaaccg |
| 234-235 | ggaaagaatggacttcttggcaaatttaaagtcaaatgatgcaatcca | tggattgcatcatttgaactttgccagaagaatgcttctttcc |
| 275-277 | ggaatatggccactgcagcatgagtcaaaccccagtgag | ctactggggtttgacatctagctgcagtggccatattcc |
| 282 | cactaaatgtcaaaccccaaatagggcagataaaactctag | ctagagtttatcgtccttattggggtttgacatttagtg |
| 297 | ccattccacaatatacatcctatcaccatcgggg | ccccgagtggtaggatgtatatttgggaatgg |
| 309 | atgcccccaaaatacgtgaagtcagacaagttggtcc | ggaccaaacttgtctgacttcacgtattttggggcat |
