# Supplementary material for: Mapping hemagglutinin residues driving antigenic diversity in H5Nx avian influenza viruses
Source: J Virol. 2026 Apr 30;100(6):e00095-26. doi: 10.1128/jvi.00095-26 (PMC13288987; doi:10.1128/jvi.00095-26)
Supplement: Table S5 — Mean, standard deviation, and percent coefficient of variation of hemagglutination inhibition of putative antigenic residues. [file jvi.00095-26-s0007.docx]

**Table S5:** Mean, standard deviation (Std Dev) and percentage coefficient of variation (%CV) of haemagglutination inhibition (HI) of putative antigenic residues **(Figure 7)**.

| **Bird Number** | **K35R** | | | **K40R + D43N** | | | **V47I** | | | **D54N** | | | **M66L** | | | **I71T** | | |
| --- | --- | --- | --- | --- | --- | --- | --- | --- | --- | --- | --- | --- | --- | --- | --- | --- | --- | --- |
|  | **Mean** | **Std Dev** | **%CV** | **Mean** | **Std Dev** | **%CV** | **Mean** | **Std Dev** | **%CV** | **Mean** | **Std Dev** | **%CV** | **Mean** | **Std Dev** | **%CV** | **Mean** | **Std Dev** | **%CV** |
| 1242 | 0.00 | 0.00 | 0.00 | -0.33 | 0.24 | 17.75 | -1.00 | 0.00 | 0.00 | -1.00 | 0.00 | 0.00 | 0.00 | 0.00 | 0.00 | 0.00 | 0.00 | 0.00 |
| 1243 | -1.33 | 0.47 | 38.65 | -2.00 | 0.00 | 0.00 | -1.75 | 0.25 | 18.92 | -0.75 | 0.25 | 18.92 | -1.33 | 0.47 | 38.65 | -1.67 | 0.24 | 17.75 |
| 1244 | -0.67 | 0.47 | 38.65 | -1.33 | 0.24 | 17.75 | 0.00 | 0.00 | 0.00 | 0.00 | 0.00 | 0.00 | -2.00 | 0.00 | 0.00 | -2.00 | 0.00 | 0.00 |
| 1245 | -1.33 | 0.47 | 38.65 | -1.67 | 0.24 | 17.75 | -1.50 | 0.50 | 41.42 | -1.50 | 0.50 | 41.42 | -1.33 | 0.47 | 38.65 | -1.33 | 0.47 | 38.65 |
| 1247 | -1.00 | 0.00 | 0.00 | -0.33 | 0.24 | 17.75 | 0.00 | 0.00 | 0.00 | 0.00 | 0.00 | 0.00 | 0.00 | 0.00 | 0.00 | -1.67 | 0.24 | 17.75 |
| 1248 | -1.00 | 0.00 | 0.00 | -1.33 | 0.24 | 17.75 | -1.00 | 0.00 | 0.00 | 0.00 | 0.00 | 0.00 | -1.00 | 0.00 | 0.00 | -1.00 | 0.00 | 0.00 |
| 1249 | 0.00 | 0.00 | 0.00 | -0.33 | 0.24 | 17.75 | 0.00 | 0.00 | 0.00 | 0.00 | 0.00 | 0.00 | -0.67 | 0.24 | 17.75 | 0.00 | 0.00 | 0.00 |
| **Bird Number** | **R82K** | | | **A83T** | | | **S94N + N97D** | | | **I114T + L115R + …** | | | **P123T** | | | **N124S** | | |
|  | **Mean** | **Std Dev** | **%CV** | **Mean** | **Std Dev** | **%CV** | **Mean** | **Std Dev** | **%CV** | **Mean** | **Std Dev** | **%CV** | **Mean** | **Std Dev** | **%CV** | **Mean** | **Std Dev** | **%CV** |
| 1242 | -1.33 | 0.24 | 17.75 | -2.00 | 0.00 | 0.00 | -1.00 | 0.00 | 0.00 | -1.33 | 0.24 | 17.75 | -1.00 | 0.00 | 0.00 | -0.33 | 0.24 | 17.75 |
| 1243 | -2.33 | 0.24 | 17.75 | -2.50 | 0.00 | 0.00 | -1.67 | 0.24 | 17.75 | -2.00 | 0.00 | 0.00 | -1.50 | 0.00 | 0.00 | -1.67 | 0.24 | 17.75 |
| 1244 | -2.67 | 0.47 | 38.65 | -2.00 | 0.00 | 0.00 | -2.00 | 0.00 | 0.00 | -2.33 | 0.24 | 17.75 | -3.00 | 0.00 | 0.00 | -2.00 | 0.00 | 0.00 |
| 1245 | -2.33 | 0.24 | 17.75 | -2.00 | 0.00 | 0.00 | -1.67 | 0.24 | 17.75 | -2.33 | 0.47 | 38.65 | -1.00 | 0.00 | 0.00 | -1.67 | 0.24 | 17.75 |
| 1247 | -1.67 | 0.47 | 38.65 | -2.00 | 0.00 | 0.00 | -1.00 | 0.00 | 0.00 | -2.00 | 0.00 | 0.00 | -1.00 | 0.00 | 0.00 | -1.00 | 0.00 | 0.00 |
| 1248 | -2.67 | 0.47 | 38.65 | -3.00 | 0.00 | 0.00 | -2.00 | 0.00 | 0.00 | -1.67 | 0.47 | 38.65 | -2.00 | 0.00 | 0.00 | -1.00 | 0.00 | 0.00 |
| 1249 | -1.67 | 0.47 | 38.65 | -2.00 | 0.00 | 0.00 | -1.00 | 0.00 | 0.00 | -1.33 | 0.24 | 17.75 | -1.00 | 0.00 | 0.00 | -0.67 | 0.24 | 17.75 |
| **Bird Number** | **126Del** | | | **T127V + L129S + …** | | | **P136S + Q138L + …** | | | **I151T + N154D + …** | | | **K161E** | | | **N168Y + R169Q** | | |
|  | **Mean** | **Std Dev** | **%CV** | **Mean** | **Std Dev** | **%CV** | **Mean** | **Std Dev** | **%CV** | **Mean** | **Std Dev** | **%CV** | **Mean** | **Std Dev** | **%CV** | **Mean** | **Std Dev** | **%CV** |
| 1242 | -3.00 | 0.00 | 0.00 | -1.17 | 0.62 | 54.07 | -0.33 | 0.24 | 17.75 | -0.67 | 0.47 | 38.65 | 0.00 | 0.00 | 0.00 | 0.00 | 0.00 | 0.00 |
| 1243 | -2.75 | 0.25 | 18.92 | -1.67 | 1.03 | 103.84 | -2.67 | 0.24 | 17.75 | -2.17 | 0.47 | 38.65 | -1.00 | 0.00 | 0.00 | -1.00 | 0.00 | 0.00 |
| 1244 | 0.00 | 0.00 | 0.00 | 0.00 | 0.00 | 0.00 | -2.33 | 0.24 | 17.75 | -1.00 | 0.00 | 0.00 | 0.00 | 0.00 | 0.00 | -1.33 | 0.24 | 17.75 |
| 1245 | -3.50 | 0.50 | 41.42 | -2.50 | 0.50 | 41.42 | -2.67 | 0.24 | 17.75 | -1.50 | 0.41 | 32.71 | 0.00 | 0.00 | 0.00 | -1.67 | 0.24 | 17.75 |
| 1247 | 0.00 | 0.00 | 0.00 | -0.50 | 0.41 | 32.71 | -2.00 | 0.00 | 0.00 | -0.17 | 0.24 | 17.75 | -1.00 | 0.00 | 0.00 | -0.33 | 0.24 | 17.75 |
| 1248 | -0.50 | 0.00 | 0.00 | 0.00 | 0.00 | 0.00 | -2.00 | 0.00 | 0.00 | -1.00 | 0.00 | 0.00 | -1.00 | 0.00 | 0.00 | -1.00 | 0.00 | 0.00 |
| 1249 | -1.00 | 0.00 | 0.00 | -1.00 | 0.00 | 0.00 | -1.00 | 0.00 | 0.00 | 0.00 | 0.00 | 0.00 | -1.00 | 0.00 | 0.00 | -0.33 | 0.24 | 17.75 |
| **Bird Number** | **A184E + E185A** | | | **T195A + I198V** | | | **T204I** | | | **Q207L + V210E** | | | **R223S** | | | **F229Y** | | |
|  | **Mean** | **Std Dev** | **%CV** | **Mean** | **Std Dev** | **%CV** | **Mean** | **Std Dev** | **%CV** | **Mean** | **Std Dev** | **%CV** | **Mean** | **Std Dev** | **%CV** | **Mean** | **Std Dev** | **%CV** |
| 1242 | 0.00 | 0.00 | 0.00 | -0.67 | 0.47 | 38.65 | -2.00 | 0.00 | 0.00 | -1.00 | 0.00 | 0.00 | -1.00 | 0.00 | 0.00 | -2.00 | 0.00 | 0.00 |
| 1243 | -2.67 | 0.24 | 17.75 | -1.33 | 0.24 | 17.75 | -3.50 | 0.00 | 0.00 | -1.67 | 0.24 | 17.75 | -1.67 | 0.24 | 17.75 | -2.50 | 0.00 | 0.00 |
| 1244 | -1.17 | 0.24 | 17.75 | -1.67 | 0.47 | 38.65 | -2.00 | 0.00 | 0.00 | -2.00 | 0.00 | 0.00 | -1.00 | 0.00 | 0.00 | -2.00 | 0.00 | 0.00 |
| 1245 | -1.33 | 0.47 | 38.65 | -2.00 | 0.00 | 0.00 | -2.00 | 0.00 | 0.00 | -1.33 | 0.47 | 38.65 | -1.33 | 0.47 | 38.65 | -2.00 | 0.00 | 0.00 |
| 1247 | 0.00 | 0.00 | 0.00 | -1.33 | 0.24 | 17.75 | -2.00 | 0.00 | 0.00 | -1.00 | 0.00 | 0.00 | -1.00 | 0.00 | 0.00 | -2.00 | 0.00 | 0.00 |
| 1248 | -0.50 | 0.41 | 32.71 | -1.33 | 0.24 | 17.75 | -3.00 | 0.00 | 0.00 | -1.00 | 0.00 | 0.00 | -0.33 | 0.47 | 38.65 | -3.00 | 0.00 | 0.00 |
| 1249 | -0.50 | 0.41 | 32.71 | 0.00 | 0.00 | 0.00 | -3.00 | 0.00 | 0.00 | -0.50 | 0.41 | 32.71 | -0.33 | 0.47 | 38.65 | -2.00 | 0.00 | 0.00 |
| **Bird Number** | **K234R + P235S** | | | **N275S + K277R** | | | **V282I** | | | **L297I** | | | **N309D** | | |  |  |  |
|  | **Mean** | **Std Dev** | **%CV** | **Mean** | **Std Dev** | **%CV** | **Mean** | **Std Dev** | **%CV** | **Mean** | **Std Dev** | **%CV** | **Mean** | **Std Dev** | **%CV** |  |  |  |
| 1242 | -1.00 | 0.00 | 0.00 | -0.17 | 0.24 | 17.75 | -0.17 | 0.24 | 17.75 | -0.17 | 0.24 | 17.75 | -0.17 | 0.24 | 17.75 |  |  |  |
| 1243 | -2.00 | 0.41 | 32.71 | -1.67 | 0.24 | 17.75 | -1.67 | 0.24 | 17.75 | -1.67 | 0.24 | 17.75 | -1.83 | 0.24 | 17.75 |  |  |  |
| 1244 | -2.00 | 0.00 | 0.00 | -2.00 | 0.00 | 0.00 | -3.00 | 0.00 | 0.00 | -2.00 | 0.00 | 0.00 | -1.00 | 0.00 | 0.00 |  |  |  |
| 1245 | -1.67 | 0.47 | 38.65 | -1.50 | 0.41 | 32.71 | -1.83 | 0.24 | 17.75 | -2.33 | 0.47 | 38.65 | -1.50 | 0.41 | 32.71 |  |  |  |
| 1247 | -1.33 | 0.47 | 38.65 | -1.00 | 0.00 | 0.00 | -1.83 | 0.24 | 17.75 | -1.83 | 0.24 | 17.75 | -1.17 | 0.24 | 17.75 |  |  |  |
| 1248 | -2.33 | 0.47 | 38.65 | -2.00 | 0.00 | 0.00 | -1.83 | 0.24 | 17.75 | -2.00 | 0.00 | 0.00 | -1.17 | 0.24 | 17.75 |  |  |  |
| 1249 | -1.33 | 0.47 | 38.65 | -0.17 | 0.24 | 17.75 | -1.83 | 0.24 | 17.75 | -1.00 | 0.00 | 0.00 | -1.17 | 0.24 | 17.75 |  |  |  |
