# Supplementary material for: Mapping hemagglutinin residues driving antigenic diversity in H5Nx avian influenza viruses
Source: J Virol. 2026 Apr 30;100(6):e00095-26. doi: 10.1128/jvi.00095-26 (PMC13288987; doi:10.1128/jvi.00095-26)
Supplement: Table S6 — Two-paired p-value of comparison of homologous and heterologous virus antisera. [file jvi.00095-26-s0008.docx]

**Table S6:** Two-paired p-value of comparison of homologous and heterologous virus antisera. The Wilcoxon test statistic was applied to non-zero paired differences of log2-transformed HI titres. Cells are coloured according to significance: p>0.05 (blank), p<0.05 (lavender), p<0.01 (blue), p<0.001 (green). Cells where all differences were zero are marked as “N/A”.

|  | **NPL14** | **BGD11** | **IDN10a** | **CHN15b** | **USA15** | **VMN12a** | **EGY13** | **EGY15** | **EGY10** | **CHN13** | **VMN12b** | **IDN10b** | **VMN12c** | **CHN15a** | **RUS18** | **IRN17** | **CHN14** | **CHN15b** | **TWN17** | **CHN16** | **VMN20** | **CHN21** |
| --- | --- | --- | --- | --- | --- | --- | --- | --- | --- | --- | --- | --- | --- | --- | --- | --- | --- | --- | --- | --- | --- | --- |
| **NPL14** |  | 0.043 | 0.028 | 0.028 | 0.028 | 0.028 | 0.109 | 0.028 | 0.010 | 0.011 | 0.028 | 0.068 | 0.068 | 0.028 | 0.068 | 0.068 | 0.028 | 0.043 | 0.028 | 0.068 | 0.028 | 0.028 |
| **BGD11** | 0.028 |  | 0.028 | 0.028 | 0.028 | 0.028 | 0.000 | 0.028 | 0.043 | 0.028 | 0.028 | 0.068 | 0.068 | 0.028 | 0.068 | 0.068 | 0.028 | 0.043 | 0.028 | 0.068 | 0.028 | 0.028 |
| **IDN10a** | 0.028 | 0.043 |  | 0.028 | 0.028 | 0.028 | 0.028 | 0.028 | 0.028 | 0.028 | N/A | 0.068 | 0.068 | 0.028 | 0.068 | 0.109 | 0.028 | 0.043 | 0.028 | 0.068 | 0.028 | 0.028 |
| **CHN15b** | 0.028 | 0.043 | 0.028 |  | 0.028 | 0.028 | 0.028 | 0.028 | 0.028 | 0.043 | 0.028 | 0.068 | 0.068 | N/A | 0.068 | 0.068 | 0.028 | 0.000 | 0.001 | 0.068 | 0.028 | 0.028 |
| **USA15** | 0.028 | 0.043 | 0.028 | 0.028 |  | 0.028 | 0.001 | 0.028 | 0.028 | 0.043 | 0.028 | 0.180 | 0.068 | N/A | N/A | N/A | N/A | 0.008 | 0.000 | 0.317 | N/A | 0.028 |
| **VMN12a** | 0.028 | 0.043 | 0.028 | 0.028 | 0.028 |  | 0.028 | 0.028 | 0.028 | 0.028 | 0.028 | 0.109 | 0.068 | 0.028 | 0.068 | 0.068 | 0.028 | 0.043 | 0.028 | 0.068 | 0.028 | 0.028 |
| **EGY13** | 0.028 | 0.043 | 0.028 | 0.028 | 0.028 | 0.028 |  | 0.028 | 0.022 | 0.018 | 0.028 | 0.068 | 0.068 | 0.028 | 0.068 | 0.068 | 0.028 | 0.043 | 0.028 | 0.068 | 0.028 | 0.028 |
| **EGY15** | 0.028 | 0.043 | 0.028 | 0.028 | 0.028 | 0.028 | N/A |  | 0.000 | 0.011 | 0.028 | 0.068 | 0.068 | 0.028 | 0.068 | 0.068 | 0.028 | 0.043 | 0.028 | 0.068 | 0.028 | 0.028 |
| **EGY10** | 0.028 | 0.043 | 0.028 | 0.028 | 0.028 | 0.028 | 0.000 | 0.028 |  | 0.028 | 0.028 | 0.068 | 0.068 | 0.028 | 0.068 | 0.068 | 0.028 | 0.043 | 0.028 | 0.068 | 0.028 | 0.028 |
| **CHN13** | 0.043 | 0.043 | 0.028 | 0.028 | 0.028 | 0.028 | 0.028 | 0.028 | 0.022 |  | 0.028 | 0.068 | 0.068 | 0.028 | 0.068 | 0.068 | 0.028 | 0.043 | 0.028 | 0.068 | 0.028 | 0.028 |
| **VMN12b** | 0.028 | 0.043 | 0.028 | 0.028 | 0.028 | 0.028 | 0.028 | 0.028 | 0.028 | 0.028 |  | 0.068 | 0.068 | 0.028 | 0.068 | 0.109 | 0.028 | 0.043 | 0.028 | 0.068 | 0.028 | 0.028 |
| **IDN10b** | 0.028 | 0.043 | 0.000 | 0.028 | 0.028 | 0.028 | 0.043 | 0.028 | 0.028 | 0.028 | 0.028 |  | 0.068 | 0.028 | 0.068 | 0.068 | 0.028 | 0.043 | 0.028 | 0.068 | 0.028 | 0.028 |
| **VMN12c** | 0.002 | 0.043 | 0.028 | 0.028 | 0.028 | 0.028 | 0.001 | 0.028 | 0.010 | 0.018 | 0.028 | 0.068 |  | 0.028 | 0.068 | 0.068 | 0.028 | 0.043 | 0.028 | 0.068 | 0.028 | 0.028 |
| **CHN15a** | 0.028 | 0.043 | 0.028 | 0.028 | 0.028 | 0.028 | 0.028 | 0.028 | 0.028 | 0.068 | 0.028 | 0.068 | 0.068 |  | 0.068 | 0.068 | 0.028 | 0.068 | 0.028 | 0.068 | 0.028 | 0.028 |
| **RUS18** | 0.281 | 0.109 | 0.000 | 0.000 | N/A | 0.000 | 0.000 | 0.003 | 0.000 | 0.003 | 0.043 | 0.109 | 0.068 | 0.000 |  | N/A | 0.043 | 0.000 | 0.000 | 0.317 | 0.000 | 0.028 |
| **IRN17** | 0.080 | 0.043 | 0.028 | 0.028 | 0.028 | 0.028 | 0.010 | 0.028 | 0.022 | 0.028 | 0.028 | 0.068 | 0.068 | 0.028 | 0.068 |  | 0.028 | 0.043 | 0.028 | 0.068 | 0.109 | 0.028 |
| **CHN14** | 0.028 | 0.043 | 0.028 | 0.028 | 0.028 | 0.028 | 0.028 | 0.028 | 0.028 | 0.028 | 0.028 | 0.068 | 0.068 | 0.028 | 0.068 | 0.109 |  | 0.043 | 0.028 | 0.068 | 0.043 | 0.028 |
| **CHN15b** | 0.028 | 0.043 | 0.028 | 0.028 | 0.028 | 0.028 | 0.028 | 0.028 | 0.028 | 0.068 | 0.028 | 0.068 | 0.068 | 0.028 | 0.068 | 0.068 | 0.028 |  | 0.028 | 0.068 | 0.028 | 0.028 |
| **TWN17** | 0.028 | 0.043 | 0.028 | 0.028 | 0.028 | 0.028 | 0.028 | 0.028 | 0.028 | 0.068 | 0.028 | 0.068 | 0.068 | 0.028 | 0.068 | 0.068 | 0.028 | 0.068 |  | 0.068 | 0.028 | 0.028 |
| **CHN16** | 0.028 | 0.043 | 0.028 | 0.028 | 0.028 | 0.028 | 0.028 | 0.028 | 0.028 | 0.043 | 0.028 | 0.068 | 0.068 | 0.028 | 0.068 | 0.068 | 0.028 | 0.043 | 0.028 |  | 0.028 | 0.028 |
| **VMN20** | 0.028 | 0.043 | 0.028 | 0.028 | 0.028 | 0.028 | 0.028 | 0.028 | 0.028 | 0.043 | 0.028 | 0.068 | 0.068 | 0.028 | 0.068 | 0.068 | 0.317 | 0.043 | 0.028 | 0.068 |  | 0.028 |
| **CHN21** | 0.028 | 0.043 | 0.028 | 0.028 | 0.028 | 0.028 | 0.028 | 0.028 | 0.022 | 0.028 | 0.028 | 0.068 | 0.068 | 0.028 | 0.068 | 0.068 | 0.028 | 0.043 | 0.028 | 0.068 | 0.028 |  |
